# Comparing human papillomavirus prevalences in women with normal cytology or invasive cervical cancer to rank genotypes according to their oncogenic potential: a meta-analysis of observational studies

**DOI:** 10.1186/1471-2334-13-373

**Published:** 2013-08-13

**Authors:** Erik Bernard, Margarita Pons-Salort, Michel Favre, Isabelle Heard, Elisabeth Delarocque-Astagneau, Didier Guillemot, Anne CM Thiébaut

**Affiliations:** 1 Institut Pasteur, Unité de Pharmaco-Épidémiologie et Maladies Infectieuses, 25-28 rue du Dr Roux, 75724 Paris Cedex 15 France; 2 INSERM, U657, 25-28 rue du Dr Roux, 75724 Paris Cedex 15 France; 3 Univ Versailles–St-Quentin-en-Yvelines, EA 4499, UFR des Sciences de la Santé Simone Veil, 2 avenue de la Source de la Bièvre, 78180 Montigny Le Bretonneux France; 4 UPMC Univ Paris 06, Cellule Pasteur UPMC, 25-28 rue du Dr Roux, 75724 Paris Cedex 15 France; 5 Institut Pasteur, Centre National de Référence des HPV, 25-28 rue du Dr Roux, 75724 Paris Cedex 15 France; 6 Institut Pasteur, Genetics, Papillomavirus and Human Cancer Unit, 25-28 rue du Dr Roux, 75724 Paris Cedex 15 France; 7 UPMC Univ Paris 06, UMRS 943, Groupe Hospitalier Pitié–Salpêtrière, 91-105 boulevard de l’Hôpital, 75013 Paris, France; 8 INSERM, U943, Groupe Hospitalier Pitié–Salpêtrière, 91-105 boulevard de l’Hôpital, 75013 Paris, France; 9 AP–HP, Hôpital Raymond Poincaré, 104 boulevard Raymond Poincaré, 92380 Garches, France

**Keywords:** Human papillomavirus, Genotype, Cervical cancer, Oncogenic potential, Meta-analysis

## Abstract

**Background:**

Mucosal human papillomavirus (HPV) infection is a necessary cause of cervical cancer. Vaccine and non-vaccine genotype prevalences may change after vaccine introduction. Therefore, it appears essential to rank HPV genotypes according to their oncogenic potential for invasive cervical cancer, independently of their respective prevalences.

**Methods:**

We performed meta-analyses of published observational studies and estimated pooled odds ratios with random-effects models for 32 HPV genotypes, using HPV-16 as the reference.

**Results:**

Twenty-seven studies yielded 9,252 HPV-infected women: 2,902 diagnosed with invasive cervical cancer and 6,350 with normal cytology. Expressed as (odds ratio [95% confidence interval]), HPV-18 (0.63 [0.51, 0.78]) ranked closest to HPV-16, while other genotypes showed continuously decreasing relative oncogenic potentials: HPV-45 (0.35 [0.22, 0.55]), HPV-69 (0.28 [0.09, 0.92]), HPV-58 (0.24 [0.15, 0.38]), HPV-31 (0.22 [0.14, 0.35]), HPV-33 (0.22 [0.12, 0.38]), HPV-34 (0.21 [0.06, 0.80]), HPV-67 (0.21 [0.06, 0.67]), HPV-39 (0.17 [0.09, 0.30]), HPV-59 (0.17 [0.09, 0.31]), HPV-73 (0.16 [0.06, 0.41]), and HPV-52 (0.16 [0.11, 0.23]).

**Conclusions:**

Our results support the markedly higher oncogenic potentials of HPV-16 and -18, followed by HPV-31, -33, -39, -45, -52, -58 and -59, and highlight the need for further investigation of HPV-34, -67, -69 and -73. Overall, these findings could have important implications for the prevention of cervical cancer.

## Background

Invasive cervical cancer (ICC) is the third most common cancer among women worldwide, with an estimated incidence of 553,119 new cases and 288,109 deaths in 2010 [1]. Persistent infection with one of the oncogenic human papillomavirus (HPV) genotypes is required to cause ICC [2-5]. More than 150 HPV genotypes have been identified and about 40 are known to infect the genital tract [6,7].

To date, HPV genotypes identified as causing ICC have belonged to a few genetically related “high-risk” species of the mucosotropic α-genus (α-5, -6, -7, -9 and -11) [8] but other HPV genotypes could be involved [9]. Dichotomous classification into low- and high-risk HPV genotypes has often been used previously [4,10]. Alternatively, the International Agency for Research on Cancer (IARC) classified individual HPV genotypes into more categories based on available epidemiologic and mechanistic evidence of their carcinogenicity for cancer at any site. Thus, 12 HPV genotypes (HPV-16, -18, -31, -33, -35, -39, -45, -51, -52, -56, -58 and -59) are classified as “carcinogenic to humans” (Group 1), HPV-68 as “probably carcinogenic” (Group 2A) and 12 other HPV genotypes as “possibly carcinogenic” (Group 2B) [8,11].

Two vaccines targeting HPV-16 and -18, which account for 70% of cervical cancers worldwide [12,13], are currently available. Vaccination impact on the cervical cancer incidence remains uncertain, especially because genotype-specific prevalences of vaccine and non-vaccine genotypes might change after vaccine introduction through vaccine-induced cross-protection or genotype replacement [14-16]. The number of ICC cases associated with a given HPV genotype depends both on its prevalence in the general population and its oncogenic potential, which can be defined as the inherent and differential abilities of each genotype to trigger malignant transformation and induce cervical cancer [17]. Within categories of IARC-classified carcinogenic HPV genotypes, the risk of progression to ICC might differ by HPV genotype. Therefore, ranking the oncogenic potentials of HPV genotypes, independently of their respective prevalences, is challenging but essential to guide the formulation of second-generation polyvalent HPV vaccines and HPV-DNA–based screening tests.

This study was undertaken to rank HPV genotypes as causal agents of ICC according to their relative oncogenic potentials assessed by means of meta-analyses of published observational data. Oncogenic potentials herein are expressed using HPV-16 as the reference, since it has been recognized as the most carcinogenic HPV genotype [8,11,18].

## Methods

### Literature search and study selection

Original studies published in English, French, German and Spanish from 1/1995 to 3/2011 were systematically sought in PubMed/Medline and Embase databases, in March 2011. The following keywords were combined: “female”, “papillomavirus infection”, “DNA probes, HPV”, “DNA, viral”, “genotype”, “polymerase chain reaction”, “sequence analysis, DNA”, “uterine cervical neoplasms”, “cervix uteri”, “epidemiologic studies”, “prevalence”, “incidence” (Additional file 1). We restricted our selection to original articles (reviews, meta-analyses, editorials, comments and letters were not eligible). Conference abstracts and other unpublished articles were not considered.

First, article titles and abstracts were screened then full texts were read to check inclusion criteria. The relevance of references cited in the retrieved articles, reviews and meta-analyses was also evaluated for potential inclusions. When necessary, authors were contacted for confirmation of inclusion criteria or results.

Unvaccinated women of any age were considered for this meta-analysis. We defined the following three inclusion criteria: prevalence data for at least one HPV genotype other than HPV-16 and -18; inclusion of ≥20 HPV-infected women with ICC and ≥20 HPV-infected women with normal cytology, and HPV-prevalence data for women with ICC presented separately from those with normal cytology.

One author (EB) conducted the eligibility assessment and problematic papers were resolved by collegial discussion (MPS, ACMT). BibDesk 1.5.4 software was used to manage references.

### Data extraction

For each study, one author (EB) extracted the following data, entering them into a predefined Excel spreadsheet: study characteristics (first author, year of publication, journal, country and continent, design and funding source), characteristics of included subjects (number of cases [total and by histologic type, if available] and controls, age data), methods (cytologic or histologic cervical specimen, identification and typing method, primers used [if any] and number of HPV genotypes detectable) and results (numbers of HPV genotypes actually identified, and simple and multiple infections, genotype-specific HPV-prevalence data for cases and controls). For multiple infections with ≥2 HPV genotypes, no weighting was used and each HPV genotype was counted equally. Infection with an uncharacterized HPV genotype is denoted HPV-X.

Furthermore, study quality was assessed with a list of specifically defined criteria, inspired by some of the STROBE checklist items [19]. The following items were coded yes, no, unclear or missing: comparability of cases and controls for geographic origin, age, sample type and methods used to detect and genotype HPV; blinded assessment; numbers of individuals reported at each study stage; and genotype prevalences reported for multiple infections. Data extraction was double checked by two authors (MPS, ACMT).

### Statistical analyses

A meta-analysis was performed for each HPV genotype, after excluding studies that did not seek or report the genotype under consideration and those that sought it but reported zero cases and controls. Therefore, the number of studies analyzed varied from one genotype to another. We did not consider genotypes for which all but one study reported zero controls. For each study and each available genotype, an odds ratio (OR) and its 95% confidence interval (CI) were computed from the reported numbers of case and control infections, considering HPV-16 infections as the reference group. Then, each HPV genotype was subjected to meta-analysis across the corresponding number of studies by combining the studies’ ORs using DerSimonian and Laird’s random-effects model [20]. If a count equalled zero when cross-tabulating case–control status and infection with a given HPV genotype or HPV-16 (usually no controls infected with the HPV genotype under consideration), we applied a continuity correction (CC) by adding 0.5 to each cell [21,22].

For each HPV genotype, heterogeneity of the estimated oncogenic potentials relative to HPV-16 was assessed graphically with forest plots, and quantitatively using Cochran’s Q-test and the I^2^ statistic [23,24]. When Cochran’s Q-test was statistically significant at the 10% level or the I^2^ statistic ≥50%, we examined potential sources of heterogeneity by performing subgroup analyses according to five prespecified factors: study design (case–control versus cross-sectional), year of publication (before and after the median, ≤2005 versus >2005), comparability of case and control ages (similar versus unbalanced distribution or unclear information), geographic area (Asia [18,25] versus all other continents), and HPV-detection level among cases (<90% versus ≥90% threshold). For each HPV genotype, we assessed publication bias (or other potential sources of bias) by examining the funnel plots for asymmetry and running the Egger test [26].

Sensitivity analyses were performed using: a fixed-effect model, with Peto’s method [27], CC = 0.25 or 0, and HPV-negative subjects as the reference group.

All statistical analyses were computed using the package Meta-analysis in Stata in Stata/SE v11.0 [28,29].

## Results

### Study identification and description

**Figure 1:**
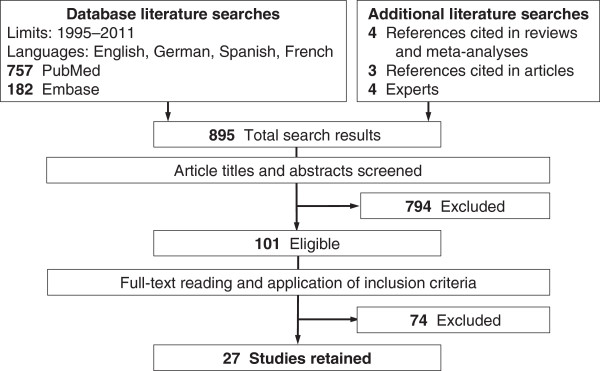
Article identification and selection process for inclusion in the meta-analysis.

The Medline and Embase database searches provided, respectively, 757 and 182 references, while additional searches identified 11 studies, yielding, after deleting 55 duplicates, a total of 895 references (Figure 1). Among them, 794 were excluded based on their titles and abstracts. The full texts of the remaining 101 references were read and 27 studies fully satisfying the inclusion criteria were finally retained [30-56]: 17 case–control and 10 cross-sectional studies, published between 1997 and 2011, all but one (Spanish [30]) written in English. They had been conducted on four continents: Asia (12 studies), Europe (six studies), South and Central America (five studies), and Africa (four studies). A total of 3,191 women with ICC (cases) and 29,623 with normal cytology (controls) were included (Table 1).

**Table 1:** Characteristics of studies, numbers of women with invasive cervical cancer (cases) or normal cytology (controls)

**First author, year [reference]**	**Country**	**Study design**	**Cases, n**		**Controls, n**	
			**HPV+**	**Total**	**HPV+**	**Total**
Abba, 2003 [30]	Argentina	Cross-sectional	21	21	70	152
Alibegashvili, 2011 [31]	Georgia	Case–control	91	91	143	1,247
An, 2003 [32]	South Korea	Cross-sectional	48	50	72	276
Andersson, 2005 [33]	Sweden	Cross-sectional	45	45	24	24
Asato, 2004 [34]	Japan	Case–control	311	356	333	3,249
Baay, 2001 [35]	Belgium	Case–control	101	115	31	286
Bardin, 2008 [36]	Poland	Case–control	87	88	115	799
Castellsagué, 2008 [37]	Mozambique	Case–control	230	241	148	195
Chang, 1997 [38]	China	Case–control	39	47	42	72
Chaouki, 1998 [39]	Morocco	Case–control	144	152	38	185
Cho, 2003 [40]	South Korea	Cross-sectional	43	49	132	414
Ferrera, 1999 [41]	Honduras	Case–control	83	104	170	438
Hammouda, 2011 [42]	Algeria	Case–control	167	171	39	732
Herrero, 2005 [43]	Costa-Rica	Cross-sectional	34	35	1,671	7,459
Hong, 2008 [44]	China	Case–control	172	181	91	217
Illades-Aguiar, 2009 [45]	Mexico	Case–control	133	133	91	256
Illades-Aguiar, 2010 [46]	Mexico	Cross-sectional	141	141	1,274	3,117
Keita, 2009 [47]	Guinea	Case–control	70	77	360	752
Lee, 2007 [48]	South Korea	Cross-sectional	133	160	388	1,650
Liu, 2010 [49]	China	Case–control	111	134	274	613
Maehama, 2005 [50]	Japan	Case–control	330	383	434	4,078
Park, 2004 [51]	South Korea	Cross-sectional	59	62	51	290
Sasagawa, 2001 [52]	Japan	Case–control	75	84	151	1,562
Sherpa, 2010 [53]	Nepal	Case–control	54	61	73	898
Tachezy, 1999 [54]	Czech Republic	Cross-sectional	36	49	38	165
Tornesello, 2006 [55]	Italy	Case–control	53	65	36	183
Wu, 2010 [56]	China	Cross-sectional	91	96	61	314
Total			2,902	3,191	6,350	29,623

HPV+, human papillomavirus-positive.

When available, mean age ranges were 44–56 years for cases and 32–52 years for controls. Case and control ages were comparable in five studies but not in eight others, and this information was unclear or missing in the 14 other papers. The cervical specimens used to detect HPV infection were usually cytologic for controls (23 studies) and histologic for cases (13 studies), with specimen type being similar for cases and controls in 14 studies. All studies used polymerase chain reaction (PCR) to detect HPV infection and 4–48 HPV genotypes could be identified in each study (4–20 in cases, 4–36 in controls). HPV infection was detected in 73–100% of cases (squamous cell carcinoma: 80–100%; adenocarcinoma: 50–100%) and 5–76% of controls (except in [33] which included only 24 controls, all HPV-positive).

### Estimation of the genotype-specific oncogenic potential compared to HPV-16

In total, 9,252 HPV-infected women (2,902 cases and 6,350 controls) were included (Table 1). Among HPV-positive subjects, multiple infections were more frequent in women with normal cytology (16% on average and up to 50%) than those diagnosed with ICC (10% on average and up to 35%). A total of 3,150 HPV infections, including multiple infections, were identified among the 2,902 cases. The most common HPV genotypes identified in women with ICC were, in descending order: HPV-16 (57.9%), HPV-18 (12.8%), HPV-45 (4.8%), HPV-58 (4.7%), HPV-33 (4.7%), HPV-31 (4.4%) and HPV-52 (4.0%). Prevalences of the other HPV genotypes were <4% (Table 2). The overall HPV-X prevalence was 7.6% but this value represents different numbers of genotypes from one study to another.

**Table 2:** Human papillomavirus genotype-specific prevalences among invasive cervical cancer cases, meta-analytical estimates of relative oncogenic potentials

**HPV-**	Prevalence*		**Cases**^**†**^**, n**	**Pooled OR**	**95% CI**	**Studies**^**‡**^**, n**	**Cochran’s Q *- *test P**	**I**^**2 **^**statistic, %**	**Between-study variance**
	**n**	**(%)**							
6	17	(0.8)	2,208	0.08	0.04, 0.16	18	0.162	24.8	0.438
11	17	(1.1)	1,554	0.11	0.06, 0.19	15	0.407	4.1	0.049
16	1,680	(57.9)	2,902		Reference	27			
18	372	(12.8)	2,902	0.63	0.51, 0.78	27	0.425	2.6	0.009
30	2	(0.5)	430	0.13	0.03, 0.60	3	0.881	0.0	0.000
31	122	(4.4)	2,745	0.22	0.14, 0.35	24	<0.001	55.9	0.696
33	128	(4.7)	2,728	0.22	0.12, 0.38	24	<0.001	65.1	1.152
34	1	(0.2)	666	0.21	0.06, 0.80	5	0.791	0.0	0.000
35	67	(2.7)	2,450	0.12	0.08, 0.17	21	0.408	4.0	0.032
39	24	(1.2)	2,017	0.17	0.09, 0.30	19	0.169	23.7	0.372
40	2	(0.2)	991	0.13	0.04, 0.45	8	0.197	28.9	0.889
42	3	(0.2)	1,573	0.05	0.02, 0.15	11	0.102	37.2	1.345
44	3	(0.4)	729	0.14	0.03, 0.66	5	0.147	41.1	1.324
45	119	(4.8)	2,464	0.35	0.22, 0.55	23	0.054	34.4	0.358
51	54	(2.7)	1,971	0.10	0.05, 0.20	19	0.014	46.3	0.901
52	96	(4.0)	2,394	0.16	0.11, 0.23	21	0.293	12.7	0.091
53	22	(1.1)	1,981	0.07	0.04, 0.12	16	0.380	6.4	0.075
54	1	(0.1)	1,263	0.06	0.02, 0.16	9	0.580	0.0	0.000
56	22	(1.0)	2,303	0.09	0.05, 0.16	20	0.278	14.2	0.230
58	127	(4.7)	2,685	0.24	0.15, 0.38	22	<0.001	56.8	0.583
59	24	(1.1)	2,218	0.17	0.09, 0.31	16	0.537	0.0	0.000
61	2	(0.2)	1,130	0.05	0.02, 0.14	8	0.739	0.0	0.000
62	1	(0.2)	558	0.07	0.02, 0.23	6	0.420	0.0	0.000
66	16	(0.7)	2,181	0.08	0.05, 0.14	18	0.799	0.0	0.000
67	4	(0.3)	1,342	0.21	0.06, 0.67	10	0.181	28.6	1.020
68	8	(0.5)	1,661	0.07	0.04, 0.14	14	0.439	0.8	0.013
69	9	(1.3)	672	0.28	0.09, 0.92	5	0.249	25.8	0.473
70	6	(0.4)	1,496	0.07	0.03, 0.14	11	0.565	0.0	0.000
71	1	(0.1)	672	0.03	0.01, 0.13	4	0.527	0.0	0.000
73	8	(0.7)	1,151	0.16	0.06, 0.41	10	0.617	0.0	0.000
74	1	(0.4)	264	0.10	0.00, 3.41	2	0.051	73.8	4.874
81	1	(0.1)	1,257	0.04	0.02, 0.10	11	0.966	0.0	0.000
82	4	(0.4)	892	0.13	0.04, 0.36	7	0.939	0.0	0.000

*HPV prevalence among cases, with no distinction between simple and multiple infections, across the studies that reported counts of the HPV genotypes under consideration. ^†^Number of cases tested for the given HPV genotype. ^‡^Number of studies included in each meta-analysis. *Abbreviations*: *HPV* human papilloma virus, *OR* odds ratio, *CI* confidence interval.

**Figure 2:**
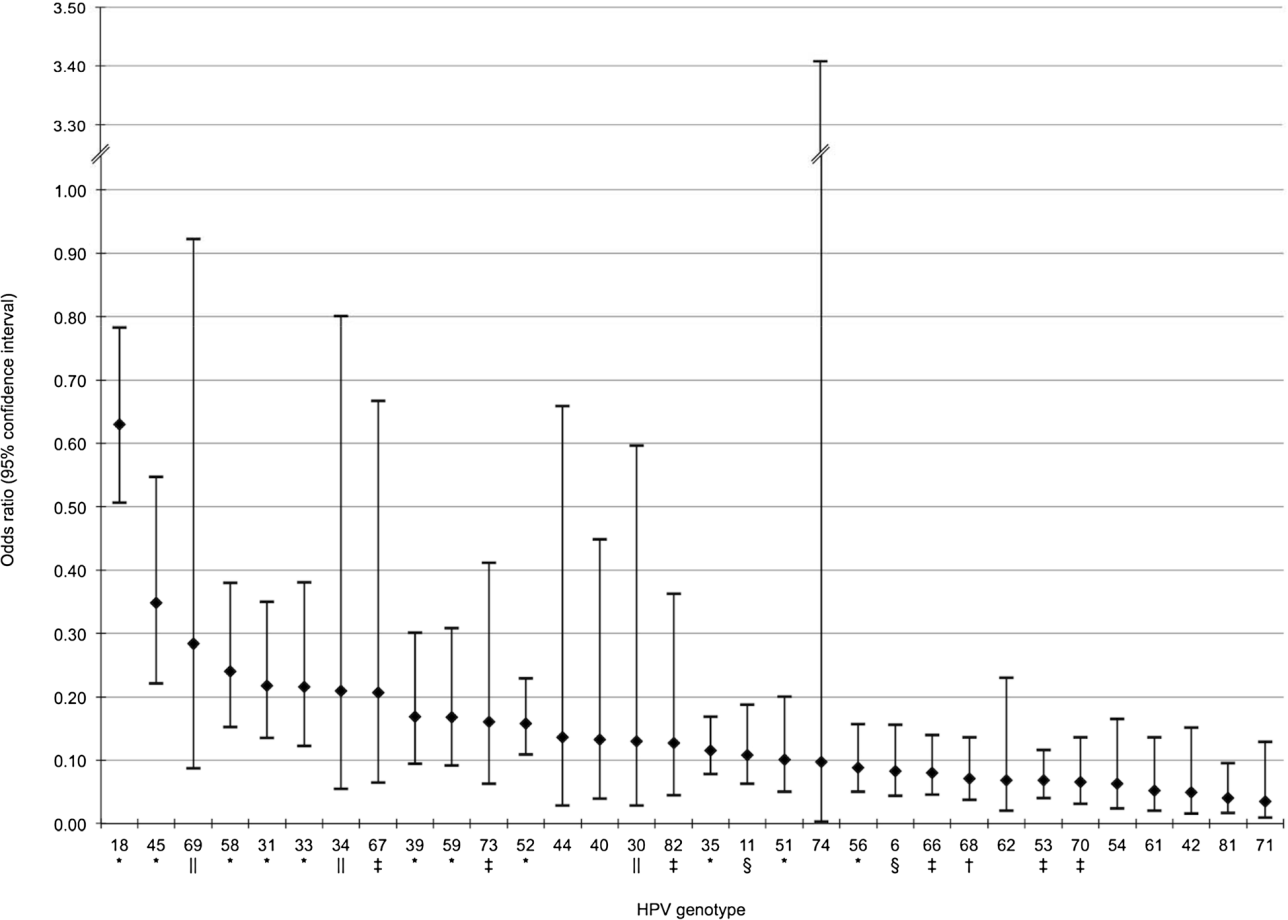
**Pooled odds ratios estimating the oncogenic potential of each HPV genotype relative to HPV-16.** NOTE: HPV genotypes were classified according to the International Agency for Research on Cancer [8,11], as follows: *Carcinogenic (Group 1), †probably carcinogenic (Group 2A), ‡possibly carcinogenic based on limited evidence in humans (Group 2B), ||possibly carcinogenic based on phylogenetic analogy to HPV genotypes with sufficient or limited evidence in humans (Group 2B), and §unclassifiable (Group 3). HPV-6, -11, -16, -18, -31, -33, -45, -52 and **-**58 are putatively included in the future nonavalent anti-HPV vaccine. Precise point estimates and 95% confidence interval limits illustrated in this figure are available in Table 2, columns 5 and 6.

Available data enabled assessment of the relative oncogenic potentials of 32 HPV genotypes (Table 2). Each meta-analysis included two (HPV-74) to 27 (HPV-18) studies (forest plots in Additional file 2). All pooled ORs were statistically significantly <1, except for HPV-74 (two-sided P = 0.20, calculated from two studies, one of which provided only one case, Figure 2). HPV-18 had the highest OR (0.63; 95% CI, [0.51, 0.78]); HPV-45 the second highest (0.35 [0.22, 0.55]) followed closely by the others, in descending order: HPV-69 (0.28 [0.09, 0.92]), HPV-58 (0.24 [0.15, 0.38]), HPV-31 (0.22 [0.14, 0.35]), HPV-33 (0.22 [0.12, 0.38]), HPV-34 (0.21 [0.06, 0.80]), HPV-67 (0.21 [0.06, 0.67]), HPV-39 (0.17 [0.09, 0.30]), HPV-59 (0.17 [0.09, 0.31]), HPV-73 (0.16 [0.06, 0.41]), and HPV-52 (0.16 [0.11, 0.23]). The ORs for the remaining HPV genotypes were <0.15.

### Heterogeneity and bias assessment

Cochran’s Q-test suggested heterogeneity for six HPV genotypes: HPV-31, -33, -45, -51, -58 and -74; and the I^2^ statistic for four among them: HPV-31, -33, -58 and -74 (Table 2). This heterogeneity was not clearly explained by any of the factors considered (study design, year of publication, geographic area, age-distribution balance between cases and controls, or HPV-detection rate among cases). For example, the I^2^ statistic was smaller for cross-sectional studies than case–control studies for HPV-33, -51 and -58, but higher for HPV-31 and -45. For HPV-74, subgroup analyses could not be completed because too few studies were included.

Moreover, no evidence of publication bias was found. No obvious asymmetry of the funnel plots was observed, except for HPV-18, -31 and -35, with slightly more small studies reporting higher ORs (Additional file 3). The Egger test was borderline or statistically significant only for HPV-6 (P = 0.091), HPV-35 (P = 0.040) and HPV-62 (P = 0.065).

### Sensitivity analyses

When Peto’s method for fixed-effect models was applied rather than DerSimonian and Laird’s random-effects models, the first six HPV genotypes ranked in the exact same order according to their pooled ORs and yielded similar point estimates with narrower CIs, e.g., 0.65 [0.52, 0.80] for HPV-18 and 0.36 [0.26, 0.49] for HPV-45. More generally, the top 12 were common to both methods except for HPV-30 versus HPV-34 (Additional file 4).

Second, using the random-effects model with a lower CC = 0.25, the first 10 genotypes were the same but ranked in a different order starting at the fifth. Point estimates and CIs were virtually unchanged (e.g., 0.63 [0.50, 0.78] for HPV-18 and 0.33 [0.21, 0.53] for HPV-45). With CC = 0, the discrepancy was much greater: HPV-18 ranked second (0.62 [0.50, 0.77]) after HPV-40, which had a very wide CI, and HPV-45 ranked fourth (0.32 [0.20, 0.52]) after HPV-44. Indeed, fewer studies were included in this analysis (those with no control or no case for the HPV genotype considered were excluded). In particular, the OR associated with HPV-67 (ranked 8 in our main analysis) could not be calculated but eight genotypes (HPV-18, -33, -34, -39, -45, -58, -59 and -69) among the first 10 were common to the two methods (Additional file 4).

Finally, considering HPV-negative women as the reference group yielded very different estimates but a not-so-different pattern in terms of ranking. First, HPV-16 (which was not assessed in our relative model) had an OR of 136.7 [70.0, 266.9]. Except for HPV-16, seven of the first 10 genotypes were common to both methods, but with scrambled orders. In particular, HPV-67 (89.8 [13.6, 593.6]) and HPV-69 (81.0 [5.0, 1313.3]) ranked between HPV-18 (99.1 [49.8, 197.2]) and HPV-45 (70.5 [34.3, 144.8]) but their CIs were particularly wide (Additional file 4).

## Discussion

Conducting meta-analyses of published epidemiologic studies enabled us to rank 32 HPV genotypes according to their oncogenic potential relative to HPV-16. All HPV genotypes studied had much smaller estimated oncogenic potentials than HPV-16, reinforcing its being considered the “most potent HPV genotype” [8,11]. Following HPV-16, the HPV genotypes with the highest oncogenic potentials were, in descending order of their pooled ORs: HPV-18, -45, -69, -58, -31, -33, -34, -67, -39, -59, -73 and -52.

Globally, it is reassuring that the HPV genotypes accorded the highest relative oncogenic potentials in our analyses were indeed IARC-classified as carcinogenic. Moreover, the genotypes with the highest ORs and relatively narrow CIs, namely HPV-18, -31, -33, -39,-45, -52, -58 and -59, all belong to the α-7 and α-9 species and are IARC-classified as carcinogenic to humans with sufficient evidence [8,11]. Pertinently, all these genotypes except HPV-18 showed largely overlapping CIs, while HPV-18 (α-7) ranked closest to, but distinct from, HPV-16 (α-9), supporting its being considered separately from the other potentially oncogenic genotypes characterized by a risk continuum without a clear break point (Figure 2).

Surprisingly, HPV-35, -51 and -56, also IARC-classified as carcinogenic and commonly detected in ICC [57], ranked lower in our analysis (OR ≤0.15). The same was observed for HPV-68 (α-7), currently IARC-classified as probably carcinogenic and among “probably high-risk genotypes” [10]. So, based on epidemiologic data alone, our analysis would suggest milder oncogenic potentials for HPV-35, -51, -56 and -68 than inferred from the IARC classification, although we acknowledge that more evidence (e.g., mechanistic) needs to be considered [9].

Notably, our results provide insights into the oncogenic potentials of several genotypes currently IARC-classified as probably oncogenic in humans. Our meta-analytic assessment of the oncogenic potentials of HPV-69 and -82 (both α-5 species), -30 (α-6), -67 (α-9), and -34 and -73 (α-11) was based on small numbers of cases, which yielded particularly wide CIs. However, they ranked among carcinogenic HPV genotypes, which could suggest stronger oncogenic potentials than assumed so far. To date, evidence for HPV-30, -34 and -69 has relied on their phylogenetic analogy to other HPV genotypes, while HPV-67, -73 and -82 were positively associated with cancer but lack strong mechanistic evidence [8,11]. In contrast, HPV-53, -66 and -70, also placed in the probably carcinogenic subgroup [8,11], had lower relative ORs in our analyses. Hence, overall, our analysis of available epidemiologic data provided more discrepant results for the probably carcinogenic genotype distribution.

Conversely, little to no mechanistic evidence supports that HPV-6 and -11 (both α-10 species), which commonly cause benign genital warts, can contribute to carcinogenesis and they remain unclassifiable as to their carcinogenicity in humans [8,11]. Our meta-analyses consistently ranked both at the end of the distribution with estimated pooled ORs ≤0.15. We should mention that our HPV-16 reference model did not allow us to disentangle less oncogenic from non-oncogenic genotypes.

Finally, no epidemiologic evidence suggests cervical oncogenicity for HPV-40 and -44 [11]. In phylogenetic terms, these genotypes belong rather to non-oncogenic species (α-8 and -10, respectively) [58] and have been considered “low-risk” genotypes [10]. In our main analysis, these two genotypes ranked before HPV-6 and -11. However, their estimated ORs were based on limited data and their classification was not robust in the sensitivity analyses (Additional file 4). Taken together, our results do not support HPV-40 and -44 oncogenic potentials.

Strengths of our study derive from methodologic choices. To date, the assessment of the HPV-genotype–specific oncogenic potential in cervical cancer has mainly been based on HPV-genotype–prevalence data among cases [13,57,59,60]. However, that knowledge alone may be insufficient to fully appreciate each genotype’s oncogenic potential. For a given HPV genotype, low frequency in ICC (corresponding to a small etiologic fraction) could reflect low prevalence in the general population or low oncogenic potential. In our study, HPV-genotype ranking according to their prevalences in cases visibly differed from that according to their estimated relative oncogenic potentials. For example, HPV-39 and -59 (both α-7), about four times less prevalent than HPV-52 (α-9), had higher oncogenic potentials estimated by their pooled ORs (Table 2); yet all three genotypes are IARC-classified as carcinogenic.

To our knowledge, the risks associated with the different HPV genotypes have rarely been assessed and HPV-negative, rather than HPV-16–positive, subjects served as the reference group to calculate ORs [4,10] with at least one exception [61]. Our similar third sensitivity analysis found lower OR estimates of the same order of magnitude as those previously published [10], e.g., respectively, 136.7 versus 281.9 for HPV-16 and 99.1 versus 222.5 for HPV-18. Notably, that third analysis showed no clear break point between HPV-18 and the other genotypes, with ORs decreasing progressively from HPV-16 to the end, unlike our main analysis. The choice of this reference group may be questioned because it takes uninfected cases into account for OR calculation, even though it is currently accepted that persistent HPV infection is required to cause ICC [2-4]. With few or no HPV-negative cases of cervical cancer expected, estimation of ORs and their CIs may become problematic. Therefore, we chose the unusual approach of using HPV-16–infected subjects as the reference category. HPV-16’s high oncogenic potential is well-documented [8,11], this genotype is highly prevalent in cases [13,57] and often identified in women with normal cytology [62,63]. In our opinion, considering HPV-16–infected women as the reference group seemed more consistent with the natural history of cervical cancer and could be more appropriate for estimating HPV-genotype oncogenic potentials, regardless of their prevalence. However, the control group’s baseline risk of developing ICC cannot be considered low, meaning that ORs cannot be directly interpreted as an accurate estimate of the relative risk, even though they can be used to rank genotypes. Alternatively, estimating ORs relative to an established low-risk genotype, e.g., HPV-6, was limited by the small, if not inexistent, numbers of ICC cases positive for such a genotype.

Herein, we combined study ORs using the random-effects model, as sometimes recommended to perform meta-analyses of published data [64]. This approach implies wider CIs than in a fixed-effect model because, in addition to random fluctuations, the random-effects model allows for variability of the real risk. However, sensitivity analyses showed our results to be consistent with those obtained using Peto’s method (Additional file 4), thereby indicating that the wide CIs mostly reflected the scarcity of epidemiologic data, rather than the choice of statistical models.

Some authors questioned the use of CC in the random-effects model, when the underlying risk varies among studies [65]. Our sensitivity analyses with a halved CC factor differed only slightly from our main results. In contrast, applying no CC raised estimation difficulties preventing the calculation of two pooled ORs (Additional file 4). Nevertheless, our choice is supported by the consistencies, both external (with the literature) and internal (across other sensitivity analyses), of our findings after correction.

Our meta-analysis has several limitations that warrant being mentioned. First, we applied stringent selection criteria, including only studies with sufficient numbers of HPV-positive cases and controls. That choice rendered the several large investigations conducted in North America ineligible [66,67], which is consistent with 85% of ICC cases occurring in developing countries [1], and HPV-vaccine trials being conducted more frequently in Asia-Pacific, Europe or Latin America than North America [68,69]. Nevertheless, although the distributions of HPV genotypes vary across populations [18,57,59,63], no evidence indicates that HPV-type–specific oncogenic potential could differ according to geographic area. Moreover, the continent did not explain heterogeneity in our meta-analyses.

Second, basing this study on summary data meant we could not control for age, despite its being a critical variable, closely associated with HPV infection, clearance, persistence and progression. Age information was frequently missing and rarely available for HPV-positive cases and controls specifically. Controls tended to be 10 years younger than cases on average, possibly reflecting different stages in the natural history of cervical cancer. The peak prevalence of cervical HPV infection coincides closely with first-time sexual intercourse, at around 20 years of age, while that of ICC occurs at 40–50 years [70]. It was reassuring that the comparability of age distributions between cases and controls did not clearly explain heterogeneity in our meta-analyses.

Third, the small number of cases infected with some HPV genotypes hindered precise estimations of their oncogenic potentials. This paucity is partly due to our strict definition of cases as having ICC. This choice was motivated by the natural history of cervical cancer, according to which precancerous lesions, even high-grade cervical intraepithelial neoplasia, may regress in a substantial proportion of cases [71]. Previous studies [17,72] might have been more permissive, assimilating high-grade lesions and ICC cases, especially longitudinal studies, often limited by the low numbers of ICC during the follow-up. Moreover, clinical management guidelines also recommend the excision of precancerous lesions, and will continue to do so as long as whether these would regress or progress cannot be foreseen [73].

Fourth, we did not distinguish between ICC histologic types, even though HPV-18 could be more prevalent in adenocarcinomas than squamous cell carcinomas [13]. However, the HPV-genotype–specific distribution according to histologic type was seldom reported in selected studies. When histologic type was reported, most were squamous cell carcinomas, which is the most common histologic cervical cancer type [74].

Fifth, our analysis was limited by the variety of sample types and HPV assays, as in previously reported meta-analyses of HPV-genotype–specific prevalences [57,59,60]. Although all HPV-detection methods were PCR-based, sensitivity and specificity of PCR protocols varied across studies and numerous HPV genotypes were not detected by some of them. However, each study used the same HPV-typing method for cases and controls, so it is unlikely that the differences among studies affected our estimates. Moreover, the heterogeneity in our meta-analyses was not explained by the HPV-detection threshold for cases.

Finally, because the components of multiple infections were seldom available, the oncogenic potential of each HPV genotype was assessed without distinguishing between single or coinfection. Thus, the oncogenic potentials of some HPV genotypes might have been overestimated in our meta-analysis if they had been coinfection partners with established high-risk genotypes, e.g., HPV-16 or -18, and were wrongly accorded equal weight in cancerous lesions even though the high-risk genotype was solely responsible for the lesions [11]. That possibility could explain HPV-11’s unexpectedly higher oncogenic potential. Moreover, for studies that did not report coinfection, misattribution of the causal HPV genotype could bias the estimated oncogenic potentials of coinfecting HPV genotypes either way [75]. A new generation of molecular studies involving lesion microdissection and HPV-E6/E7 expression could provide valuable information to assess more specifically each HPV genotype’s oncogenic potential [9,76].

## Conclusions

Our results provide further evidence reinforcing the high oncogenic potentials of genotypes HPV-18, -31, -33, -45, -52 and -58, already classified as high-risk for ICC. They also highlight the need to include in detection kits HPV-34, -67, -69 and -73, for which epidemiologic data are currently lacking, and to further examine their possibly underestimated oncogenic potentials. Moreover, although HPV-39 and -59 belong to the same α-7 species as HPV-18, they are not, at present, included in a future nonavalent anti-HPV vaccine (HPV-6, -11, -16, -18, -31, -33, -45, -52 and -58) [77]. Those genotypes may deserve further consideration, owing to accumulating evidence (relatively precise estimates) and their classification among the 10 most oncogenic genotypes after HPV-16 in our meta-analyses. Pooling individual data from presently available and future studies investigating these genotypes would allow more robust estimates, especially if controlled for age. Overall, such findings may have important implications for the prevention of cervical cancer and could help guide HPV-based–screening programs [78] and the composition of the second-generation anti-HPV vaccines [79].

## Abbreviations

CC: Continuity correction; CI: Confidence interval; HPV: Human papillomavirus; IARC: International Agency for Research on Cancer; ICC: Invasive cervical cancer; OR: Odds ratio; PCR: Polymerase chain reaction.

## Competing interests

The authors declare that they have no competing interest.

## Authors’ contributions

EB conducted the literature review, study analyses and wrote the first draft of the manuscript. ACMT and MPS supervised the study and participated in the literature review, analysis and writing. MF, IH, EDA and DG critically revised the manuscript and contributed to its Introduction and Discussion. ACMT and DG designed the study’s analytic strategy. All authors read and approved the final manuscript.

## Pre-publication history

The pre-publication history for this paper can be accessed here:

http://www.biomedcentral.com/1471-2334/13/373/prepub

## Supplementary Material

Additional file 1 Search strategies. Click here for file

Additional file 2 **Meta-analyses assessing the relative oncogenic potential of each human papillomavirus (HPV) genotype (forest plots).** Studies are listed in alphabetical order. Each study is represented by a black cross, which corresponds to the odds ratio (OR) point estimate; a grey square, whose area reflects the weight each study contributes in the meta-analysis; and a horizontal line, which spans the 95% confidence interval (CI). The diamond at the bottom of the graph represents the combined OR and its 95% CI. The solid vertical line is an oncogenic potential equal to that of HPV-16 (OR 1.0) and the dotted vertical line indicates the value of the combined ORs from the random-effects model. The graphs were generated by Stata command metan (adapted from [26] pp 14 and 33). Click here for file

Additional file 3 **Bias assessment for each meta-analysis (funnel plots).** Each dot represents one study. The solid vertical line is the pooled odds ratio (OR). Diagonal dashed lines represent the pseudo 95% confidence limits around the pooled OR for each standard error of the ordinate vertical axis values, defining a funnel within which 95% of the studies should lie in the absence of heterogeneity or selection biases. The yellow line is the fitted linear-regression line of the OR plotted against its standard error (both on natural logarithm scales) and corresponds to Egger’s test for funnel-plot asymmetry. The graphs were generated by the Stata command metafunnel (adapted from [29] pp 113 and 115). Click here for file

Additional file 4 **Sensitivity analyses of human papillomavirus genotype ranking.** *Analyses using a fixed-effect model [27]. †Analyses using DerSimonian and Laird’s random-effects model [20] with a continuity correction (CC) = 0.25. ‡Analyses using DerSimonian and Laird’s random-effects model [20] with CC = 0. HPV-62 and -69 ORs could not be calculated. §Analyses using DerSimonian and Laird’s random-effects model [20] (CC = 0.5), with HPV-negative subjects as the reference group. In this model, unlike the preceding ones, the pooled OR for HPV-16 could be estimated. Abbreviations: HPV, human papilloma virus; OR, odds ratio; CI, confidence interval. Click here for file
