# Supplementary material for: Comparing human papillomavirus prevalences in women with normal cytology or invasive cervical cancer to rank genotypes according to their oncogenic potential: a meta-analysis of observational studies
Source: BMC Infect Dis. 2013 Aug 13;13:373. doi: 10.1186/1471-2334-13-373 (PMC3751808; doi:10.1186/1471-2334-13-373)
Supplement: Additional file 1 — Search strategies. [file 1471-2334-13-373-S1.doc]

**Additional file 1:** Search strategies

| **Medline (PubMed)** |
| --- |
| #1 "Female" [Mesh]  #2 "Humans" [Mesh]  #3 "Papillomavirus Infections" [Mesh]  #4 #1 AND #2 AND #3  #5 "DNA Probes, HPV" [Mesh]  #6 "DNA, Viral" [Mesh]  #7 "Genotype" [Mesh]  #8 "Polymerase Chain Reaction" [Mesh]  #9 "Sequence Analysis, DNA" [Mesh]  #10 #5 OR #6 OR #7 OR #8 OR #9  #11 "Uterine Cervical Neoplasms" [Mesh]  #12 "Cervix Uteri" [Mesh]  #13 #11 OR #12  #14 "Epidemiologic Studies" [Mesh]  #15 "Prevalence" [Mesh]  #16 "Incidence" [Mesh]  #17 #14 OR #15 OR #16  #18 #4 AND #10 AND #13 AND #17  #19 English [lang]  #20 French [lang]  #21 German [lang]  #22 Spanish [lang]  #23 #19 OR #20 OR #21 OR #22  #24 "1995" [PDAT]: "2011" [PDAT]  #25 #18 AND #23 AND #24  #26 "Review" [Publication Type]  #27 "Comment" [Publication Type]  #28 "Meta-Analysis" [Publication Type]  #29 "Editorial" [Publication Type]  #30 "Letter" [Publication Type]  #31 #26 OR #27 OR #28 OR #29 OR #30  #32 #25 NOT #31 |
| **Embase** |
| 'papillomavirus'/exp  AND ('dna probe'/exp OR 'virus dna'/exp OR 'genotype'/exp OR 'polymerase chain reaction'/exp OR 'dna sequence'/exp)  AND ('uterine cervix'/exp OR 'uterine cervix carcinoma'/exp)  AND 'epidemiology'/exp  AND ([german]/lim OR [english]/lim OR [french]/lim OR [spanish]/lim)  AND [embase]/lim  AND [1995-2011]/py |
