# Supplementary material for: Comparing human papillomavirus prevalences in women with normal cytology or invasive cervical cancer to rank genotypes according to their oncogenic potential: a meta-analysis of observational studies
Source: BMC Infect Dis. 2013 Aug 13;13:373. doi: 10.1186/1471-2334-13-373 (PMC3751808; doi:10.1186/1471-2334-13-373)
Supplement: Additional file 2 — Meta-analyses assessing the relative oncogenic potential of each human papillomavirus (HPV) genotype (forest plots). Studies are listed in alphabetical order. Each study is represented by a black cross, which corresponds to the odds ratio (OR) point estimate; a grey square, whose area reflects the weight each study contributes in the meta-analysis; and a horizontal line, which spans the 95% confidence interval (CI). The diamond at the bottom of the graph represents the combined OR and its 95% CI. The solid vertical line is an oncogenic potential equal to that of HPV-16 (OR 1.0) and the dotted vertical line indicates the value of the combined ORs from the random-effects model. The graphs were generated by Stata command metan (adapted from [26] pp 14 and 33). [file 1471-2334-13-373-S2.pdf]

Additional File 2. Meta-analyses assessing the relative oncogenic potential of each human papillomavirus (HPV) genotype (forest plots). Studies are listed in alphabetical order. Each study is represented by a black cross, which corresponds to the odds ratio (OR) point estimate; a grey square, whose area reflects the weight each study contributes in the meta-analysis; and a horizontal line, which spans the 95% confidence interval (CI). The diamond at the bottom of the graph represents the combined OR and its 95% CI. The solid vertical line is an oncogenic potential equal to that of HPV-16 (OR 1.0) and the dotted vertical line indicates the value of the combined ORs from the random-effects model. The graphs were generated by Stata command metan.

(a)

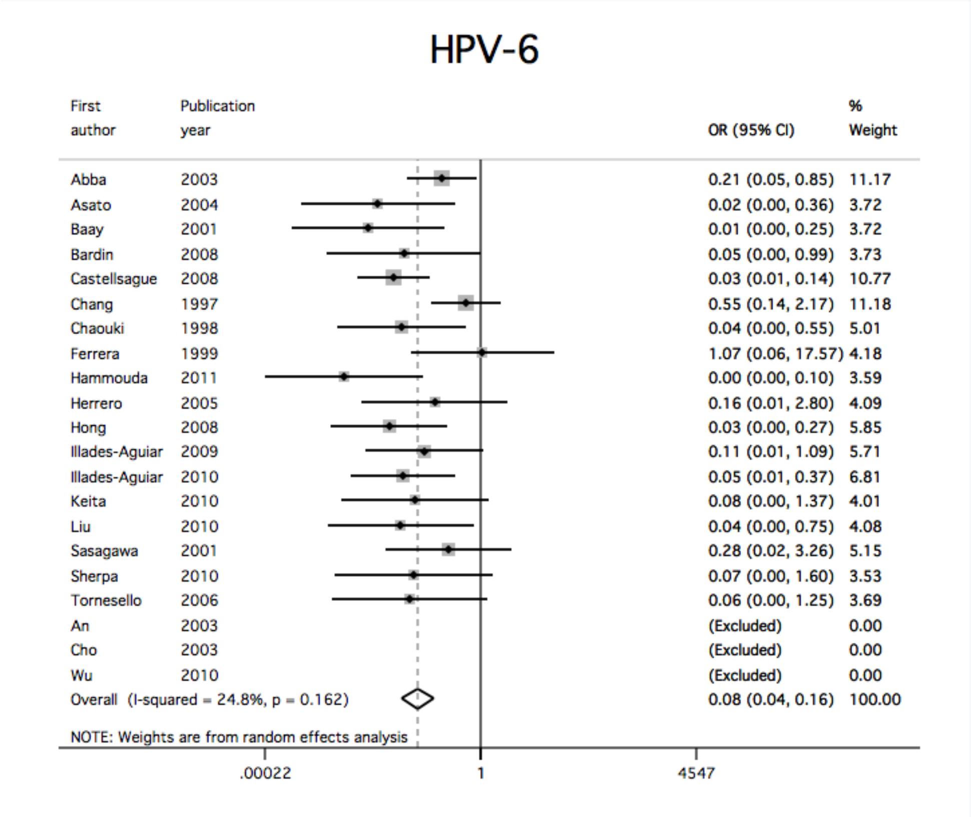

(b)

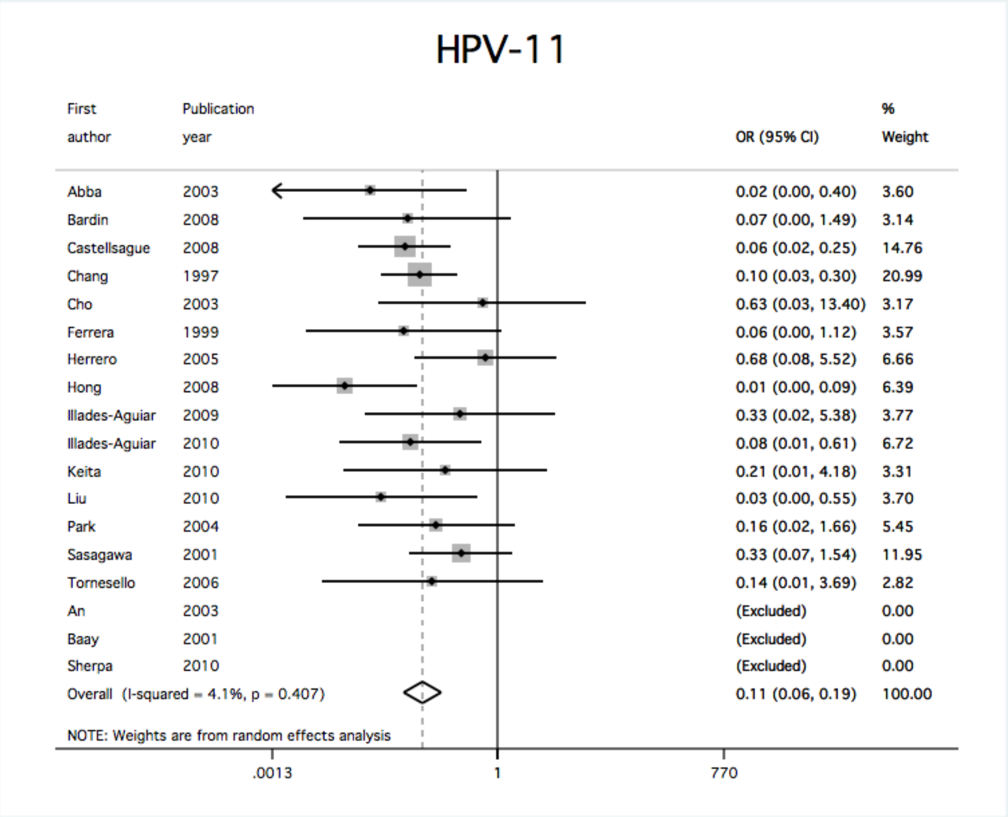
HPV-11
First author
Publication year
OR (95% CI)
% Weight
Abba 2003 0.02 (0.00, 0.40) 3.60
Bardin 2008 0.07 (0.00, 1.49) 3.14
Castellsague 2008 0.06 (0.02, 0.25) 14.76
Chang 1997 0.10 (0.03, 0.30) 20.99
Cho 2003 0.63 (0.03, 13.40) 3.17
Ferrera 1999 0.06 (0.00, 1.12) 3.57
Herrero 2005 0.68 (0.08, 5.52) 6.66
Hong 2008 0.01 (0.00, 0.09) 6.39
Illades-Aguiar 2009 0.33 (0.02, 5.38) 3.77
Illades-Aguiar 2010 0.08 (0.01, 0.61) 6.72
Keita 2010 0.21 (0.01, 4.18) 3.31
Liu 2010 0.03 (0.00, 0.55) 3.70
Park 2004 0.16 (0.02, 1.66) 5.45
Sasagawa 2001 0.33 (0.07, 1.54) 11.95
Tornesello 2006 0.14 (0.01, 3.69) 2.82
An 2003 (Excluded) 0.00
Baay 2001 (Excluded) 0.00
Sherpa 2010 (Excluded) 0.00
Overall (I-squared = 4.1%, p = 0.407) 0.11 (0.06, 0.19) 100.00
NOTE: Weights are from random effects analysis
.0013
1
770

(c)

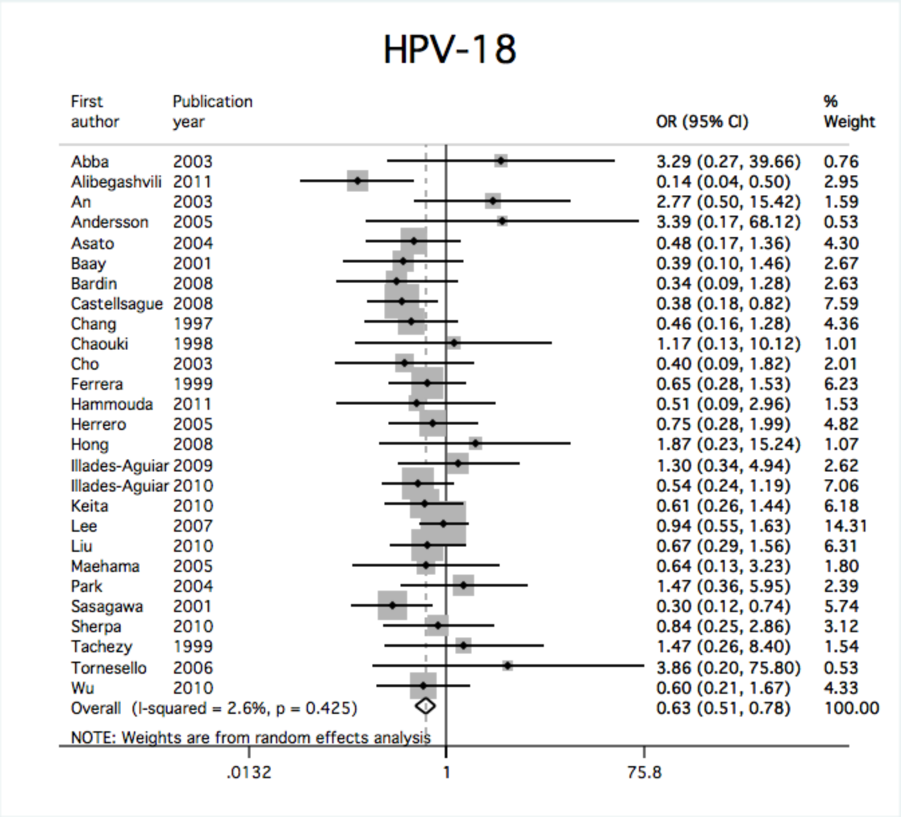
HPV-18
First author
Publication year
OR (95% CI)
% Weight
Abba 2003 3.29 (0.27, 39.66) 0.76
Alibegashvili 2011 0.14 (0.04, 0.50) 2.95
An 2003 2.77 (0.50, 15.42) 1.59
Andersson 2005 3.39 (0.17, 68.12) 0.53
Asato 2004 0.48 (0.17, 1.36) 4.30
Baay 2001 0.39 (0.10, 1.46) 2.67
Bardin 2008 0.34 (0.09, 1.28) 2.63
Castellsague 2008 0.38 (0.18, 0.82) 7.59
Chang 1997 0.46 (0.16, 1.28) 4.36
Chaouki 1998 1.17 (0.13, 10.12) 1.01
Cho 2003 0.40 (0.09, 1.82) 2.01
Ferrera 1999 0.65 (0.28, 1.53) 6.23
Hammouda 2011 0.51 (0.09, 2.96) 1.53
Herrero 2005 0.75 (0.28, 1.99) 4.82
Hong 2008 1.87 (0.23, 15.24) 1.07
Illades-Aguiar 2009 1.30 (0.34, 4.94) 2.62
Illades-Aguiar 2010 0.54 (0.24, 1.19) 7.06
Keita 2010 0.61 (0.26, 1.44) 6.18
Lee 2007 0.94 (0.55, 1.63) 14.31
Liu 2010 0.67 (0.29, 1.56) 6.31
Maehama 2005 0.64 (0.13, 3.23) 1.80
Park 2004 1.47 (0.36, 5.95) 2.39
Sasagawa 2001 0.30 (0.12, 0.74) 5.74
Sherpa 2010 0.84 (0.25, 2.86) 3.12
Tachezy 1999 1.47 (0.26, 8.40) 1.54
Tornesello 2006 3.86 (0.20, 75.80) 0.53
Wu 2010 0.60 (0.21, 1.67) 4.33
Overall (I-squared = 2.6%, p = 0.425) 0.63 (0.51, 0.78) 100.00
NOTE: Weights are from random effects analysis
.0132
1
75.8

(d)

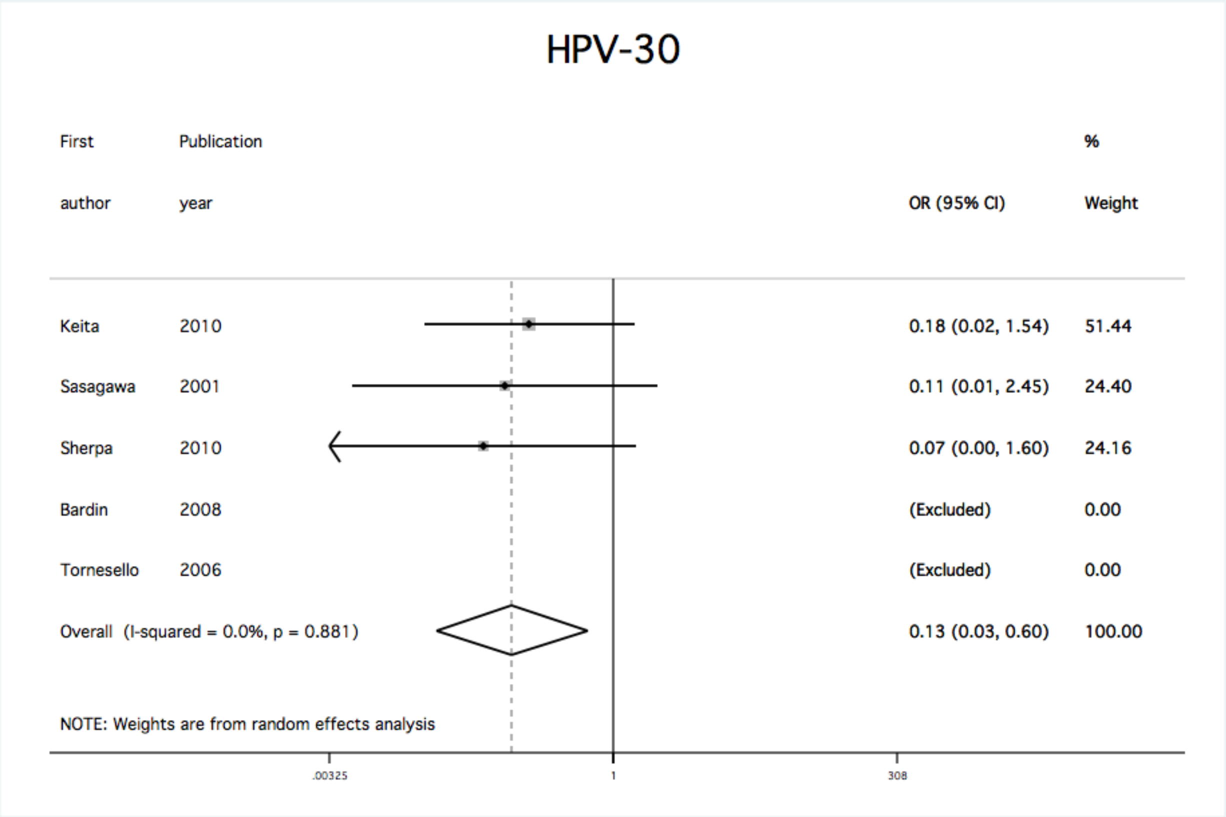

HPV-30
First author
Publication year
OR (95% CI)
% Weight
Keita 2010 0.18 (0.02, 1.54) 51.44
Sasagawa 2001 0.11 (0.01, 2.45) 24.40
Sherpa 2010 0.07 (0.00, 1.60) 24.16
Bardin 2008 (Excluded) 0.00
Tornesello 2006 (Excluded) 0.00
Overall (I-squared = 0.0%, p = 0.881) 0.13 (0.03, 0.60) 100.00
NOTE: Weights are from random effects analysis
.00325
1
308

(e)

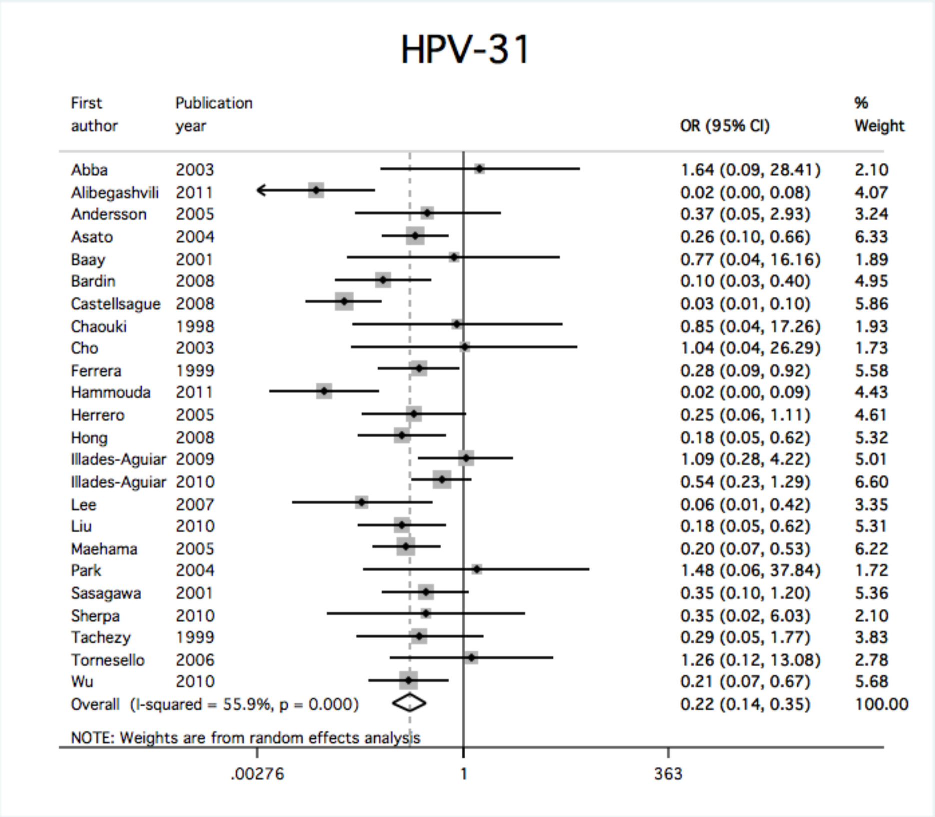

HPV-31
First author
Publication year
OR (95% CI)
% Weight
Abba 2003 1.64 (0.09, 28.41) 2.10
Alibegashvili 2011 0.02 (0.00, 0.08) 4.07
Andersson 2005 0.37 (0.05, 2.93) 3.24
Asato 2004 0.26 (0.10, 0.66) 6.33
Baay 2001 0.77 (0.04, 16.16) 1.89
Bardin 2008 0.10 (0.03, 0.40) 4.95
Castellsague 2008 0.03 (0.01, 0.10) 5.86
Chaouki 1998 0.85 (0.04, 17.26) 1.93
Cho 2003 1.04 (0.04, 26.29) 1.73
Ferrera 1999 0.28 (0.09, 0.92) 5.58
Hammouda 2011 0.02 (0.00, 0.09) 4.43
Herrero 2005 0.25 (0.06, 1.11) 4.61
Hong 2008 0.18 (0.05, 0.62) 5.32
Illades-Aguiar 2009 1.09 (0.28, 4.22) 5.01
Illades-Aguiar 2010 0.54 (0.23, 1.29) 6.60
Lee 2007 0.06 (0.01, 0.42) 3.35
Liu 2010 0.18 (0.05, 0.62) 5.31
Maehama 2005 0.20 (0.07, 0.53) 6.22
Park 2004 1.48 (0.06, 37.84) 1.72
Sasagawa 2001 0.35 (0.10, 1.20) 5.36
Sherpa 2010 0.35 (0.02, 6.03) 2.10
Tachezy 1999 0.29 (0.05, 1.77) 3.83
Tornesello 2006 1.26 (0.12, 13.08) 2.78
Wu 2010 0.21 (0.07, 0.67) 5.68
Overall (I-squared = 55.9%, p = 0.000) 0.22 (0.14, 0.35) 100.00
NOTE: Weights are from random effects analysis
.00276
1
363

(f)

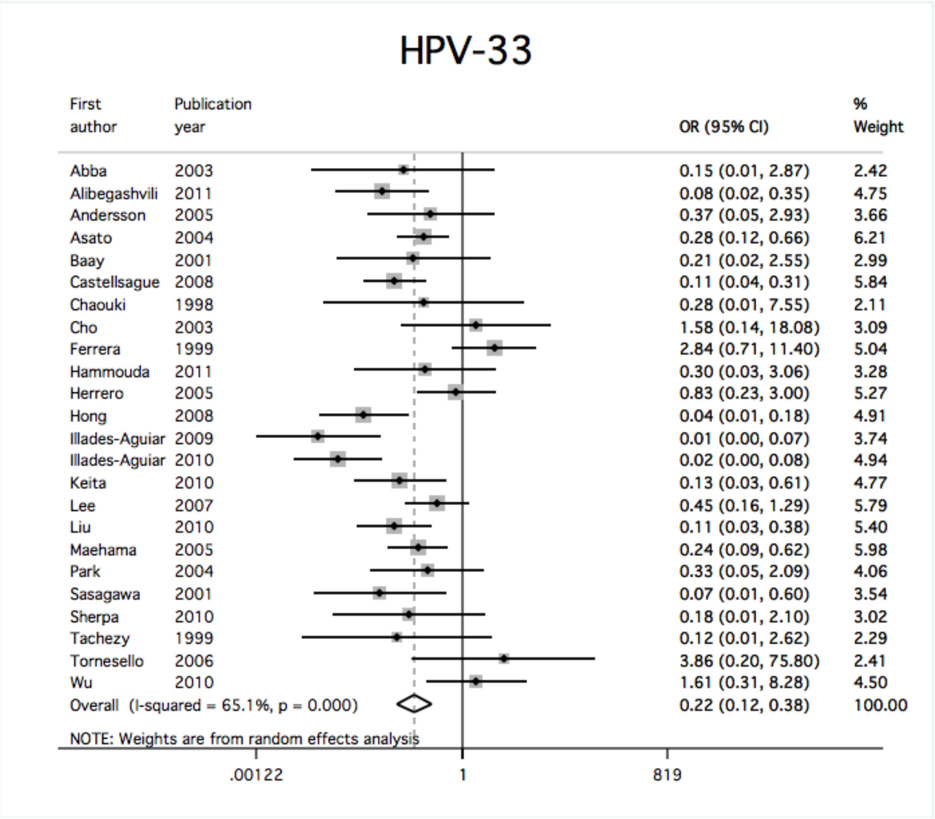
HPV-33
First author
Publication year
OR (95% CI)
% Weight
Abba 2003 0.15 (0.01, 2.87) 2.42
Alibegashvili 2011 0.08 (0.02, 0.35) 4.75
Andersson 2005 0.37 (0.05, 2.93) 3.66
Asato 2004 0.28 (0.12, 0.66) 6.21
Baay 2001 0.21 (0.02, 2.55) 2.99
Castellsague 2008 0.11 (0.04, 0.31) 5.84
Chaouki 1998 0.28 (0.01, 7.55) 2.11
Cho 2003 1.58 (0.14, 18.08) 3.09
Ferrera 1999 2.84 (0.71, 11.40) 5.04
Hammouda 2011 0.30 (0.03, 3.06) 3.28
Herrero 2005 0.83 (0.23, 3.00) 5.27
Hong 2008 0.04 (0.01, 0.18) 4.91
Illades-Aguiar 2009 0.01 (0.00, 0.07) 3.74
Illades-Aguiar 2010 0.02 (0.00, 0.08) 4.94
Keita 2010 0.13 (0.03, 0.61) 4.77
Lee 2007 0.45 (0.16, 1.29) 5.79
Liu 2010 0.11 (0.03, 0.38) 5.40
Maehama 2005 0.24 (0.09, 0.62) 5.98
Park 2004 0.33 (0.05, 2.09) 4.06
Sasagawa 2001 0.07 (0.01, 0.60) 3.54
Sherpa 2010 0.18 (0.01, 2.10) 3.02
Tachezy 1999 0.12 (0.01, 2.62) 2.29
Tornesello 2006 3.86 (0.20, 75.80) 2.41
Wu 2010 1.61 (0.31, 8.28) 4.50
Overall (I-squared = 65.1%, p = 0.000) 0.22 (0.12, 0.38) 100.00
NOTE: Weights are from random effects analysis
.00122
1
819

(g)

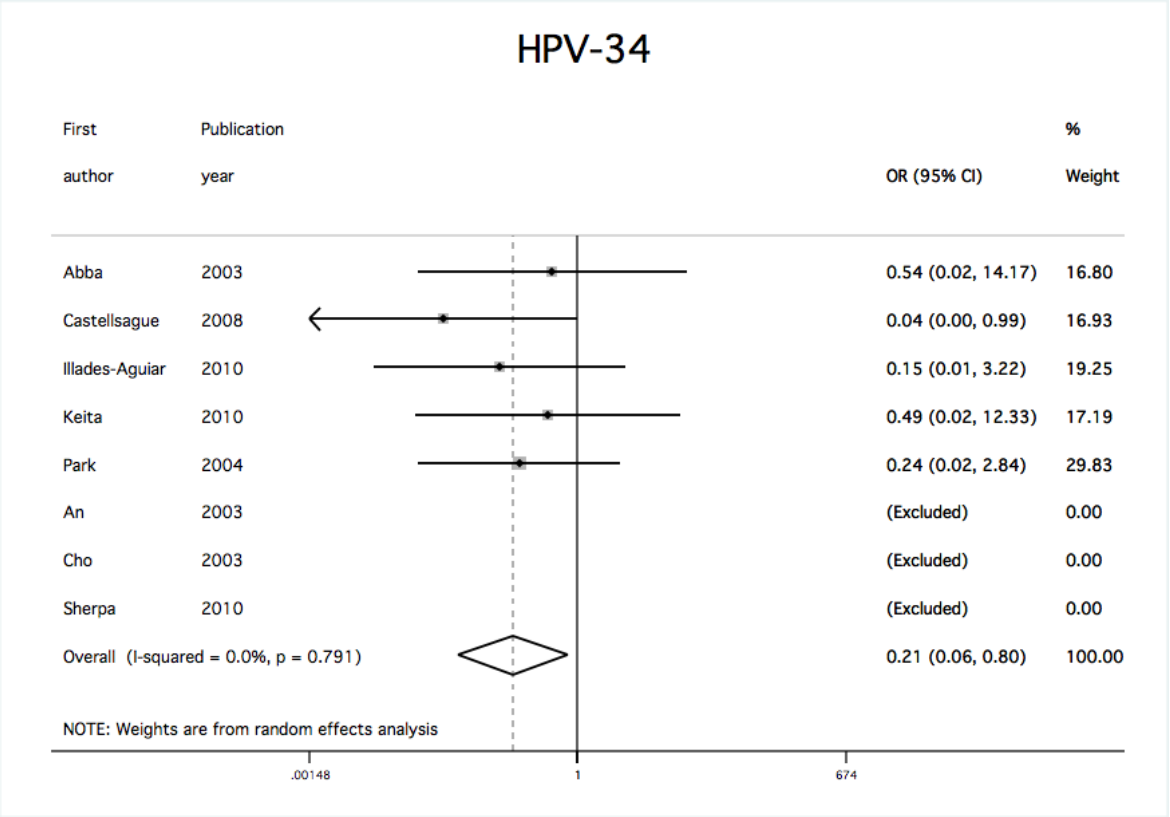
HPV-34
First author
Publication year
OR (95% CI)
% Weight
Abba 2003 0.54 (0.02, 14.17) 16.80
Castellsague 2008 0.04 (0.00, 0.99) 16.93
Illades-Aguiar 2010 0.15 (0.01, 3.22) 19.25
Keita 2010 0.49 (0.02, 12.33) 17.19
Park 2004 0.24 (0.02, 2.84) 29.83
An 2003 (Excluded) 0.00
Cho 2003 (Excluded) 0.00
Sherpa 2010 (Excluded) 0.00
Overall (I-squared = 0.0%, p = 0.791) 0.21 (0.06, 0.80) 100.00
NOTE: Weights are from random effects analysis
.00148
1
674

(h)

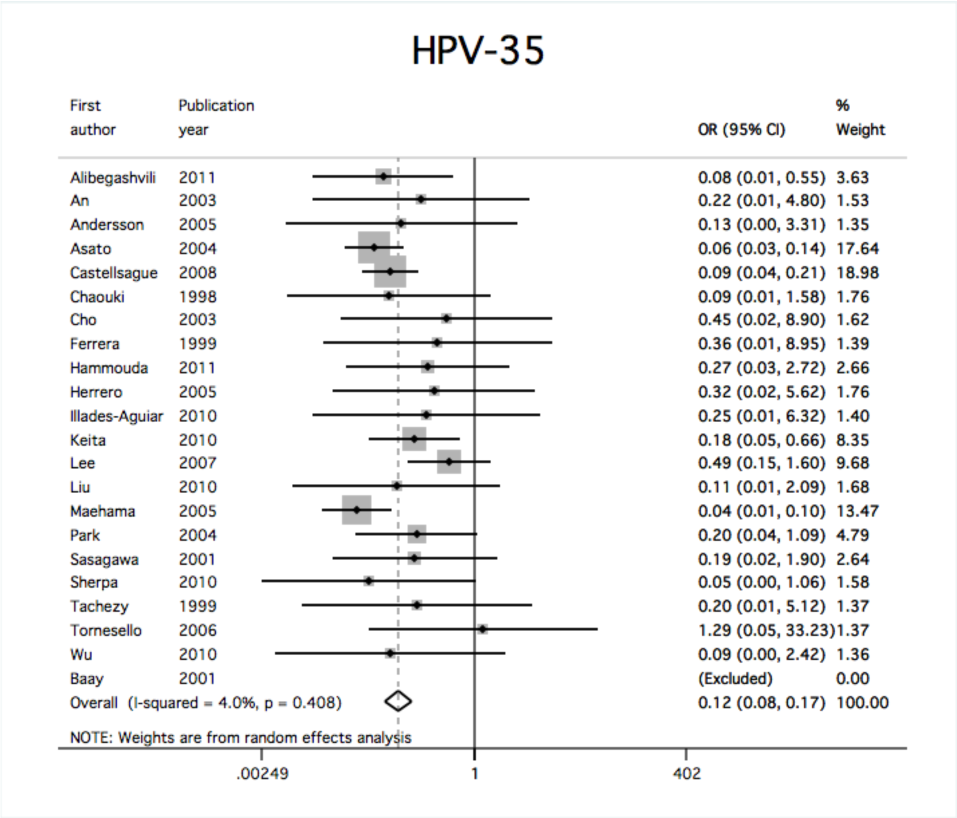
HPV-35
First author
Publication year
OR (95% CI)
% Weight
Alibegashvili 2011 0.08 (0.01, 0.55) 3.63
An 2003 0.22 (0.01, 4.80) 1.53
Andersson 2005 0.13 (0.00, 3.31) 1.35
Asato 2004 0.06 (0.03, 0.14) 17.64
Castellsague 2008 0.09 (0.04, 0.21) 18.98
Chaouki 1998 0.09 (0.01, 1.58) 1.76
Cho 2003 0.45 (0.02, 8.90) 1.62
Ferrera 1999 0.36 (0.01, 8.95) 1.39
Hammouda 2011 0.27 (0.03, 2.72) 2.66
Herrero 2005 0.32 (0.02, 5.62) 1.76
Illades-Aguiar 2010 0.25 (0.01, 6.32) 1.40
Keita 2010 0.18 (0.05, 0.66) 8.35
Lee 2007 0.49 (0.15, 1.60) 9.68
Liu 2010 0.11 (0.01, 2.09) 1.68
Maehama 2005 0.04 (0.01, 0.10) 13.47
Park 2004 0.20 (0.04, 1.09) 4.79
Sasagawa 2001 0.19 (0.02, 1.90) 2.64
Sherpa 2010 0.05 (0.00, 1.06) 1.58
Tachezy 1999 0.20 (0.01, 5.12) 1.37
Tornesello 2006 1.29 (0.05, 33.23) 1.37
Wu 2010 0.09 (0.00, 2.42) 1.36
Baay 2001 (Excluded) 0.00
Overall (I-squared = 4.0%, p = 0.408) 0.12 (0.08, 0.17) 100.00
NOTE: Weights are from random effects analysis
.00249
1
402

(i)

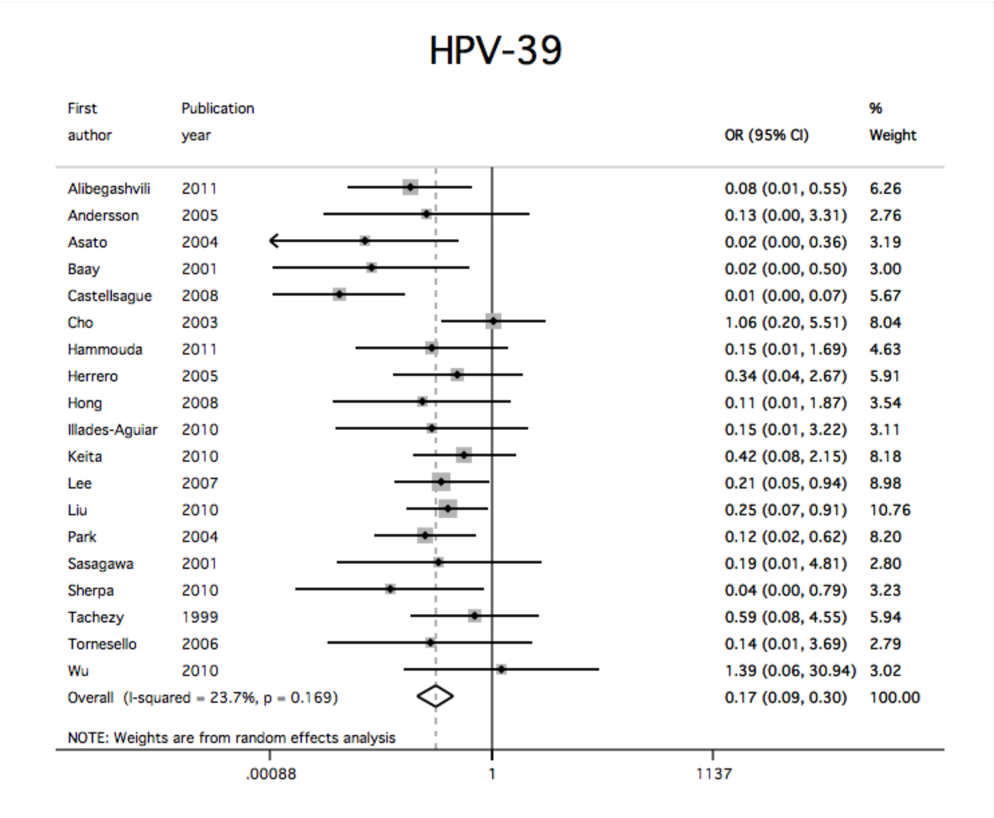
HPV-39
First author
Publication year
OR (95% CI)
% Weight
Alibegashvili 2011 0.08 (0.01, 0.55) 6.26
Andersson 2005 0.13 (0.00, 3.31) 2.76
Asato 2004 0.02 (0.00, 0.36) 3.19
Baay 2001 0.02 (0.00, 0.50) 3.00
Castellsague 2008 0.01 (0.00, 0.07) 5.67
Cho 2003 1.06 (0.20, 5.51) 8.04
Hammouda 2011 0.15 (0.01, 1.69) 4.63
Herrero 2005 0.34 (0.04, 2.67) 5.91
Hong 2008 0.11 (0.01, 1.87) 3.54
Illades-Aguiar 2010 0.15 (0.01, 3.22) 3.11
Keita 2010 0.42 (0.08, 2.15) 8.18
Lee 2007 0.21 (0.05, 0.94) 8.98
Liu 2010 0.25 (0.07, 0.91) 10.76
Park 2004 0.12 (0.02, 0.62) 8.20
Sasagawa 2001 0.19 (0.01, 4.81) 2.80
Sherpa 2010 0.04 (0.00, 0.79) 3.23
Tachezy 1999 0.59 (0.08, 4.55) 5.94
Tornesello 2006 0.14 (0.01, 3.69) 2.79
Wu 2010 1.39 (0.06, 30.94) 3.02
Overall (I-squared = 23.7%, p = 0.169) 0.17 (0.09, 0.30) 100.00
NOTE: Weights are from random effects analysis
.00088
1
1137

(j)

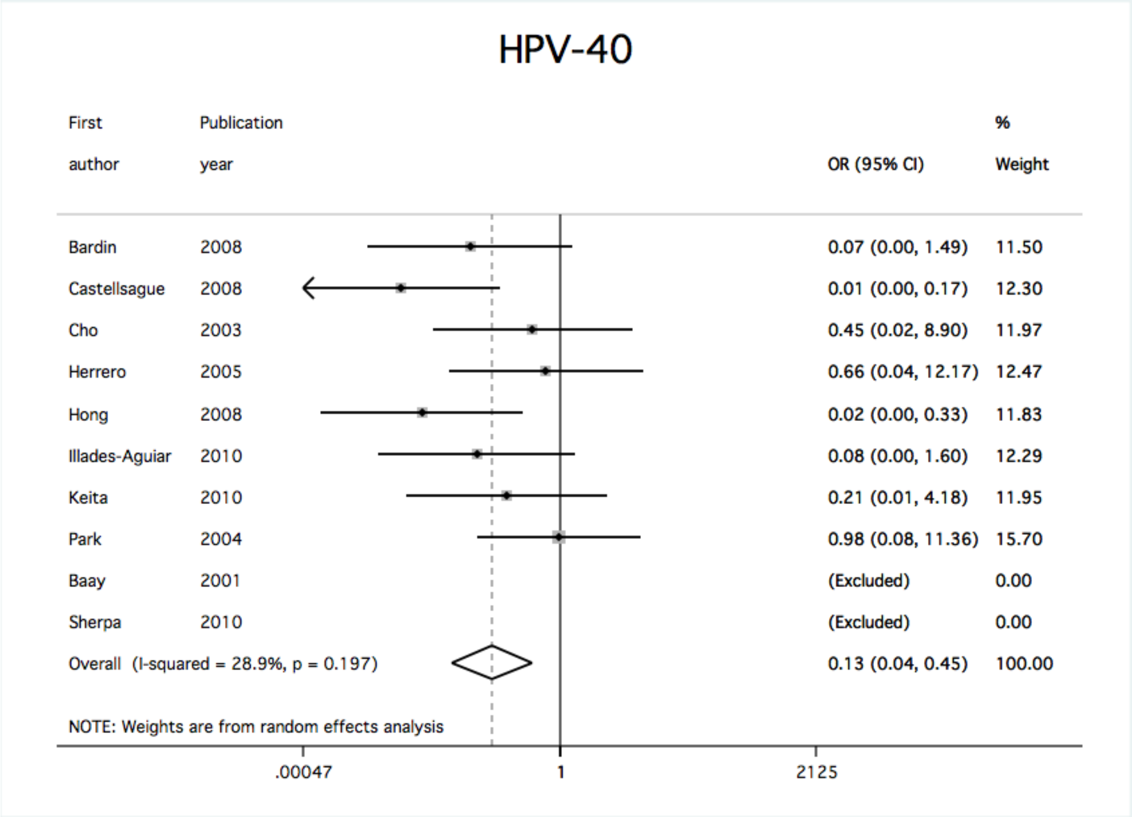
HPV-40
First author
Publication year
OR (95% CI)
% Weight
Bardin 2008 0.07 (0.00, 1.49) 11.50
Castellsague 2008 0.01 (0.00, 0.17) 12.30
Cho 2003 0.45 (0.02, 8.90) 11.97
Herrero 2005 0.66 (0.04, 12.17) 12.47
Hong 2008 0.02 (0.00, 0.33) 11.83
Illades-Aguiar 2010 0.08 (0.00, 1.60) 12.29
Keita 2010 0.21 (0.01, 4.18) 11.95
Park 2004 0.98 (0.08, 11.36) 15.70
Baay 2001 (Excluded) 0.00
Sherpa 2010 (Excluded) 0.00
Overall (I-squared = 28.9%, p = 0.197) 0.13 (0.04, 0.45) 100.00
NOTE: Weights are from random effects analysis
.00047
1
2125

(k)

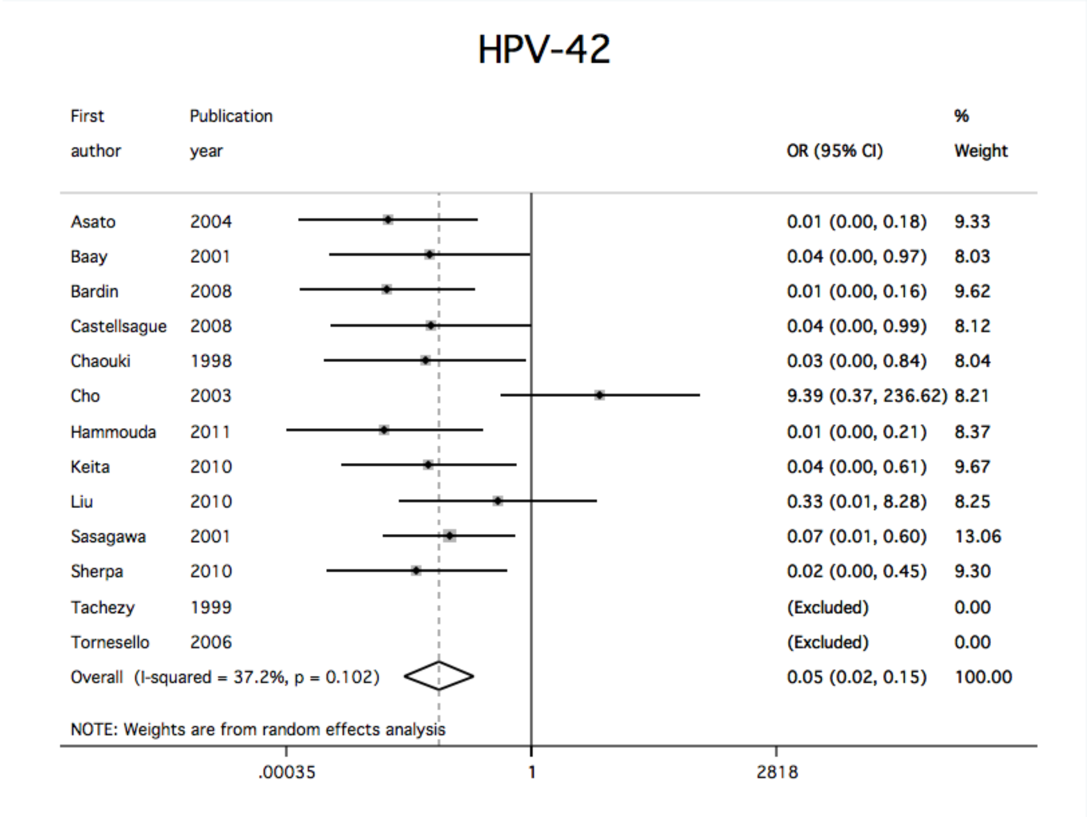
HPV-42
First author
Publication year
OR (95% CI)
% Weight
Asato 2004 0.01 (0.00, 0.18) 9.33
Baay 2001 0.04 (0.00, 0.97) 8.03
Bardin 2008 0.01 (0.00, 0.16) 9.62
Castellsague 2008 0.04 (0.00, 0.99) 8.12
Chaouki 1998 0.03 (0.00, 0.84) 8.04
Cho 2003 9.39 (0.37, 236.62) 8.21
Hammouda 2011 0.01 (0.00, 0.21) 8.37
Keita 2010 0.04 (0.00, 0.61) 9.67
Liu 2010 0.33 (0.01, 8.28) 8.25
Sasagawa 2001 0.07 (0.01, 0.60) 13.06
Sherpa 2010 0.02 (0.00, 0.45) 9.30
Tachezy 1999 (Excluded) 0.00
Tornesello 2006 (Excluded) 0.00
Overall (I-squared = 37.2%, p = 0.102) 0.05 (0.02, 0.15) 100.00
NOTE: Weights are from random effects analysis
.00035
1
2818

(l)

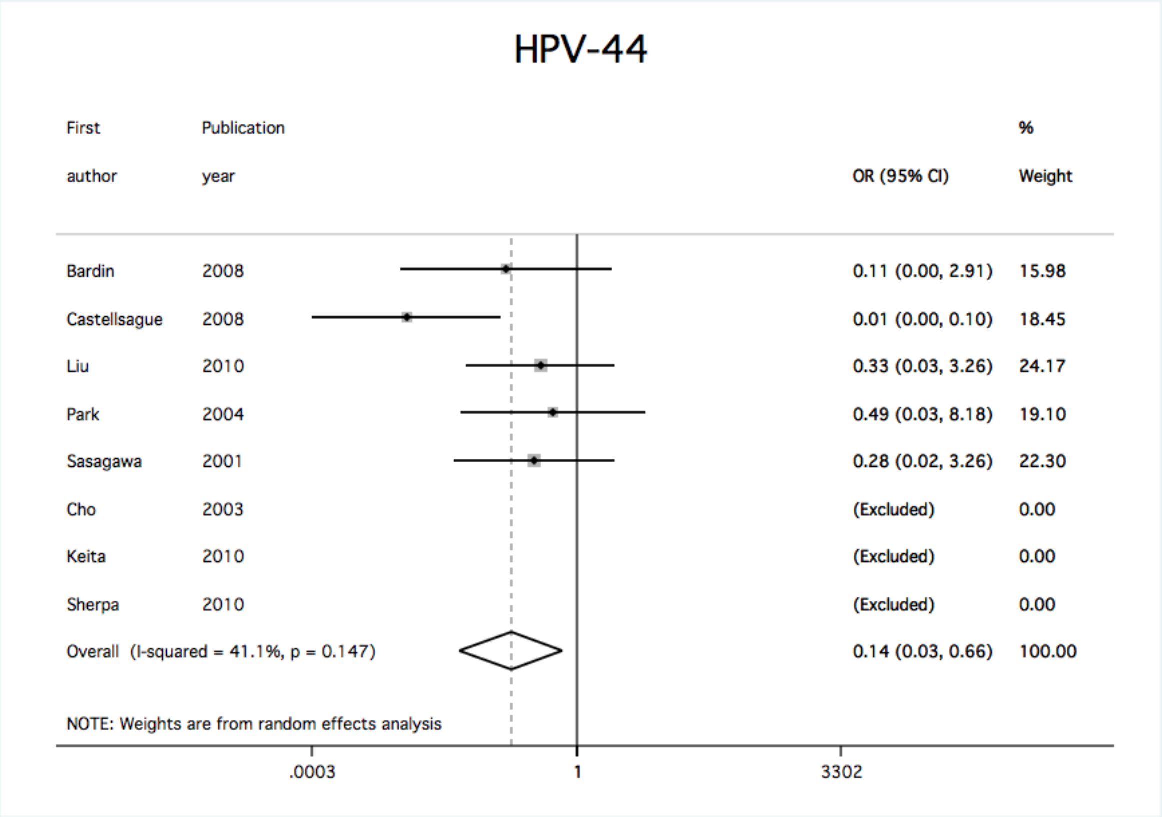
HPV-44
First author
Publication year
OR (95% CI)
% Weight
Bardin 2008 0.11 (0.00, 2.91) 15.98
Castellsague 2008 0.01 (0.00, 0.10) 18.45
Liu 2010 0.33 (0.03, 3.26) 24.17
Park 2004 0.49 (0.03, 8.18) 19.10
Sasagawa 2001 0.28 (0.02, 3.26) 22.30
Cho 2003 (Excluded) 0.00
Keita 2010 (Excluded) 0.00
Sherpa 2010 (Excluded) 0.00
Overall (I-squared = 41.1%, p = 0.147) 0.14 (0.03, 0.66) 100.00
NOTE: Weights are from random effects analysis
.0003
1
3302

(m)

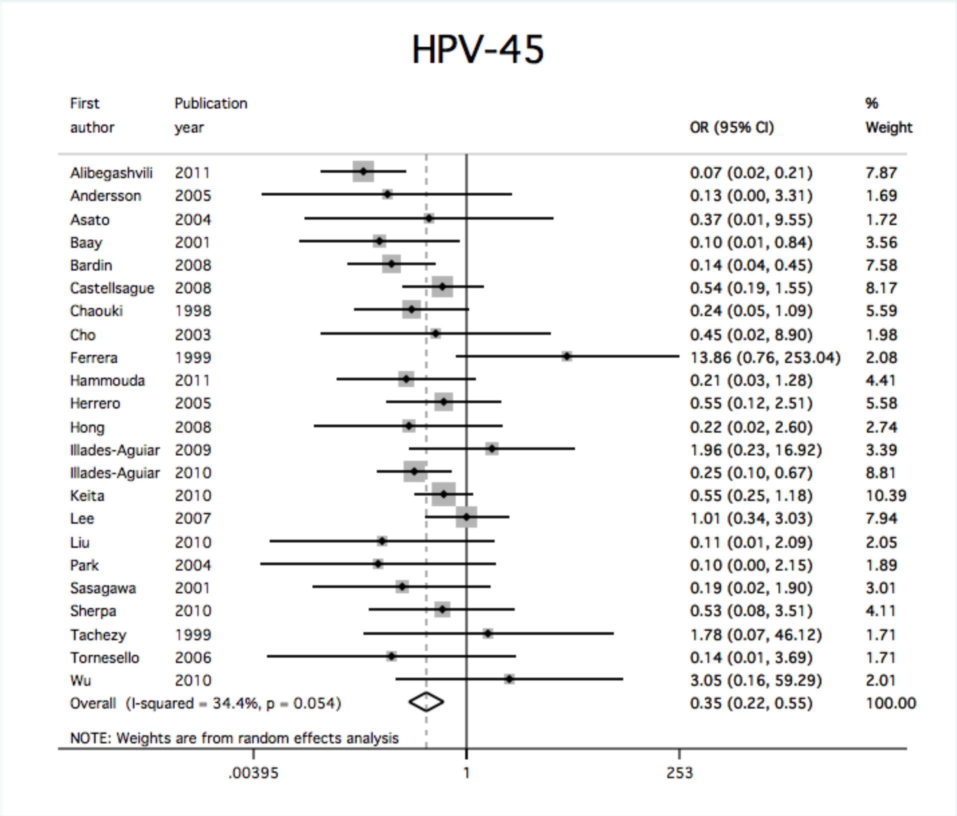
HPV-45
First author
Publication year
OR (95% CI)
% Weight
Alibegashvili 2011 0.07 (0.02, 0.21) 7.87
Andersson 2005 0.13 (0.00, 3.31) 1.69
Asato 2004 0.37 (0.01, 9.55) 1.72
Baay 2001 0.10 (0.01, 0.84) 3.56
Bardin 2008 0.14 (0.04, 0.45) 7.58
Castellsague 2008 0.54 (0.19, 1.55) 8.17
Chaouki 1998 0.24 (0.05, 1.09) 5.59
Cho 2003 0.45 (0.02, 8.90) 1.98
Ferrera 1999 13.86 (0.76, 253.04) 2.08
Hammouda 2011 0.21 (0.03, 1.28) 4.41
Herrero 2005 0.55 (0.12, 2.51) 5.58
Hong 2008 0.22 (0.02, 2.60) 2.74
Illades-Aguiar 2009 1.96 (0.23, 16.92) 3.39
Illades-Aguiar 2010 0.25 (0.10, 0.67) 8.81
Keita 2010 0.55 (0.25, 1.18) 10.39
Lee 2007 1.01 (0.34, 3.03) 7.94
Liu 2010 0.11 (0.01, 2.09) 2.05
Park 2004 0.10 (0.00, 2.15) 1.89
Sasagawa 2001 0.19 (0.02, 1.90) 3.01
Sherpa 2010 0.53 (0.08, 3.51) 4.11
Tachezy 1999 1.78 (0.07, 46.12) 1.71
Tornesello 2006 0.14 (0.01, 3.69) 1.71
Wu 2010 3.05 (0.16, 59.29) 2.01
Overall (I-squared = 34.4%, p = 0.054) 0.35 (0.22, 0.55) 100.00
NOTE: Weights are from random effects analysis
.00395
1
253

(n)

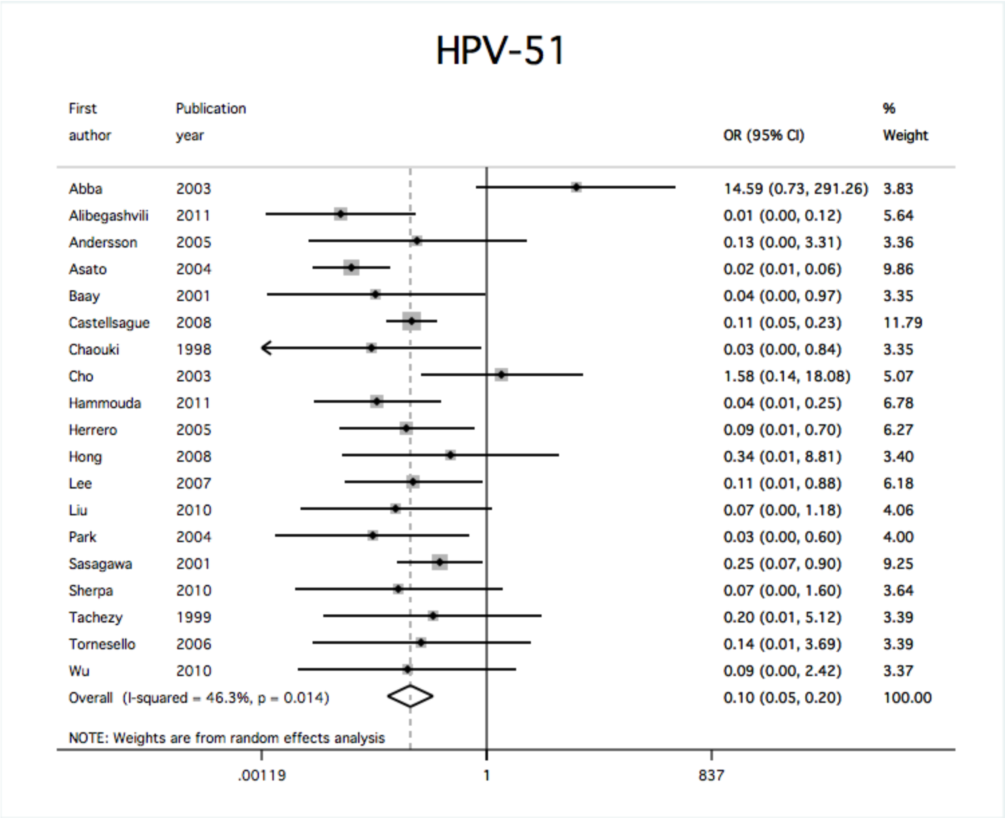
HPV-51
First author
Publication year
OR (95% CI)
% Weight
Abba 2003 14.59 (0.73, 291.26) 3.83
Alibegashvili 2011 0.01 (0.00, 0.12) 5.64
Andersson 2005 0.13 (0.00, 3.31) 3.36
Asato 2004 0.02 (0.01, 0.06) 9.86
Baay 2001 0.04 (0.00, 0.97) 3.35
Castellsague 2008 0.11 (0.05, 0.23) 11.79
Chaouki 1998 0.03 (0.00, 0.84) 3.35
Cho 2003 1.58 (0.14, 18.08) 5.07
Hammouda 2011 0.04 (0.01, 0.25) 6.78
Herrero 2005 0.09 (0.01, 0.70) 6.27
Hong 2008 0.34 (0.01, 8.81) 3.40
Lee 2007 0.11 (0.01, 0.88) 6.18
Liu 2010 0.07 (0.00, 1.18) 4.06
Park 2004 0.03 (0.00, 0.60) 4.00
Sasagawa 2001 0.25 (0.07, 0.90) 9.25
Sherpa 2010 0.07 (0.00, 1.60) 3.64
Tachezy 1999 0.20 (0.01, 5.12) 3.39
Tornesello 2006 0.14 (0.01, 3.69) 3.39
Wu 2010 0.09 (0.00, 2.42) 3.37
Overall (I-squared = 46.3%, p = 0.014) 0.10 (0.05, 0.20) 100.00
NOTE: Weights are from random effects analysis
.00119
1
837

(o)

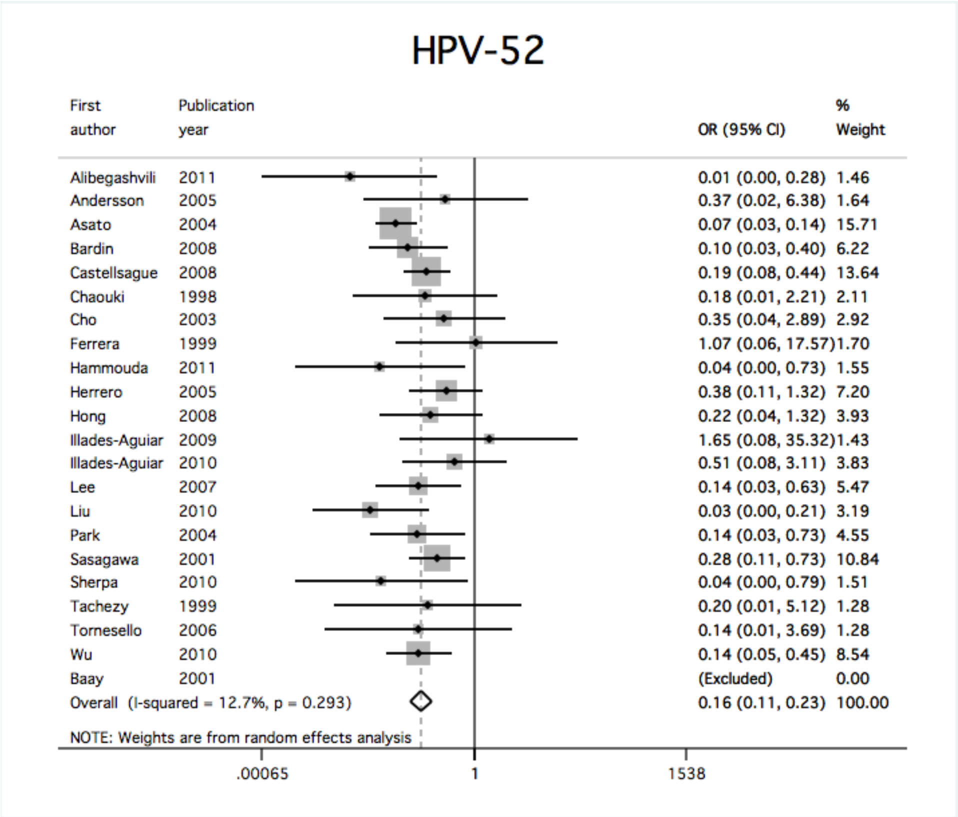
HPV-52
First author
Publication year
OR (95% CI)
% Weight
Alibegashvili 2011 0.01 (0.00, 0.28) 1.46
Andersson 2005 0.37 (0.02, 6.38) 1.64
Asato 2004 0.07 (0.03, 0.14) 15.71
Bardin 2008 0.10 (0.03, 0.40) 6.22
Castellsague 2008 0.19 (0.08, 0.44) 13.64
Chaouki 1998 0.18 (0.01, 2.21) 2.11
Cho 2003 0.35 (0.04, 2.89) 2.92
Ferrera 1999 1.07 (0.06, 17.57)1.70
Hammouda 2011 0.04 (0.00, 0.73) 1.55
Herrero 2005 0.38 (0.11, 1.32) 7.20
Hong 2008 0.22 (0.04, 1.32) 3.93
Illades-Aguiar 2009 1.65 (0.08, 35.32)1.43
Illades-Aguiar 2010 0.51 (0.08, 3.11) 3.83
Lee 2007 0.14 (0.03, 0.63) 5.47
Liu 2010 0.03 (0.00, 0.21) 3.19
Park 2004 0.14 (0.03, 0.73) 4.55
Sasagawa 2001 0.28 (0.11, 0.73) 10.84
Sherpa 2010 0.04 (0.00, 0.79) 1.51
Tachezy 1999 0.20 (0.01, 5.12) 1.28
Tornesello 2006 0.14 (0.01, 3.69) 1.28
Wu 2010 0.14 (0.05, 0.45) 8.54
Baay 2001 (Excluded) 0.00
Overall (I-squared = 12.7%, p = 0.293) 0.16 (0.11, 0.23) 100.00
NOTE: Weights are from random effects analysis
.00065
1
1538

(p)

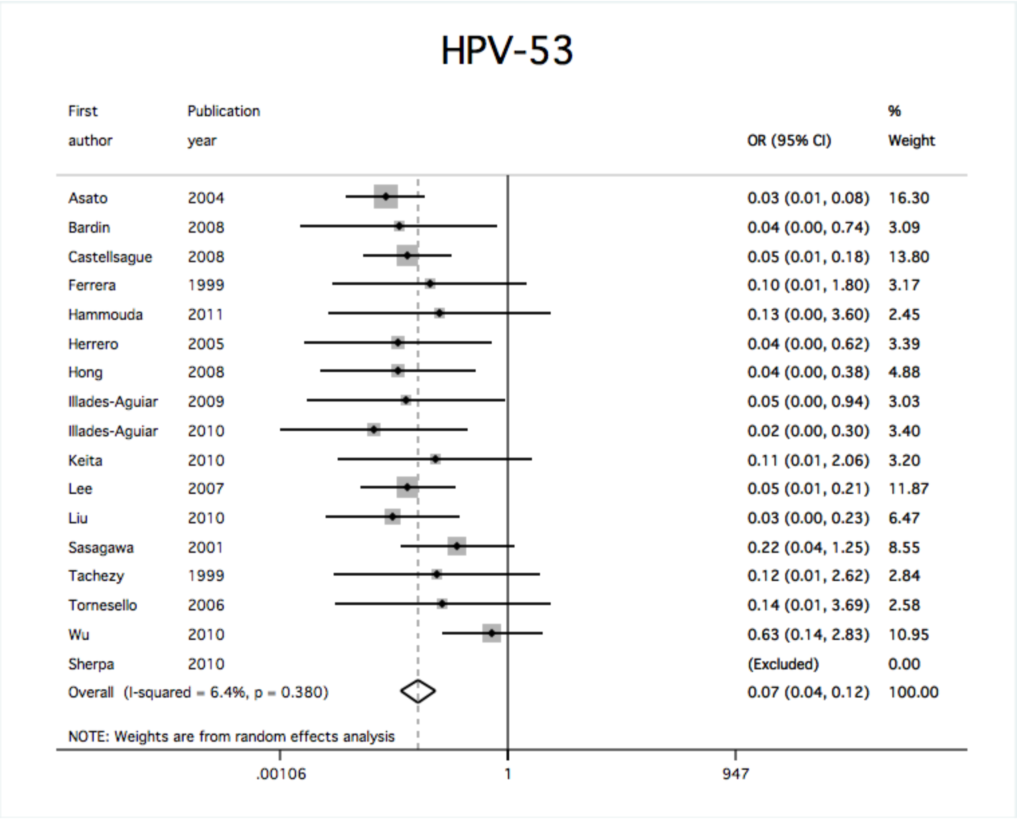
HPV-53
First author
Publication year
OR (95% CI)
% Weight
Asato 2004 0.03 (0.01, 0.08) 16.30
Bardin 2008 0.04 (0.00, 0.74) 3.09
Castellsague 2008 0.05 (0.01, 0.18) 13.80
Ferrera 1999 0.10 (0.01, 1.80) 3.17
Hammouda 2011 0.13 (0.00, 3.60) 2.45
Herrero 2005 0.04 (0.00, 0.62) 3.39
Hong 2008 0.04 (0.00, 0.38) 4.88
Illades-Aguiar 2009 0.05 (0.00, 0.94) 3.03
Illades-Aguiar 2010 0.02 (0.00, 0.30) 3.40
Keita 2010 0.11 (0.01, 2.06) 3.20
Lee 2007 0.05 (0.01, 0.21) 11.87
Liu 2010 0.03 (0.00, 0.23) 6.47
Sasagawa 2001 0.22 (0.04, 1.25) 8.55
Tachezy 1999 0.12 (0.01, 2.62) 2.84
Tornesello 2006 0.14 (0.01, 3.69) 2.58
Wu 2010 0.63 (0.14, 2.83) 10.95
Sherpa 2010 (Excluded) 0.00
Overall (I-squared = 6.4%, p = 0.380) 0.07 (0.04, 0.12) 100.00
NOTE: Weights are from random effects analysis
.00106
1
947

(q)

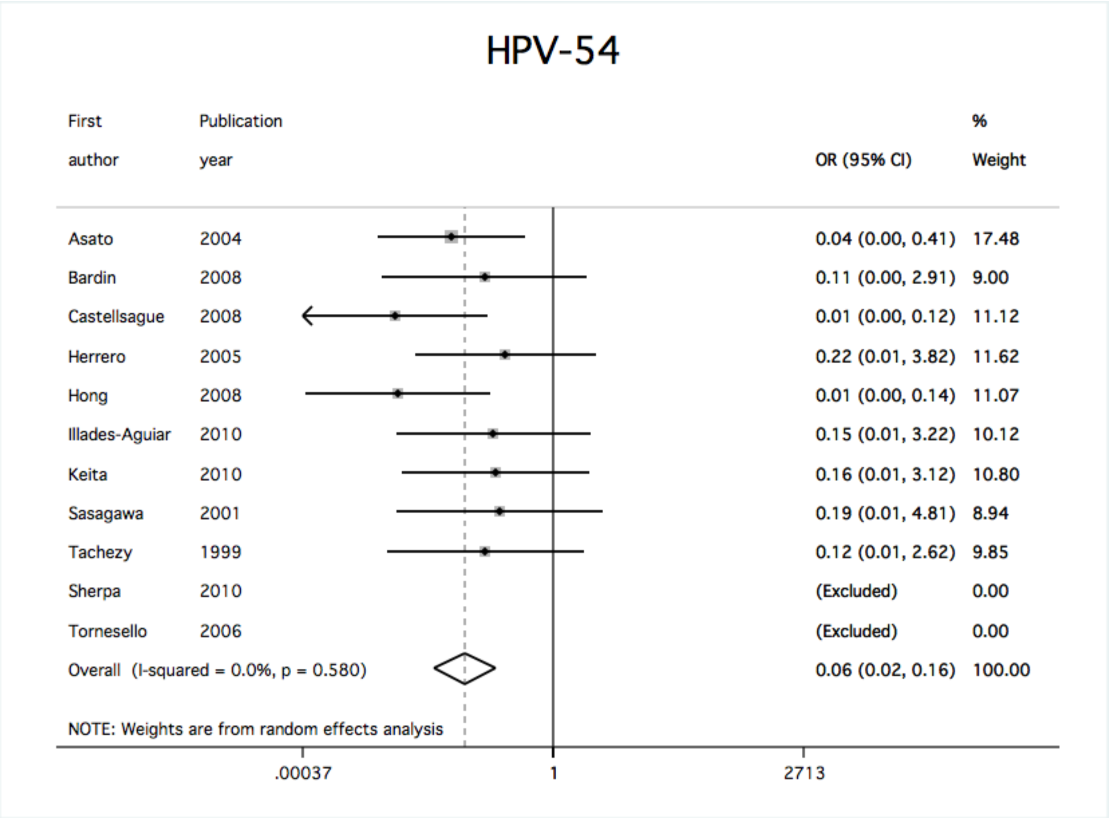
HPV-54
First author
Publication year
OR (95% CI)
% Weight
Asato 2004 0.04 (0.00, 0.41) 17.48
Bardin 2008 0.11 (0.00, 2.91) 9.00
Castellsague 2008 0.01 (0.00, 0.12) 11.12
Herrero 2005 0.22 (0.01, 3.82) 11.62
Hong 2008 0.01 (0.00, 0.14) 11.07
Illades-Aguiar 2010 0.15 (0.01, 3.22) 10.12
Keita 2010 0.16 (0.01, 3.12) 10.80
Sasagawa 2001 0.19 (0.01, 4.81) 8.94
Tachezy 1999 0.12 (0.01, 2.62) 9.85
Sherpa 2010 (Excluded) 0.00
Tornesello 2006 (Excluded) 0.00
Overall (I-squared = 0.0%, p = 0.580) 0.06 (0.02, 0.16) 100.00
NOTE: Weights are from random effects analysis
.00037
1
2713

(r)

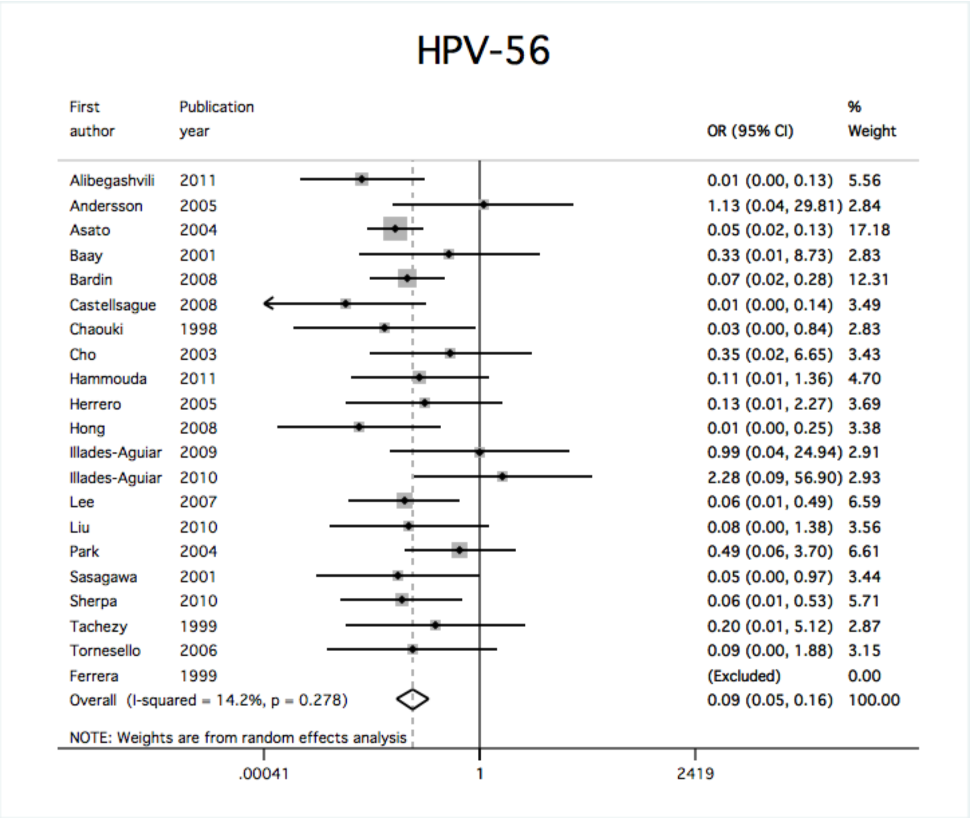
HPV-56
First author
Publication year
OR (95% CI)
% Weight
Alibegashvili 2011 0.01 (0.00, 0.13) 5.56
Andersson 2005 1.13 (0.04, 29.81) 2.84
Asato 2004 0.05 (0.02, 0.13) 17.18
Baay 2001 0.33 (0.01, 8.73) 2.83
Bardin 2008 0.07 (0.02, 0.28) 12.31
Castellsague 2008 0.01 (0.00, 0.14) 3.49
Chaouki 1998 0.03 (0.00, 0.84) 2.83
Cho 2003 0.35 (0.02, 6.65) 3.43
Hammouda 2011 0.11 (0.01, 1.36) 4.70
Herrero 2005 0.13 (0.01, 2.27) 3.69
Hong 2008 0.01 (0.00, 0.25) 3.38
Illades-Aguiar 2009 0.99 (0.04, 24.94) 2.91
Illades-Aguiar 2010 2.28 (0.09, 56.90) 2.93
Lee 2007 0.06 (0.01, 0.49) 6.59
Liu 2010 0.08 (0.00, 1.38) 3.56
Park 2004 0.49 (0.06, 3.70) 6.61
Sasagawa 2001 0.05 (0.00, 0.97) 3.44
Sherpa 2010 0.06 (0.01, 0.53) 5.71
Tachezy 1999 0.20 (0.01, 5.12) 2.87
Tornesello 2006 0.09 (0.00, 1.88) 3.15
Ferrera 1999 (Excluded) 0.00
Overall (I-squared = 14.2%, p = 0.278) 0.09 (0.05, 0.16) 100.00
NOTE: Weights are from random effects analysis
.00041
1
2419

(s)

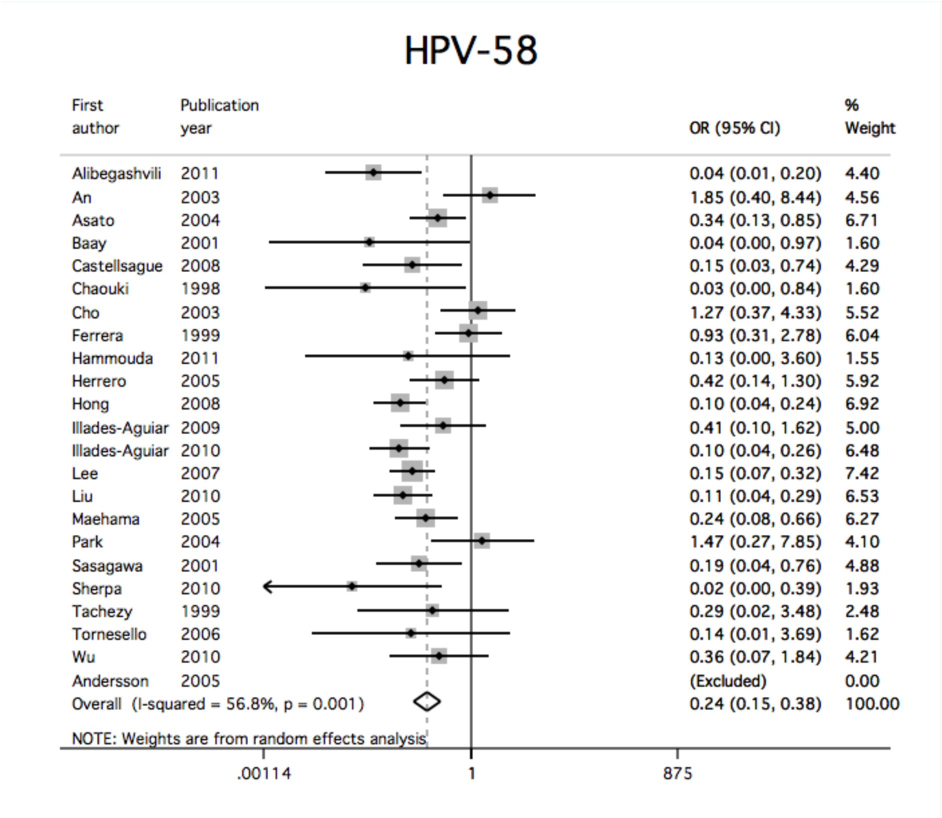
HPV-58
First author
Publication year
OR (95% CI)
% Weight
Alibegashvili 2011 0.04 (0.01, 0.20) 4.40
An 2003 1.85 (0.40, 8.44) 4.56
Asato 2004 0.34 (0.13, 0.85) 6.71
Baay 2001 0.04 (0.00, 0.97) 1.60
Castellsague 2008 0.15 (0.03, 0.74) 4.29
Chaouki 1998 0.03 (0.00, 0.84) 1.60
Cho 2003 1.27 (0.37, 4.33) 5.52
Ferrera 1999 0.93 (0.31, 2.78) 6.04
Hammouda 2011 0.13 (0.00, 3.60) 1.55
Herrero 2005 0.42 (0.14, 1.30) 5.92
Hong 2008 0.10 (0.04, 0.24) 6.92
Illades-Aguiar 2009 0.41 (0.10, 1.62) 5.00
Illades-Aguiar 2010 0.10 (0.04, 0.26) 6.48
Lee 2007 0.15 (0.07, 0.32) 7.42
Liu 2010 0.11 (0.04, 0.29) 6.53
Maehama 2005 0.24 (0.08, 0.66) 6.27
Park 2004 1.47 (0.27, 7.85) 4.10
Sasagawa 2001 0.19 (0.04, 0.76) 4.88
Sherpa 2010 0.02 (0.00, 0.39) 1.93
Tachezy 1999 0.29 (0.02, 3.48) 2.48
Tornesello 2006 0.14 (0.01, 3.69) 1.62
Wu 2010 0.36 (0.07, 1.84) 4.21
Andersson 2005 (Excluded) 0.00
Overall (I-squared = 56.8%, p = 0.001) 0.24 (0.15, 0.38) 100.00
NOTE: Weights are from random effects analysis
.00114
1
875

(t)

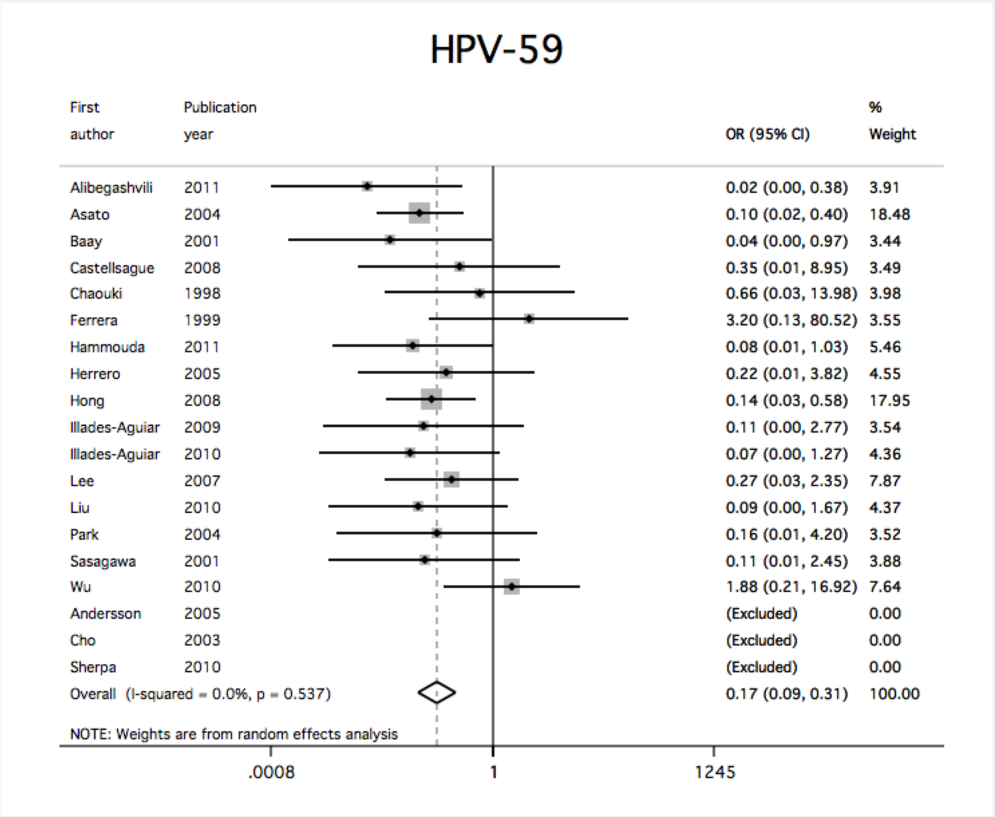
HPV-59
First author
Publication year
OR (95% CI)
% Weight
Alibegashvili 2011 0.02 (0.00, 0.38) 3.91
Asato 2004 0.10 (0.02, 0.40) 18.48
Baay 2001 0.04 (0.00, 0.97) 3.44
Castellsague 2008 0.35 (0.01, 8.95) 3.49
Chaouki 1998 0.66 (0.03, 13.98) 3.98
Ferrera 1999 3.20 (0.13, 80.52) 3.55
Hammouda 2011 0.08 (0.01, 1.03) 5.46
Herrero 2005 0.22 (0.01, 3.82) 4.55
Hong 2008 0.14 (0.03, 0.58) 17.95
Illades-Aguiar 2009 0.11 (0.00, 2.77) 3.54
Illades-Aguiar 2010 0.07 (0.00, 1.27) 4.36
Lee 2007 0.27 (0.03, 2.35) 7.87
Liu 2010 0.09 (0.00, 1.67) 4.37
Park 2004 0.16 (0.01, 4.20) 3.52
Sasagawa 2001 0.11 (0.01, 2.45) 3.88
Wu 2010 1.88 (0.21, 16.92) 7.64
Andersson 2005 (Excluded) 0.00
Cho 2003 (Excluded) 0.00
Sherpa 2010 (Excluded) 0.00
Overall (I-squared = 0.0%, p = 0.537) 0.17 (0.09, 0.31) 100.00
NOTE: Weights are from random effects analysis
.0008
1
1245

(u)

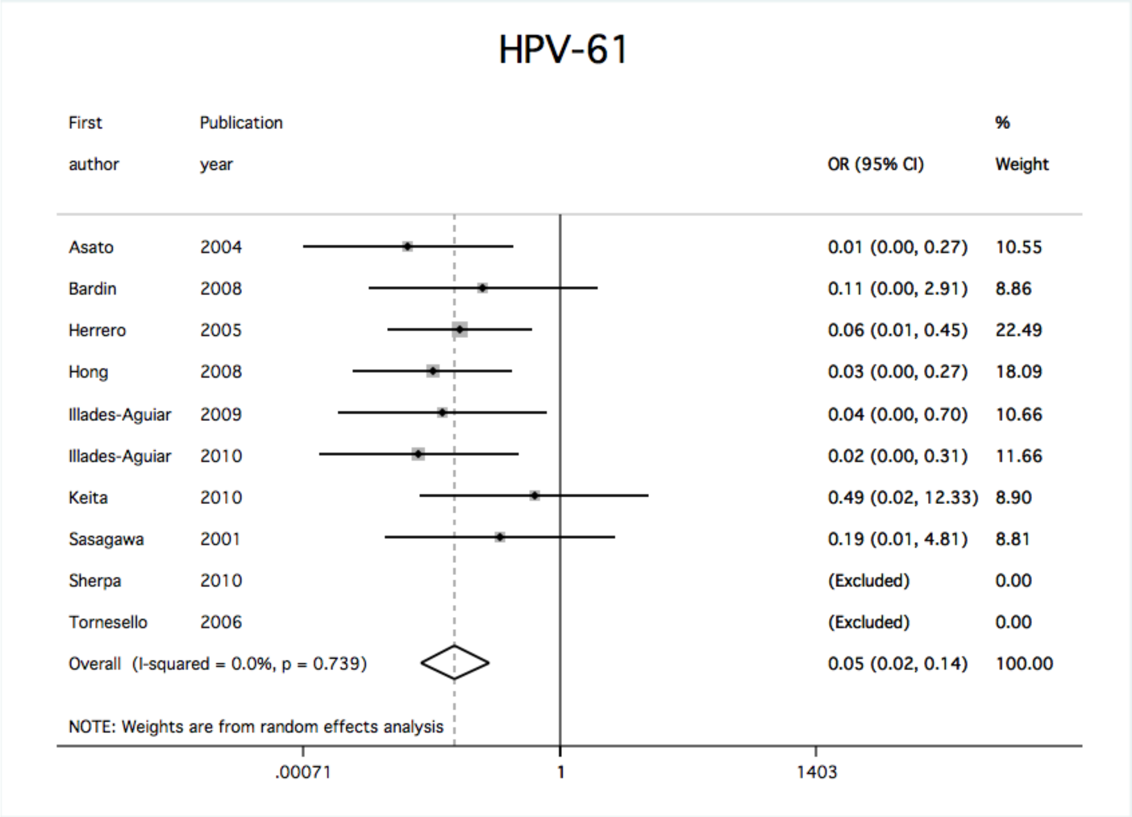
HPV-61
First author
Publication year
OR (95% CI)
% Weight
Asato 2004 0.01 (0.00, 0.27) 10.55
Bardin 2008 0.11 (0.00, 2.91) 8.86
Herrero 2005 0.06 (0.01, 0.45) 22.49
Hong 2008 0.03 (0.00, 0.27) 18.09
Illades-Aguiar 2009 0.04 (0.00, 0.70) 10.66
Illades-Aguiar 2010 0.02 (0.00, 0.31) 11.66
Keita 2010 0.49 (0.02, 12.33) 8.90
Sasagawa 2001 0.19 (0.01, 4.81) 8.81
Sherpa 2010 (Excluded) 0.00
Tornesello 2006 (Excluded) 0.00
Overall (I-squared = 0.0%, p = 0.739) 0.05 (0.02, 0.14) 100.00
NOTE: Weights are from random effects analysis
.00071
1
1403

(v)

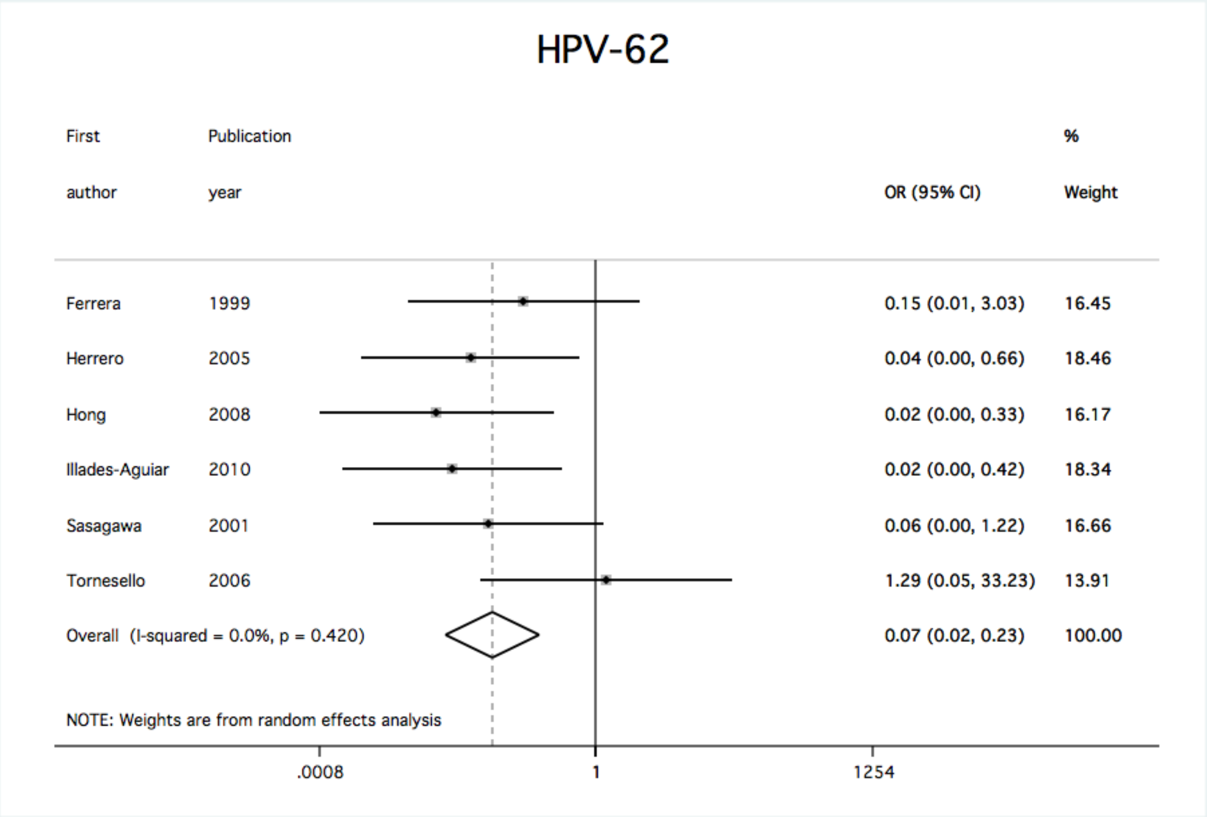
HPV-62
First author
Publication year
OR (95% CI)
% Weight
Ferrera 1999 0.15 (0.01, 3.03) 16.45
Herrero 2005 0.04 (0.00, 0.66) 18.46
Hong 2008 0.02 (0.00, 0.33) 16.17
Illades-Aguiar 2010 0.02 (0.00, 0.42) 18.34
Sasagawa 2001 0.06 (0.00, 1.22) 16.66
Tornesello 2006 1.29 (0.05, 33.23) 13.91
Overall (I-squared = 0.0%, p = 0.420) 0.07 (0.02, 0.23) 100.00
NOTE: Weights are from random effects analysis
.0008
1
1254

(w)

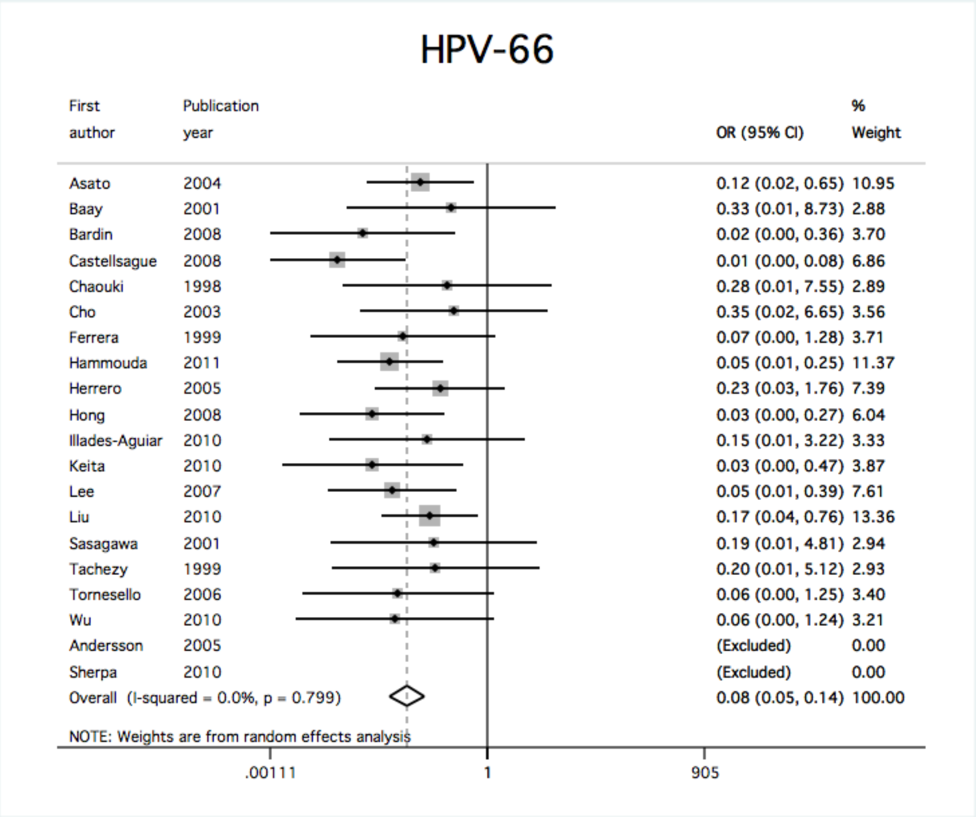
HPV-66
First author
Publication year
OR (95% CI)
% Weight
Asato 2004 0.12 (0.02, 0.65) 10.95
Baay 2001 0.33 (0.01, 8.73) 2.88
Bardin 2008 0.02 (0.00, 0.36) 3.70
Castellsague 2008 0.01 (0.00, 0.08) 6.86
Chaouki 1998 0.28 (0.01, 7.55) 2.89
Cho 2003 0.35 (0.02, 6.65) 3.56
Ferrera 1999 0.07 (0.00, 1.28) 3.71
Hammouda 2011 0.05 (0.01, 0.25) 11.37
Herrero 2005 0.23 (0.03, 1.76) 7.39
Hong 2008 0.03 (0.00, 0.27) 6.04
Illades-Aguiar 2010 0.15 (0.01, 3.22) 3.33
Keita 2010 0.03 (0.00, 0.47) 3.87
Lee 2007 0.05 (0.01, 0.39) 7.61
Liu 2010 0.17 (0.04, 0.76) 13.36
Sasagawa 2001 0.19 (0.01, 4.81) 2.94
Tachezy 1999 0.20 (0.01, 5.12) 2.93
Tornesello 2006 0.06 (0.00, 1.25) 3.40
Wu 2010 0.06 (0.00, 1.24) 3.21
Andersson 2005 (Excluded) 0.00
Sherpa 2010 (Excluded) 0.00
Overall (I-squared = 0.0%, p = 0.799) 0.08 (0.05, 0.14) 100.00
NOTE: Weights are from random effects analysis
.00111
1
905

(x)

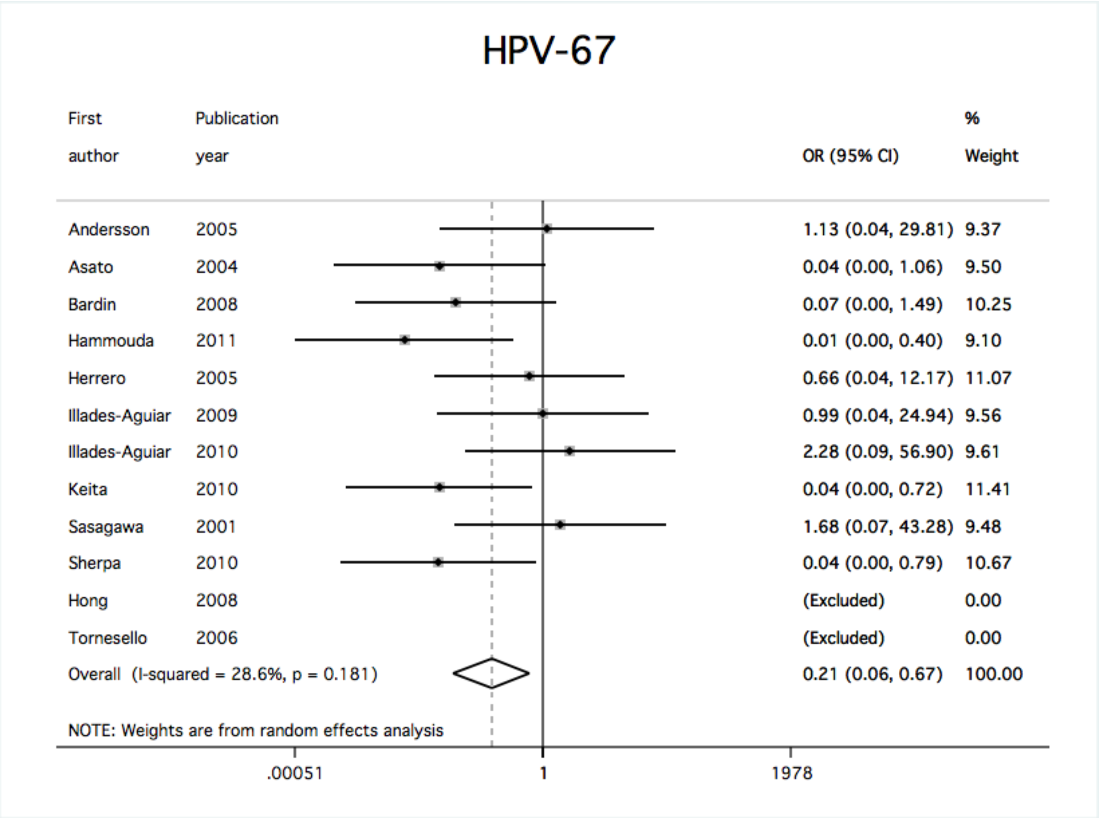
HPV-67
First author
Publication year
OR (95% CI)
% Weight
Andersson 2005 1.13 (0.04, 29.81) 9.37
Asato 2004 0.04 (0.00, 1.06) 9.50
Bardin 2008 0.07 (0.00, 1.49) 10.25
Hammouda 2011 0.01 (0.00, 0.40) 9.10
Herrero 2005 0.66 (0.04, 12.17) 11.07
Illades-Aguiar 2009 0.99 (0.04, 24.94) 9.56
Illades-Aguiar 2010 2.28 (0.09, 56.90) 9.61
Keita 2010 0.04 (0.00, 0.72) 11.41
Sasagawa 2001 1.68 (0.07, 43.28) 9.48
Sherpa 2010 0.04 (0.00, 0.79) 10.67
Hong 2008 (Excluded) 0.00
Tornesello 2006 (Excluded) 0.00
Overall (I-squared = 28.6%, p = 0.181) 0.21 (0.06, 0.67) 100.00
NOTE: Weights are from random effects analysis
.00051
1
1978

(y)

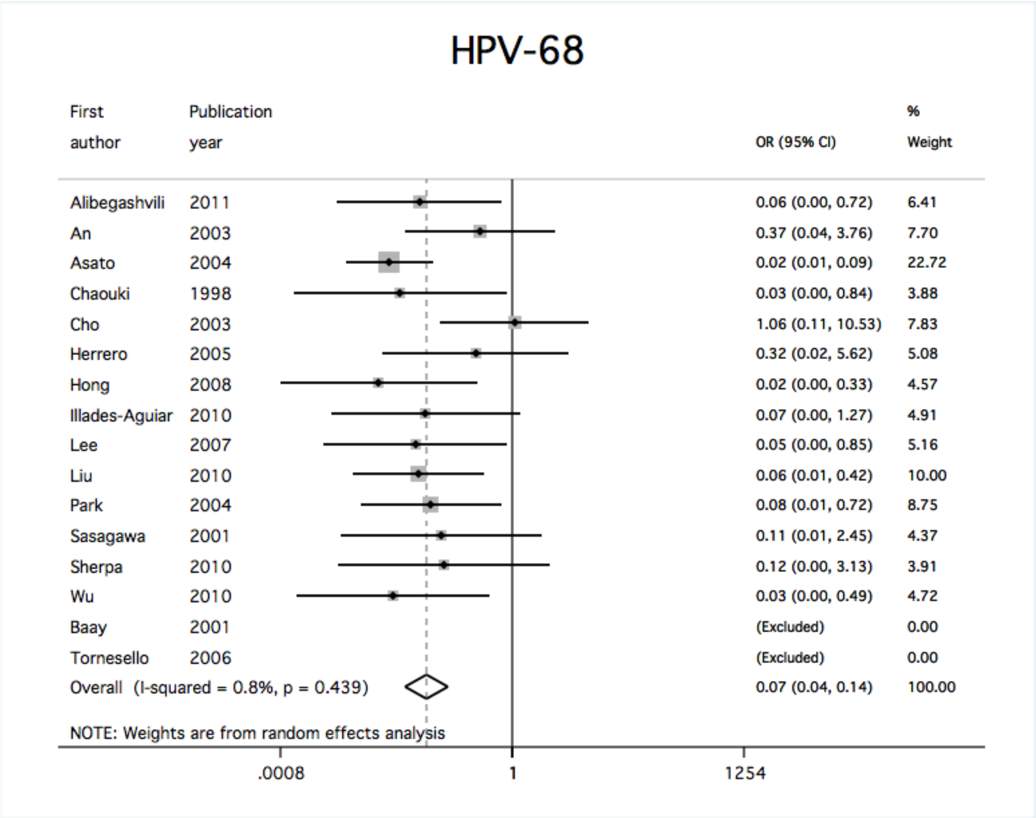
HPV-68
First author
Publication year
OR (95% CI)
% Weight
Alibegashvili 2011 0.06 (0.00, 0.72) 6.41
An 2003 0.37 (0.04, 3.76) 7.70
Asato 2004 0.02 (0.01, 0.09) 22.72
Chaouki 1998 0.03 (0.00, 0.84) 3.88
Cho 2003 1.06 (0.11, 10.53) 7.83
Herrero 2005 0.32 (0.02, 5.62) 5.08
Hong 2008 0.02 (0.00, 0.33) 4.57
Illades-Aguiar 2010 0.07 (0.00, 1.27) 4.91
Lee 2007 0.05 (0.00, 0.85) 5.16
Liu 2010 0.06 (0.01, 0.42) 10.00
Park 2004 0.08 (0.01, 0.72) 8.75
Sasagawa 2001 0.11 (0.01, 2.45) 4.37
Sherpa 2010 0.12 (0.00, 3.13) 3.91
Wu 2010 0.03 (0.00, 0.49) 4.72
Baay 2001 (Excluded) 0.00
Tornesello 2006 (Excluded) 0.00
Overall (I-squared = 0.8%, p = 0.439) 0.07 (0.04, 0.14) 100.00
NOTE: Weights are from random effects analysis
.0008
1
1254

(z)

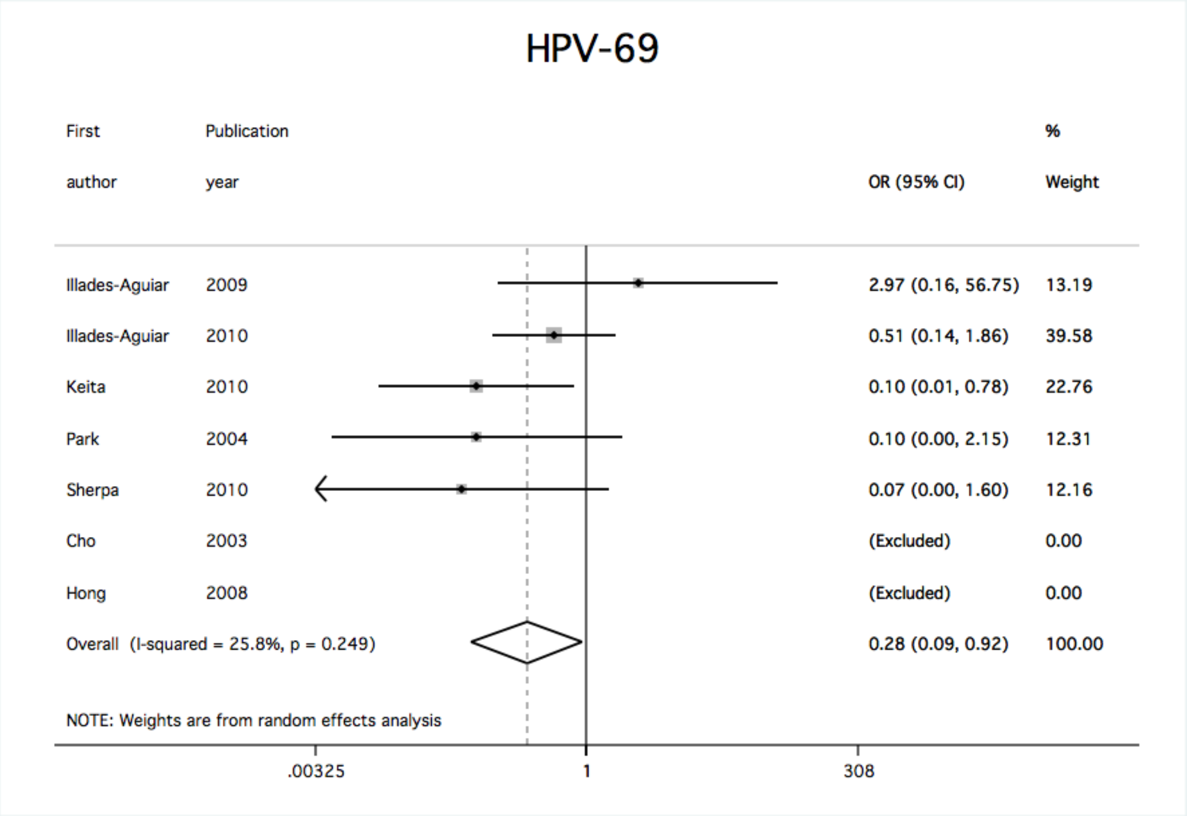
HPV-69
First author
Publication year
OR (95% CI)
% Weight
Illades-Aguiar 2009 2.97 (0.16, 56.75) 13.19
Illades-Aguiar 2010 0.51 (0.14, 1.86) 39.58
Keita 2010 0.10 (0.01, 0.78) 22.76
Park 2004 0.10 (0.00, 2.15) 12.31
Sherpa 2010 0.07 (0.00, 1.60) 12.16
Cho 2003 (Excluded) 0.00
Hong 2008 (Excluded) 0.00
Overall (I-squared = 25.8%, p = 0.249) 0.28 (0.09, 0.92) 100.00
NOTE: Weights are from random effects analysis
.00325
1
308

(aa)

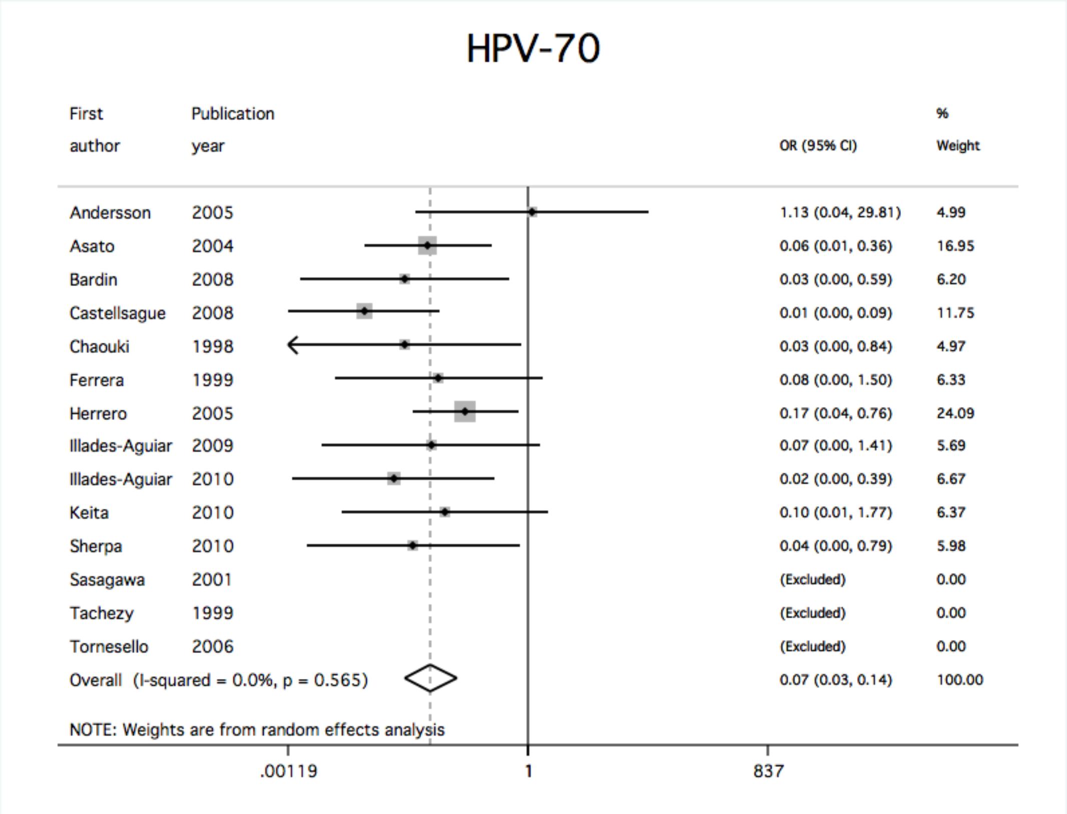
HPV-70
First author
Publication year
OR (95% CI)
% Weight
Andersson 2005 1.13 (0.04, 29.81) 4.99
Asato 2004 0.06 (0.01, 0.36) 16.95
Bardin 2008 0.03 (0.00, 0.59) 6.20
Castellsague 2008 0.01 (0.00, 0.09) 11.75
Chaouki 1998 0.03 (0.00, 0.84) 4.97
Ferrera 1999 0.08 (0.00, 1.50) 6.33
Herrero 2005 0.17 (0.04, 0.76) 24.09
Illades-Aguiar 2009 0.07 (0.00, 1.41) 5.69
Illades-Aguiar 2010 0.02 (0.00, 0.39) 6.67
Keita 2010 0.10 (0.01, 1.77) 6.37
Sherpa 2010 0.04 (0.00, 0.79) 5.98
Sasagawa 2001 (Excluded) 0.00
Tachezy 1999 (Excluded) 0.00
Tornesello 2006 (Excluded) 0.00
Overall (I-squared = 0.0%, p = 0.565) 0.07 (0.03, 0.14) 100.00
NOTE: Weights are from random effects analysis
.00119
1
837

(ab)

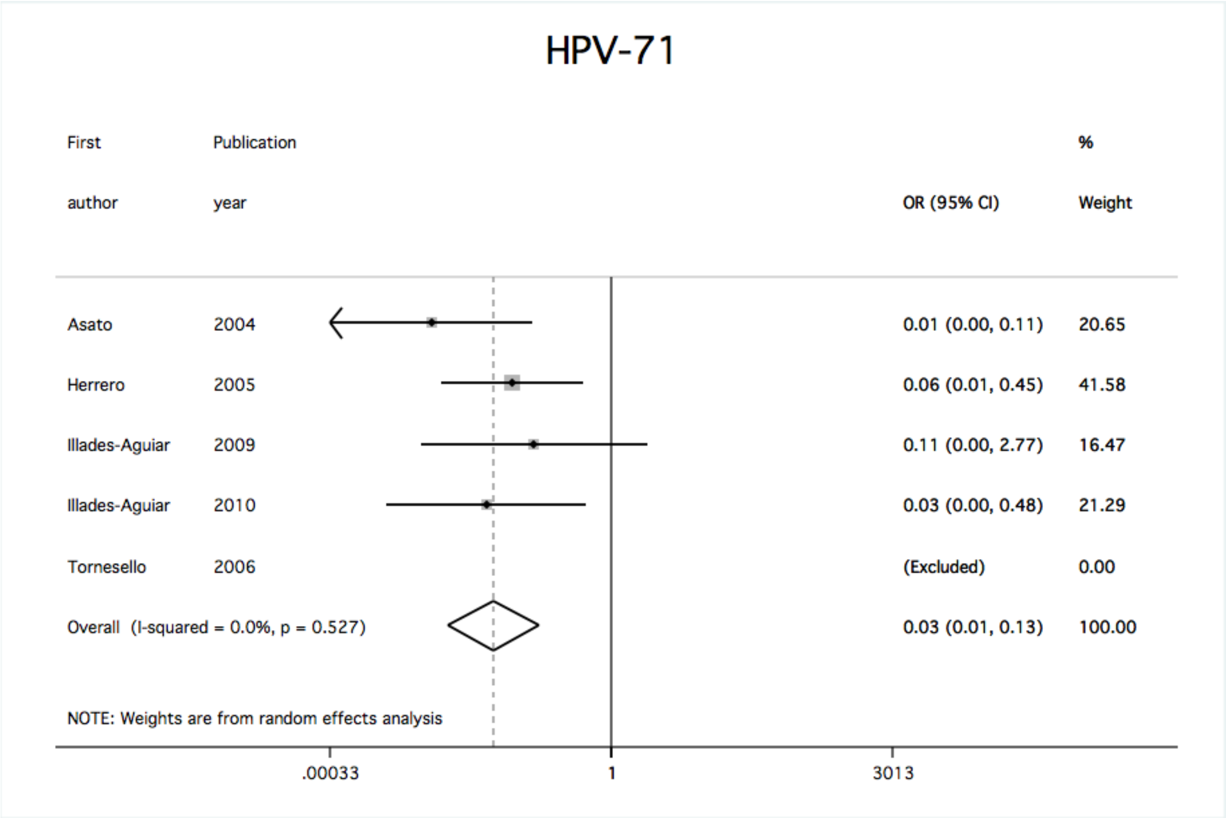
HPV-71
First author
Publication year
OR (95% CI)
% Weight
Asato 2004 0.01 (0.00, 0.11) 20.65
Herrero 2005 0.06 (0.01, 0.45) 41.58
Illades-Aguiar 2009 0.11 (0.00, 2.77) 16.47
Illades-Aguiar 2010 0.03 (0.00, 0.48) 21.29
Tornesello 2006 (Excluded) 0.00
Overall (I-squared = 0.0%, p = 0.527) 0.03 (0.01, 0.13) 100.00
NOTE: Weights are from random effects analysis
.00033
1
3013

(ac)

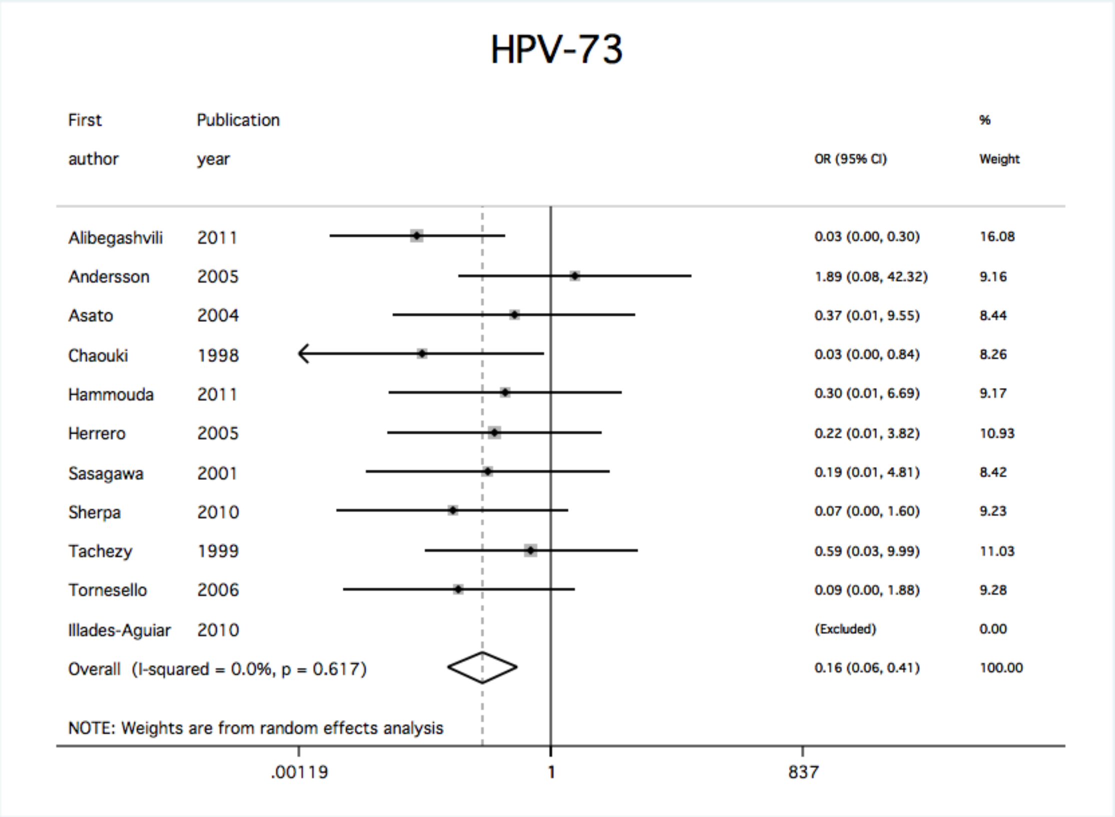
HPV-73
First author
Publication year
OR (95% CI)
% Weight
Alibegashvili 2011 0.03 (0.00, 0.30) 16.08
Andersson 2005 1.89 (0.08, 42.32) 9.16
Asato 2004 0.37 (0.01, 9.55) 8.44
Chaouki 1998 0.03 (0.00, 0.84) 8.26
Hammouda 2011 0.30 (0.01, 6.69) 9.17
Herrero 2005 0.22 (0.01, 3.82) 10.93
Sasagawa 2001 0.19 (0.01, 4.81) 8.42
Sherpa 2010 0.07 (0.00, 1.60) 9.23
Tachezy 1999 0.59 (0.03, 9.99) 11.03
Tornesello 2006 0.09 (0.00, 1.88) 9.28
Illades-Aguiar 2010 (Excluded) 0.00
Overall (I-squared = 0.0%, p = 0.617) 0.16 (0.06, 0.41) 100.00
NOTE: Weights are from random effects analysis
.00119
1
837

(ad)

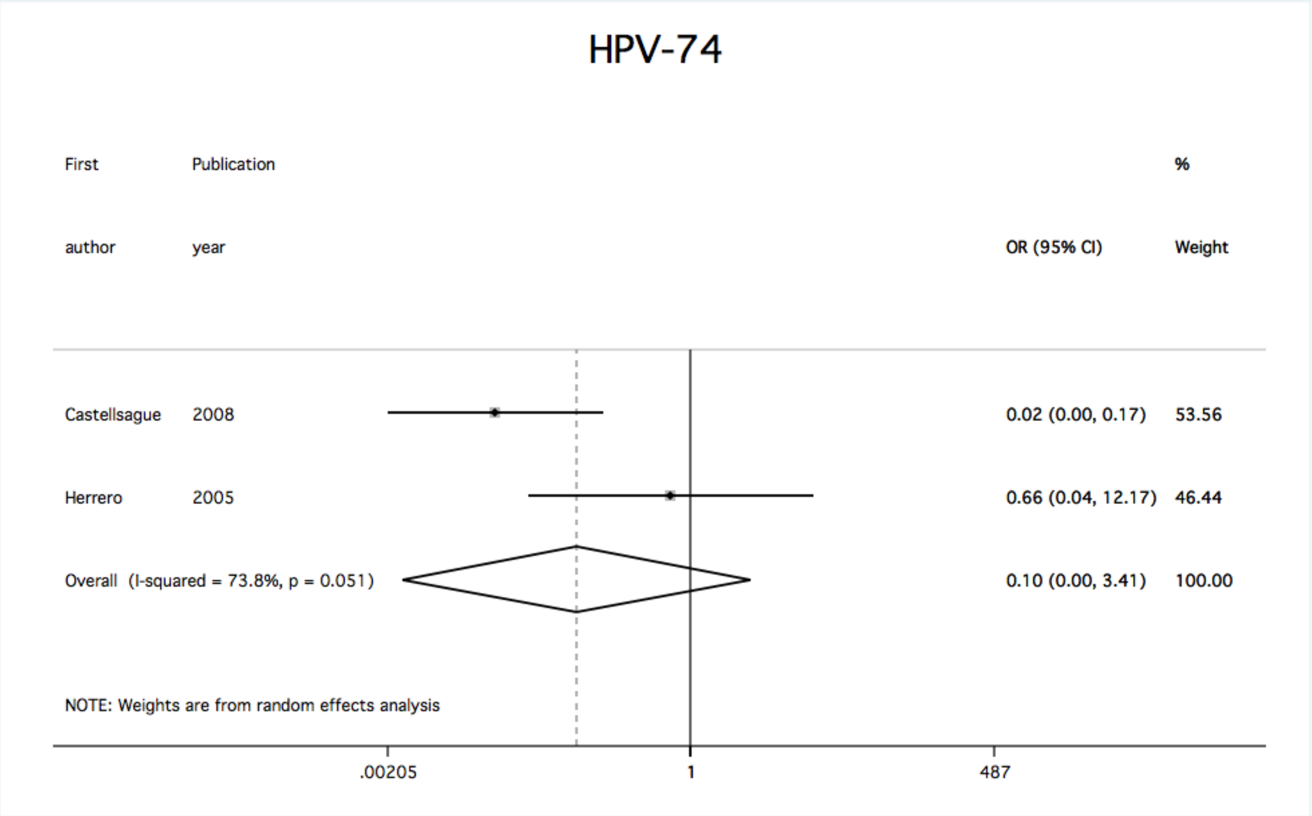
HPV-74
First author
Publication year
OR (95% CI)
% Weight
Castellsague 2008 0.02 (0.00, 0.17) 53.56
Herrero 2005 0.66 (0.04, 12.17) 46.44
Overall (I-squared = 73.8%, p = 0.051) 0.10 (0.00, 3.41) 100.00
NOTE: Weights are from random effects analysis
.00205
1
487

(ae)

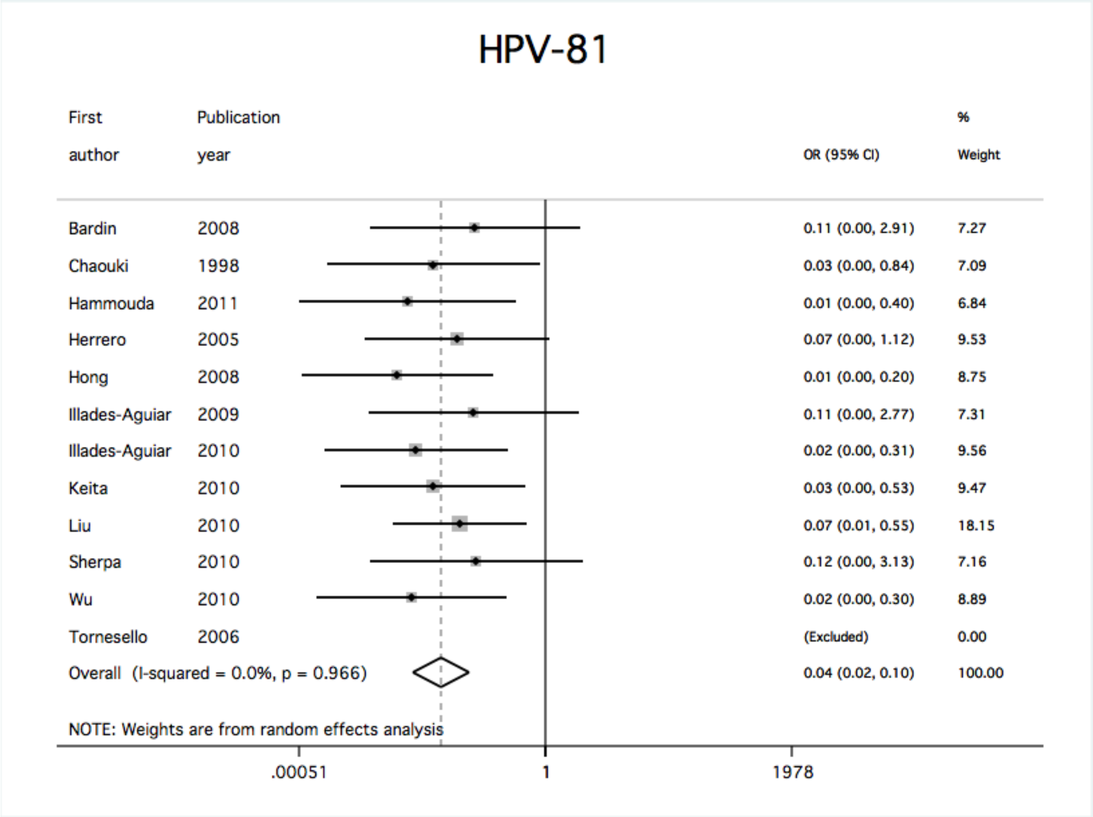
HPV-81
First author
Publication year
OR (95% CI)
% Weight
Bardin 2008 0.11 (0.00, 2.91) 7.27
Chaouki 1998 0.03 (0.00, 0.84) 7.09
Hammouda 2011 0.01 (0.00, 0.40) 6.84
Herrero 2005 0.07 (0.00, 1.12) 9.53
Hong 2008 0.01 (0.00, 0.20) 8.75
Illades-Aguiar 2009 0.11 (0.00, 2.77) 7.31
Illades-Aguiar 2010 0.02 (0.00, 0.31) 9.56
Keita 2010 0.03 (0.00, 0.53) 9.47
Liu 2010 0.07 (0.01, 0.55) 18.15
Sherpa 2010 0.12 (0.00, 3.13) 7.16
Wu 2010 0.02 (0.00, 0.30) 8.89
Tornesello 2006 (Excluded) 0.00
Overall (I-squared = 0.0%, p = 0.966) 0.04 (0.02, 0.10) 100.00
NOTE: Weights are from random effects analysis
.00051
1
1978

(af)

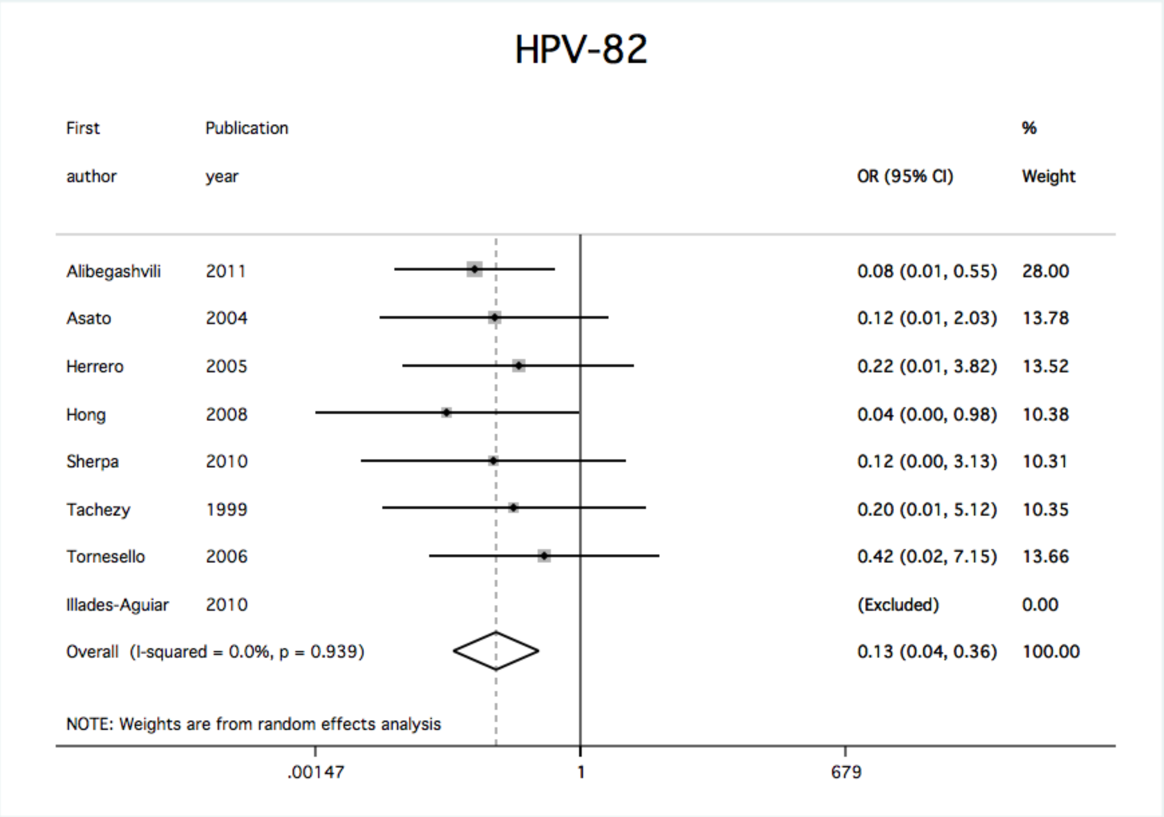
HPV-82
First author
Publication year
OR (95% CI)
% Weight
Alibegashvili 2011 0.08 (0.01, 0.55) 28.00
Asato 2004 0.12 (0.01, 2.03) 13.78
Herrero 2005 0.22 (0.01, 3.82) 13.52
Hong 2008 0.04 (0.00, 0.98) 10.38
Sherpa 2010 0.12 (0.00, 3.13) 10.31
Tachezy 1999 0.20 (0.01, 5.12) 10.35
Tornesello 2006 0.42 (0.02, 7.15) 13.66
Illades-Aguiar 2010 (Excluded) 0.00
Overall (I-squared = 0.0%, p = 0.939) 0.13 (0.04, 0.36) 100.00
NOTE: Weights are from random effects analysis
.00147
1
679
