# Supplementary material for: Comparing human papillomavirus prevalences in women with normal cytology or invasive cervical cancer to rank genotypes according to their oncogenic potential: a meta-analysis of observational studies
Source: BMC Infect Dis. 2013 Aug 13;13:373. doi: 10.1186/1471-2334-13-373 (PMC3751808; doi:10.1186/1471-2334-13-373)
Supplement: Additional file 3 — Bias assessment for each meta-analysis (funnel plots). Each dot represents one study. The solid vertical line is the pooled odds ratio (OR). Diagonal dashed lines represent the pseudo 95% confidence limits around the pooled OR for each standard error of the ordinate vertical axis values, defining a funnel within which 95% of the studies should lie in the absence of heterogeneity or selection biases. The yellow line is the fitted linear-regression line of the OR plotted against its standard error (both on natural logarithm scales) and corresponds to Egger’s test for funnel-plot asymmetry. The graphs were generated by the Stata command metafunnel (adapted from [29] pp 113 and 115). [file 1471-2334-13-373-S3.pdf]

Additional File 3. Bias assessment for each meta-analysis (funnel plots). Each dot represents one study. The solid vertical line is the pooled odds ratio (OR). Diagonal dashed lines represent the pseudo 95% confidence limits around the pooled OR for each standard error of the ordinate vertical axis values, defining a funnel within which 95% of the studies should lie in the absence of heterogeneity or selection biases. The yellow line is the fitted linear-regression line of the OR plotted against its standard error (both on natural logarithm scales) and corresponds to Egger's test for funnel-plot asymmetry. The graphs were generated by the Stata command metafunnel .

(a)

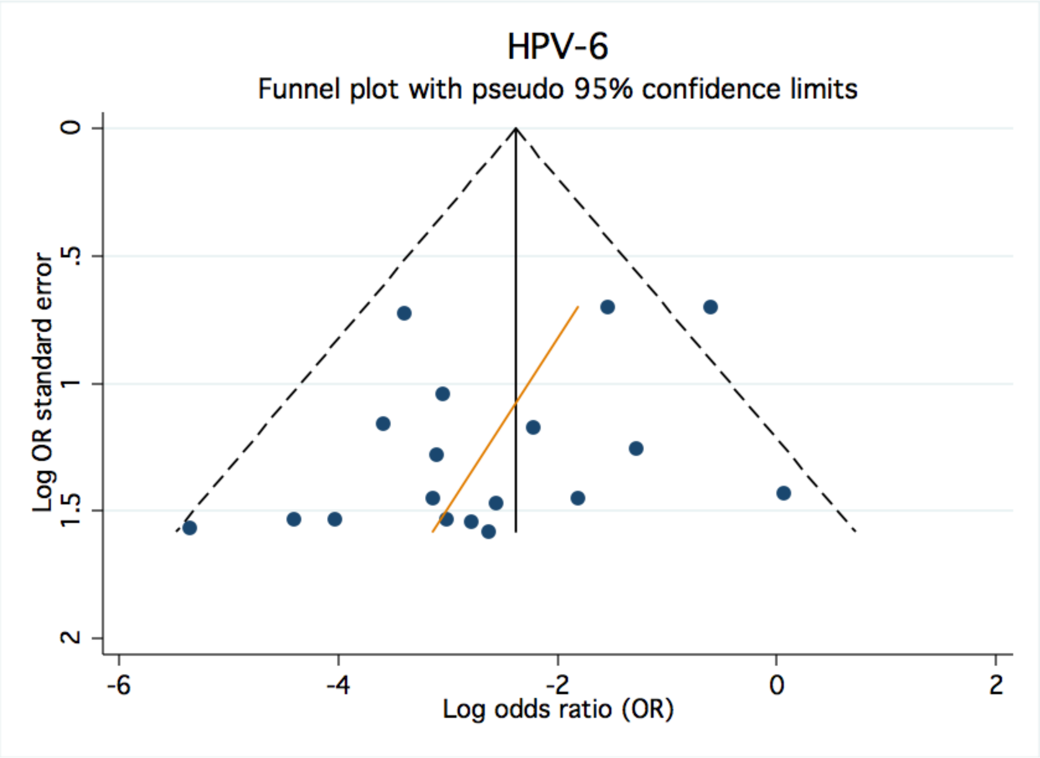

(b)

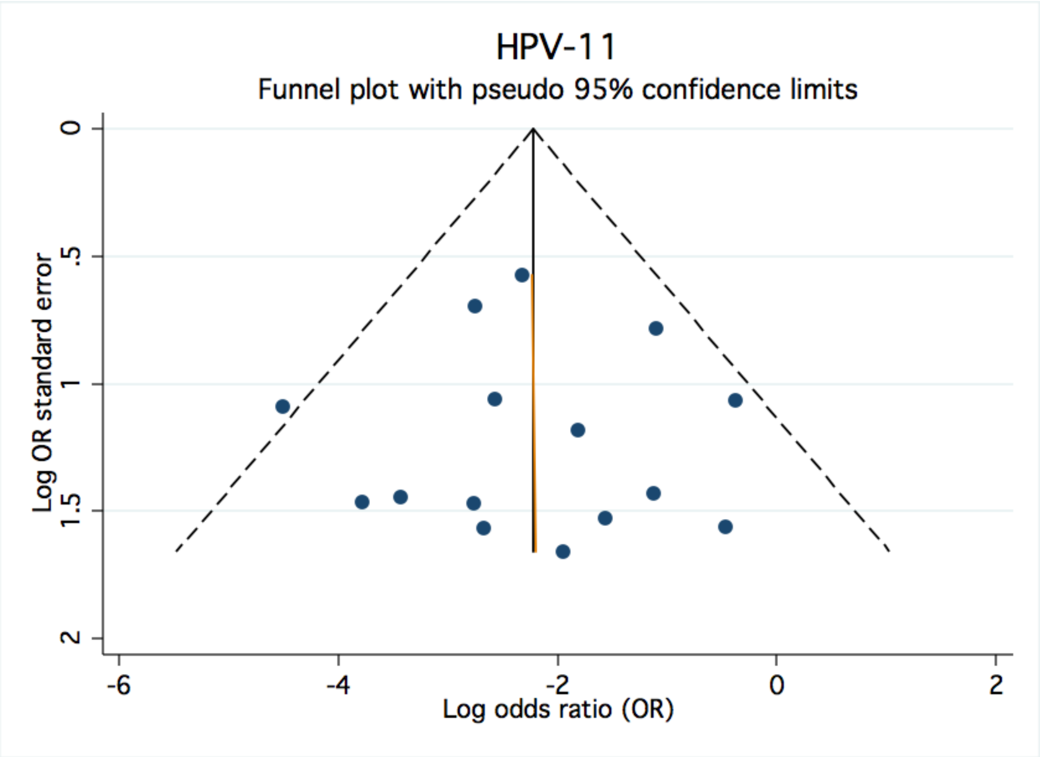
HPV-11
Funnel plot with pseudo 95% confidence limits
Log OR standard error
0
.5
1
1.5
2
-6
-4
-2
0
2
Log odds ratio (OR)

(c)

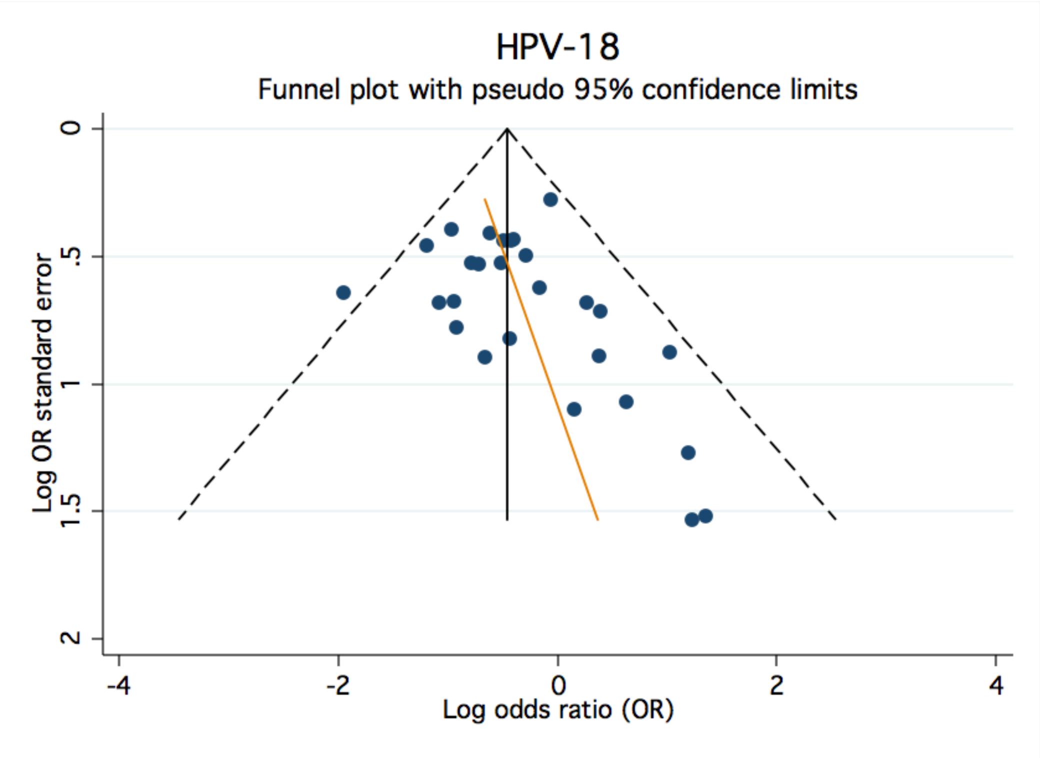
HPV-18
Funnel plot with pseudo 95% confidence limits
Log OR standard error
0
.5
1
1.5
2
-4
-2
0
2
4
Log odds ratio (OR)

(d)

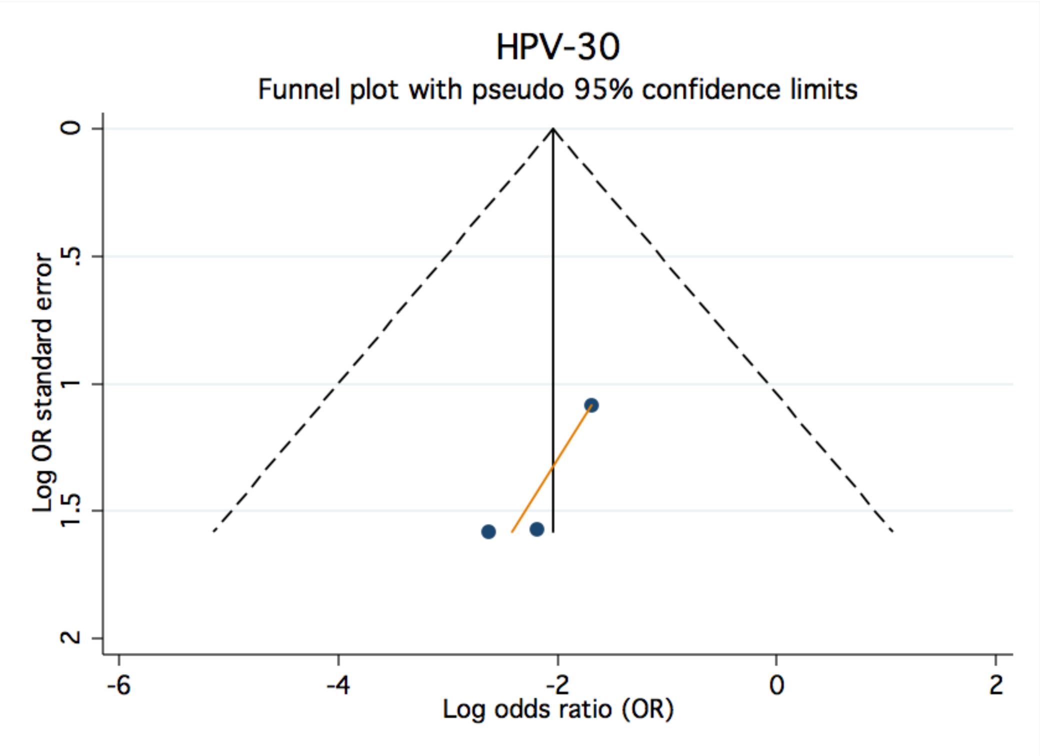
HPV-30
Funnel plot with pseudo 95% confidence limits
Log OR standard error
0
.5
1
1.5
2
-6
-4
-2
0
2
Log odds ratio (OR)

(e)

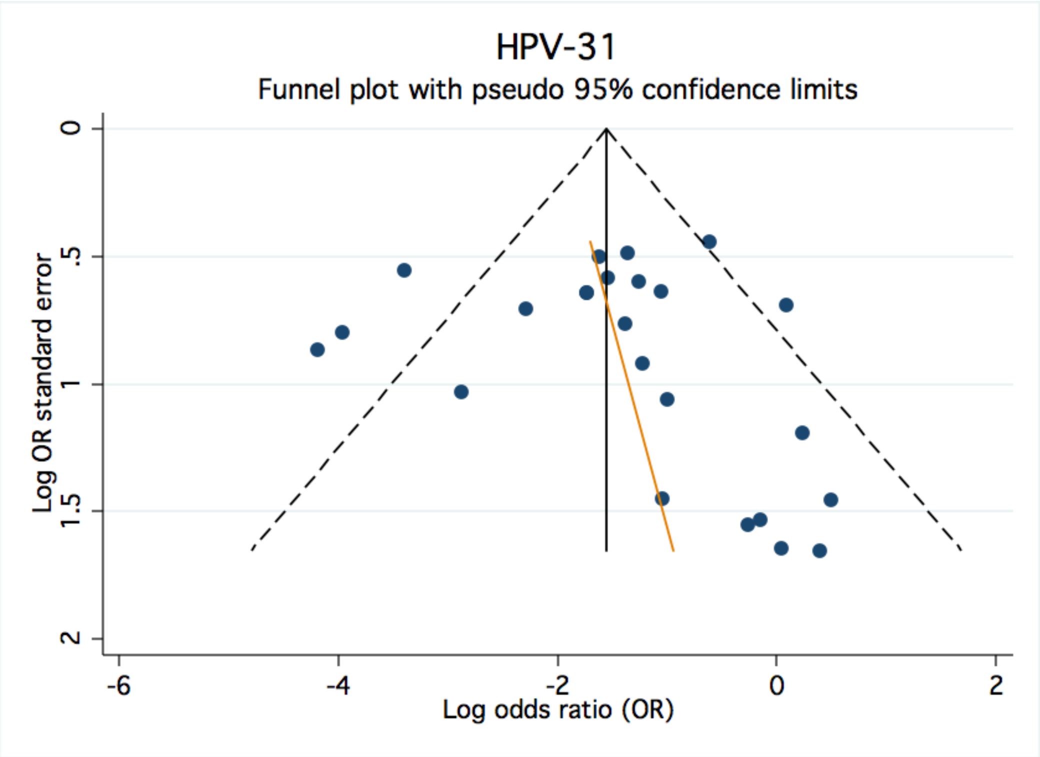
HPV-31
Funnel plot with pseudo 95% confidence limits
Log OR standard error
0
.5
1
1.5
2
-6
-4
-2
0
2
Log odds ratio (OR)

(f)

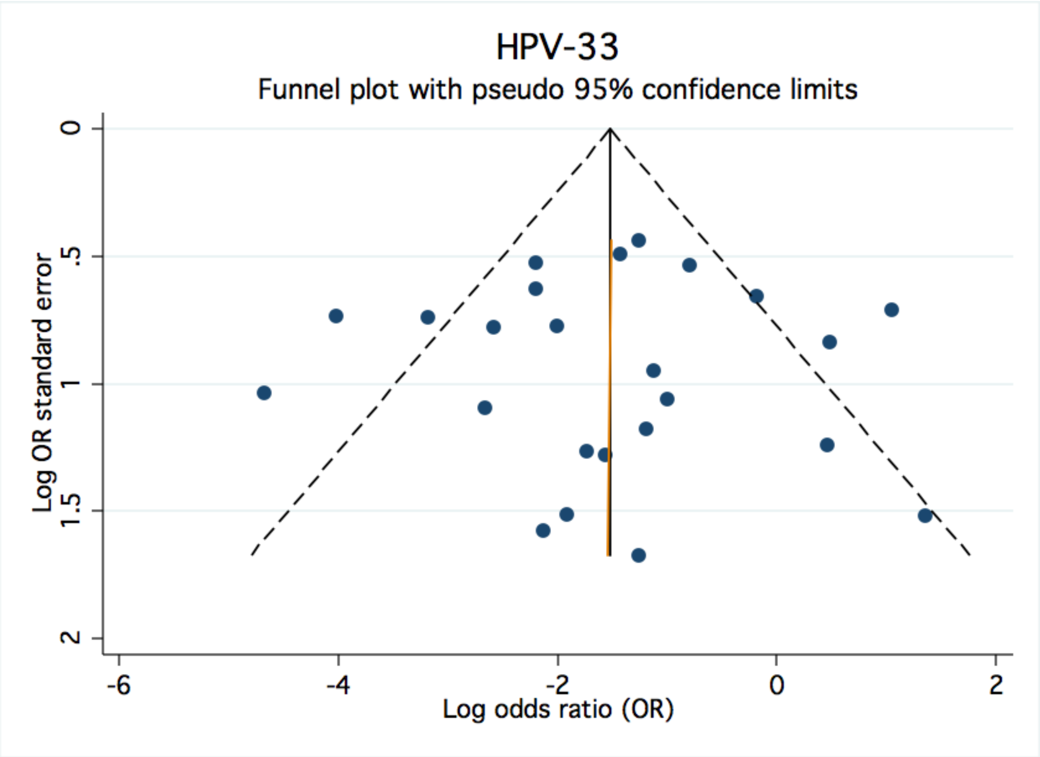
HPV-33
Funnel plot with pseudo 95% confidence limits
Log OR standard error
0
.5
1
1.5
2
-6
-4
-2
0
2
Log odds ratio (OR)

(g)

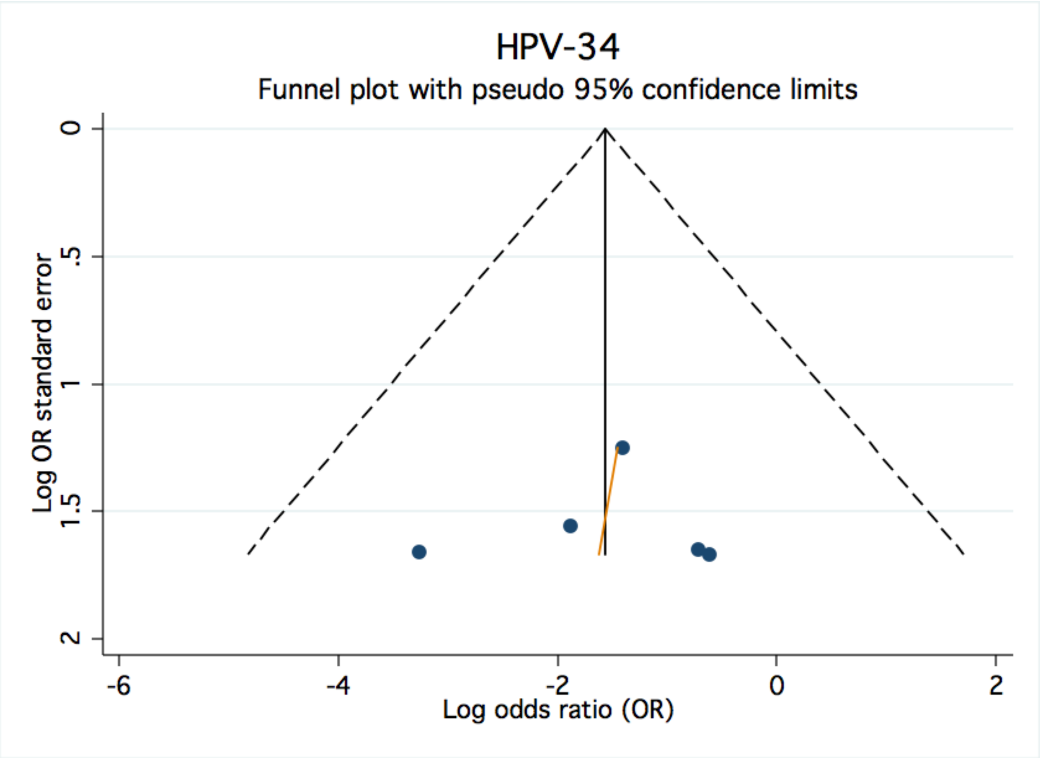
HPV-34
Funnel plot with pseudo 95% confidence limits
Log OR standard error
0
.5
1
1.5
2
-6
-4
-2
0
2
Log odds ratio (OR)

(h)

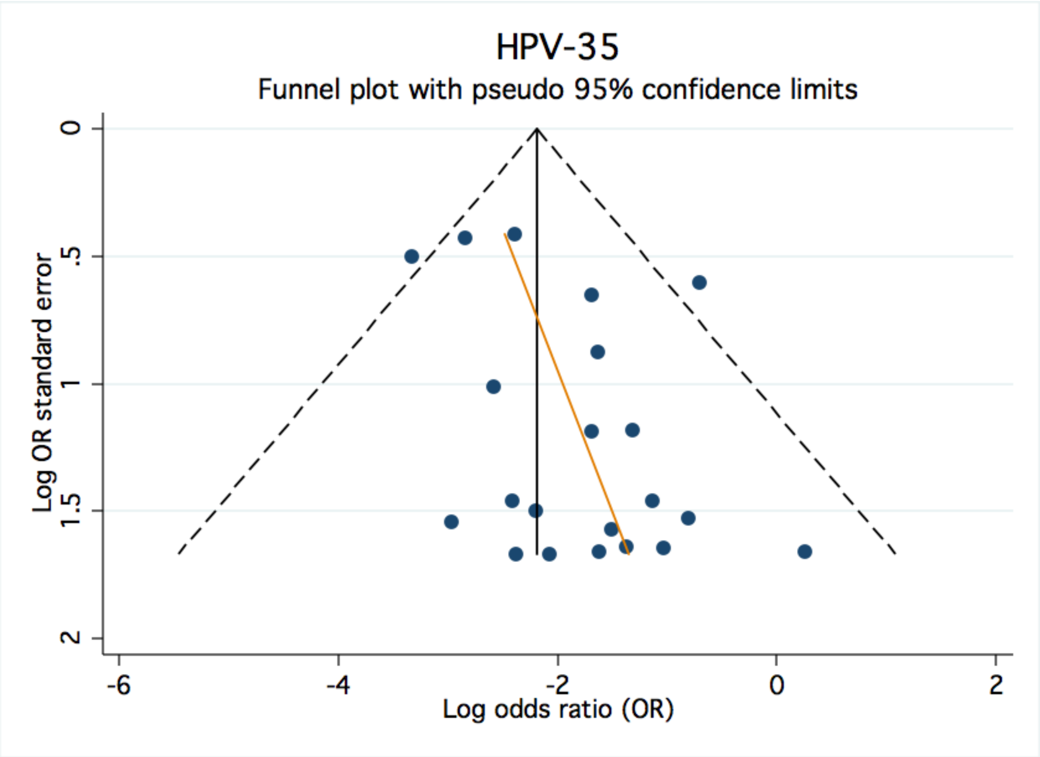
HPV-35
Funnel plot with pseudo 95% confidence limits
Log OR standard error
0
.5
1
1.5
2
-6
-4
-2
0
2
Log odds ratio (OR)

(i)

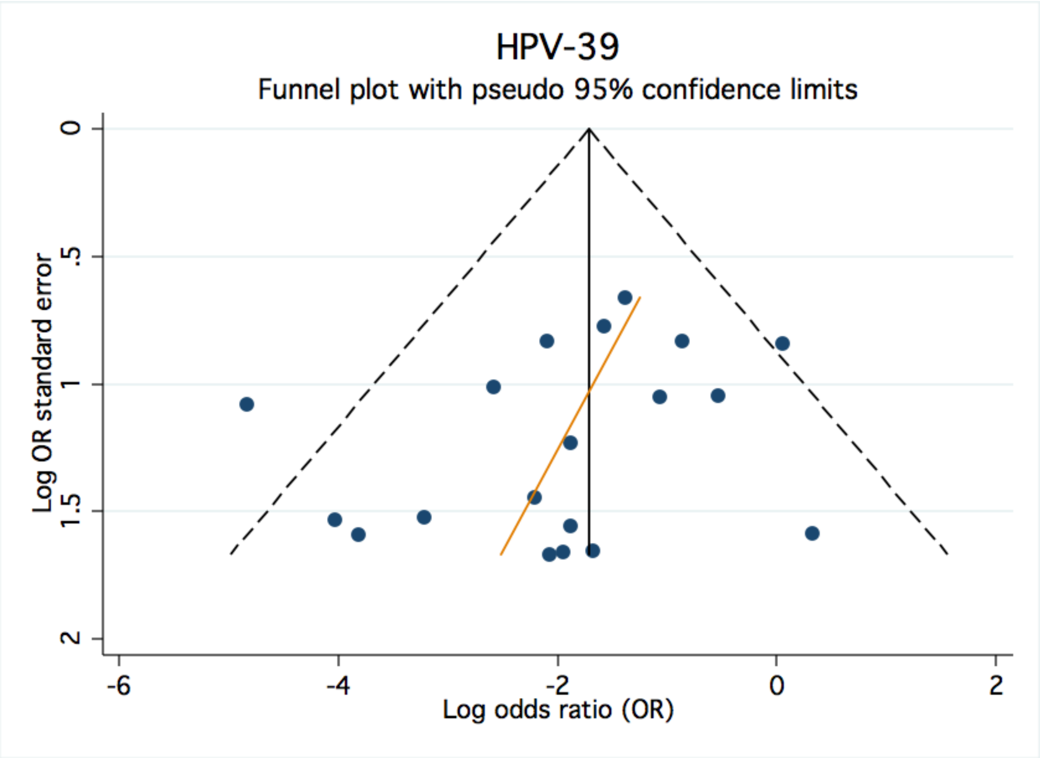
HPV-39
Funnel plot with pseudo 95% confidence limits
Log OR standard error
0
.5
1
1.5
2
-6
-4
-2
0
2
Log odds ratio (OR)

(j)

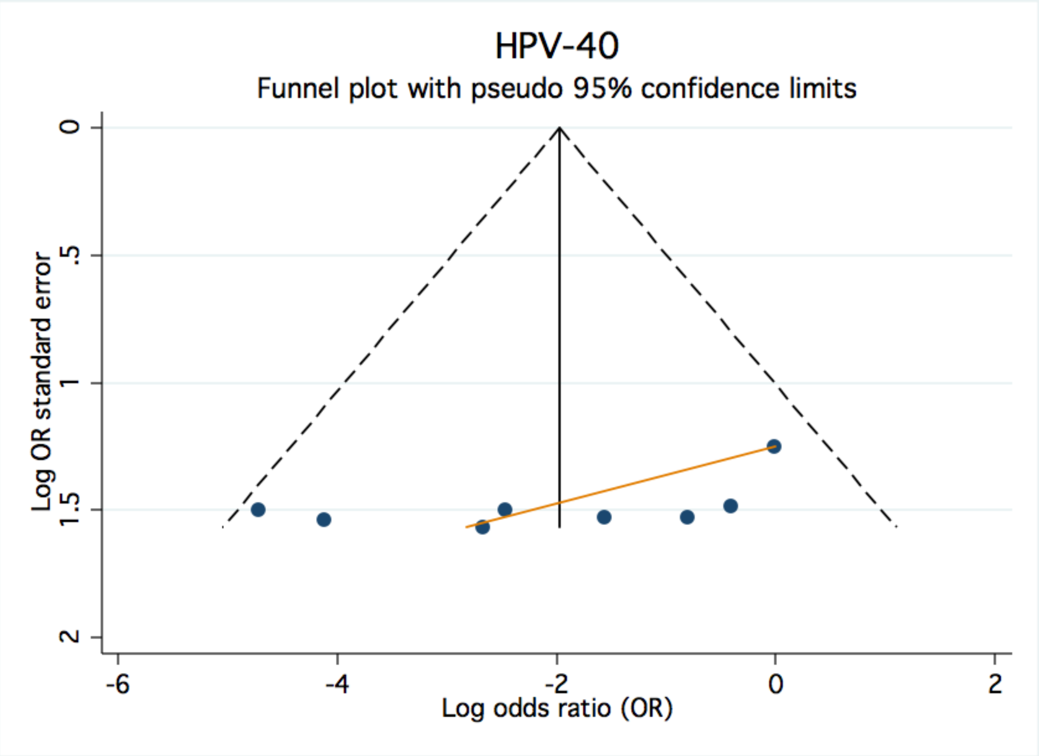
HPV-40
Funnel plot with pseudo 95% confidence limits
Log OR standard error
0
.5
1
1.5
2
-6
-4
-2
0
2
Log odds ratio (OR)

(k)

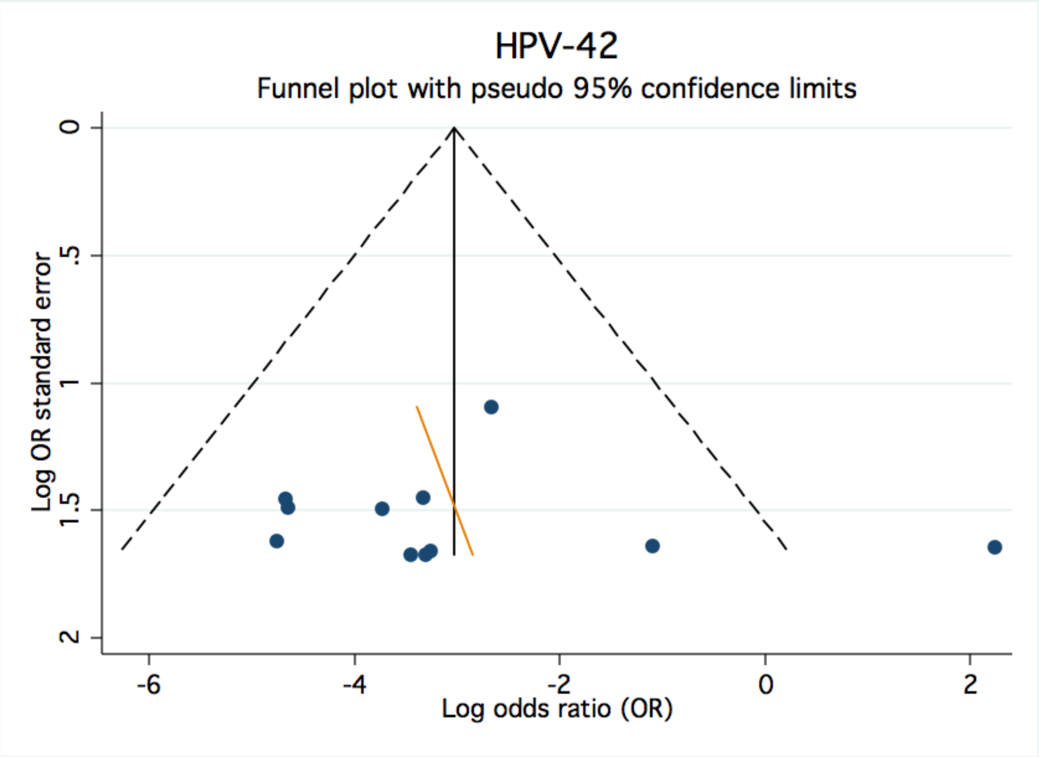
HPV-42
Funnel plot with pseudo 95% confidence limits
Log OR standard error
0
.5
1
1.5
2
-6
-4
-2
0
2
Log odds ratio (OR)

(l)

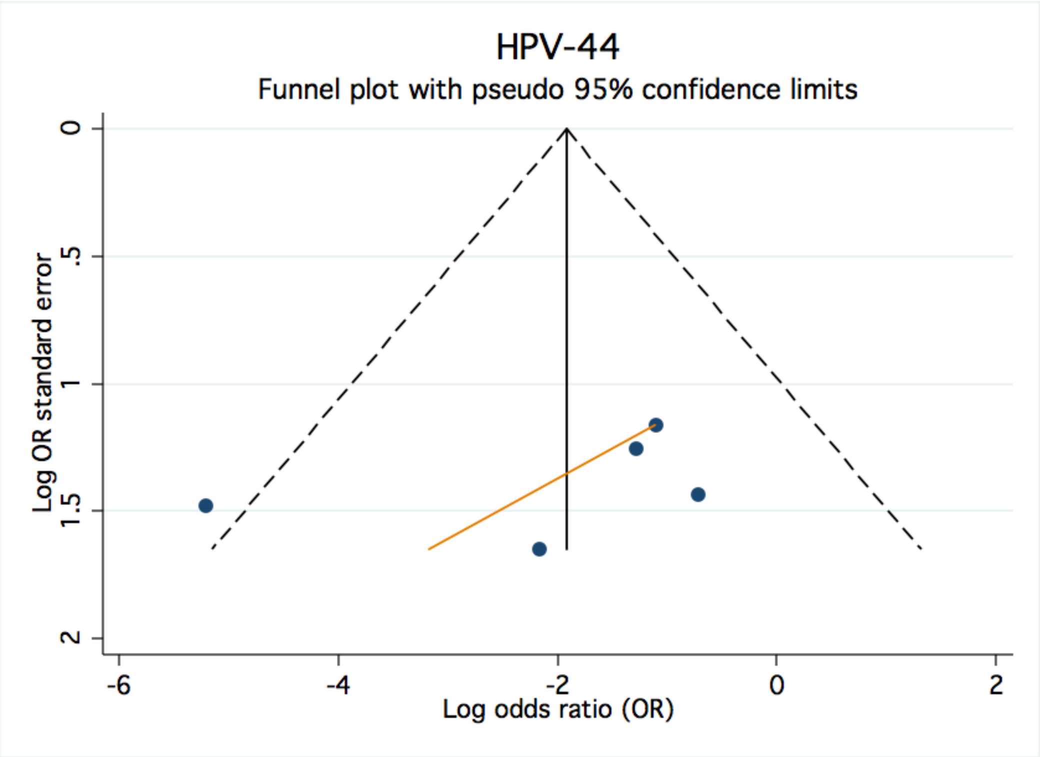
HPV-44
Funnel plot with pseudo 95% confidence limits
Log OR standard error
0
.5
1
1.5
2
-6
-4
-2
0
2
Log odds ratio (OR)

(m)

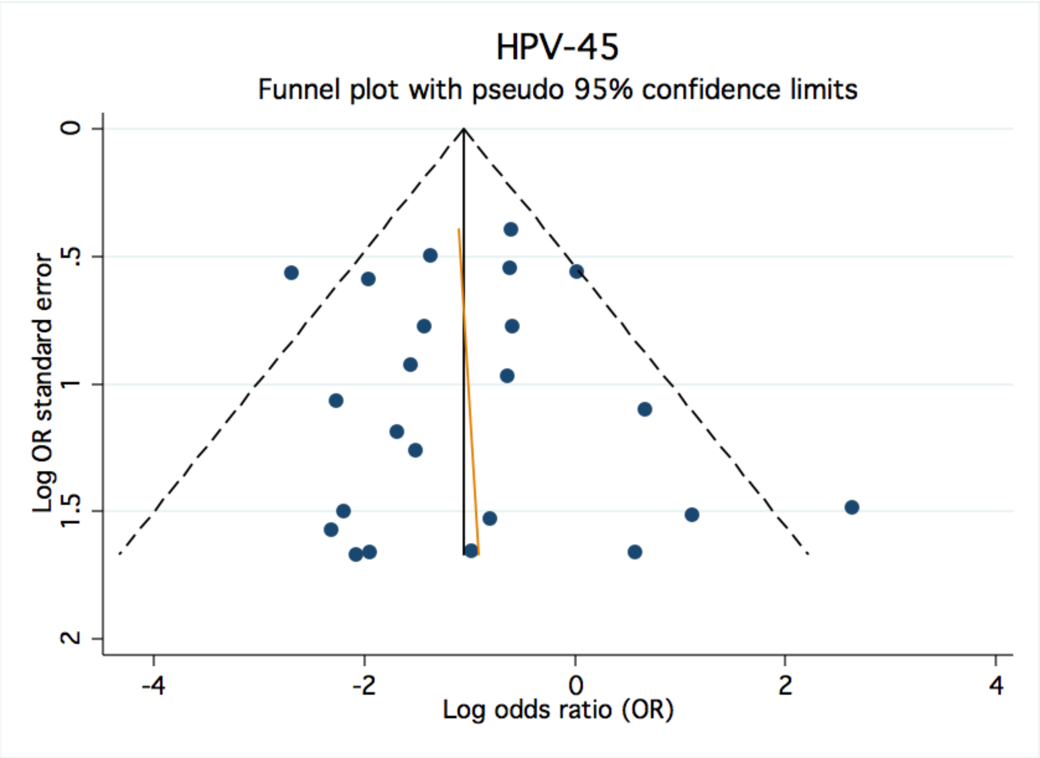
HPV-45
Funnel plot with pseudo 95% confidence limits
Log OR standard error
0
.5
1
1.5
2
-4
-2
0
2
4
Log odds ratio (OR)

(n)

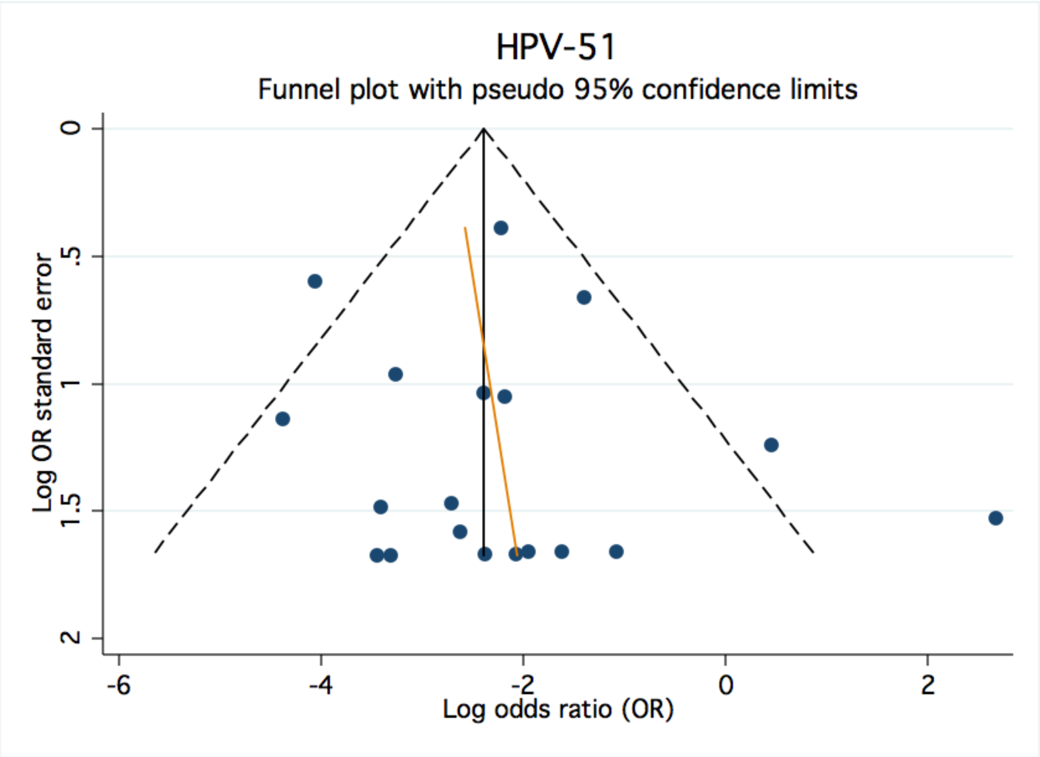
HPV-51
Funnel plot with pseudo 95% confidence limits
Log OR standard error
0
.5
1
1.5
2
-6
-4
-2
0
2
Log odds ratio (OR)

(o)

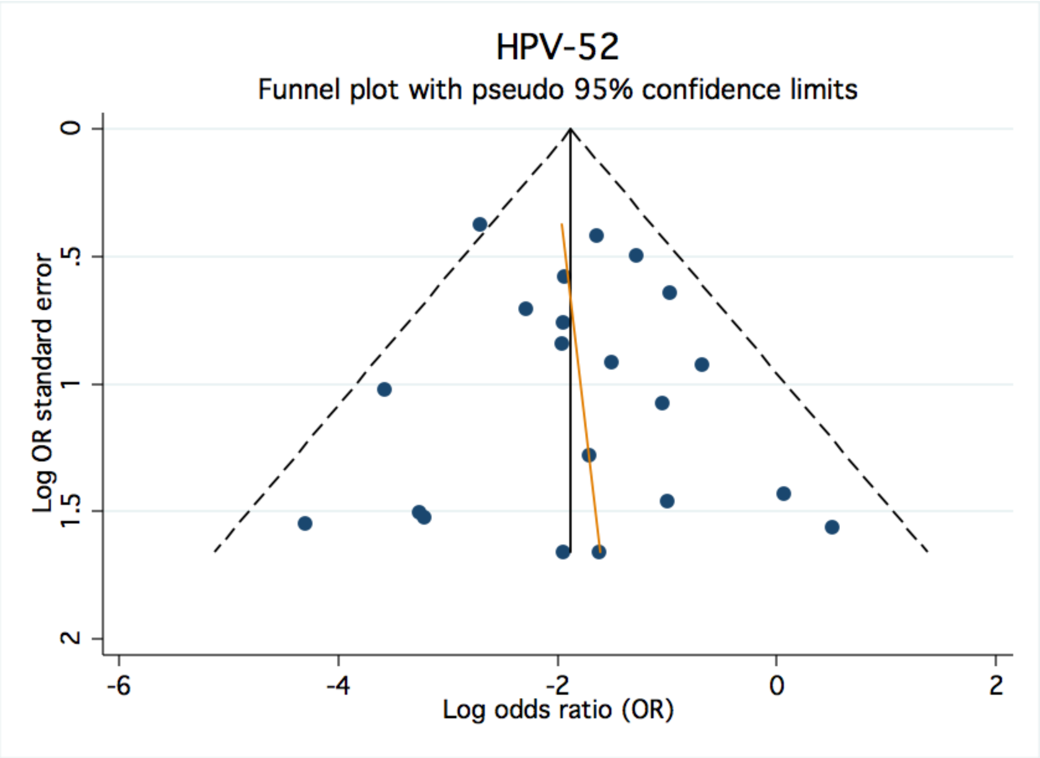
HPV-52
Funnel plot with pseudo 95% confidence limits
Log OR standard error
0
.5
1
1.5
2
-6
-4
-2
0
2
Log odds ratio (OR)

(p)

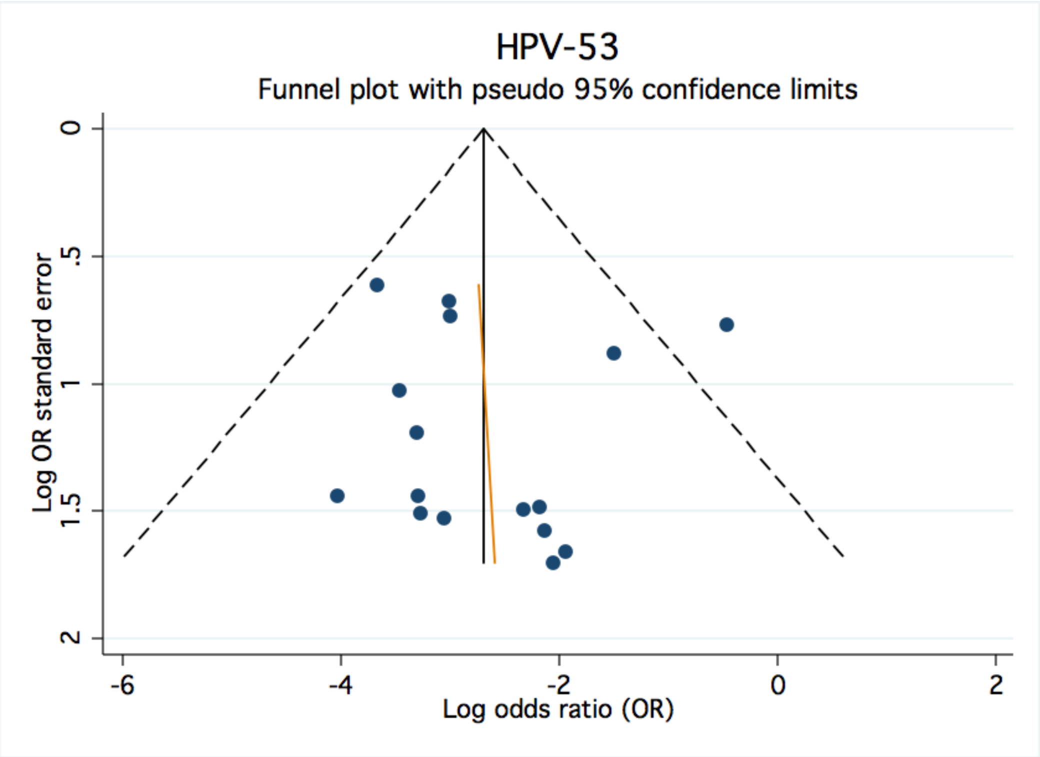
HPV-53
Funnel plot with pseudo 95% confidence limits
Log OR standard error
0
.5
1
1.5
2
-6
-4
-2
0
2
Log odds ratio (OR)

(q)

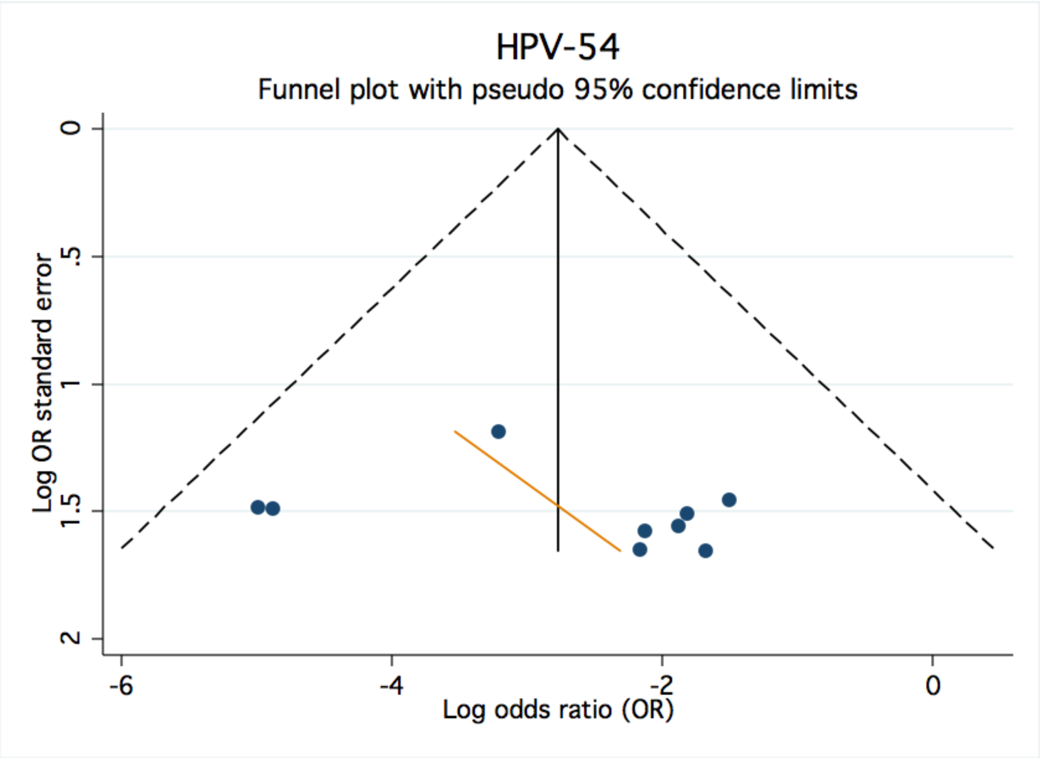
HPV-54
Funnel plot with pseudo 95% confidence limits
Log OR standard error
0
.5
1
1.5
2
-6
-4
-2
0
Log odds ratio (OR)

(r)

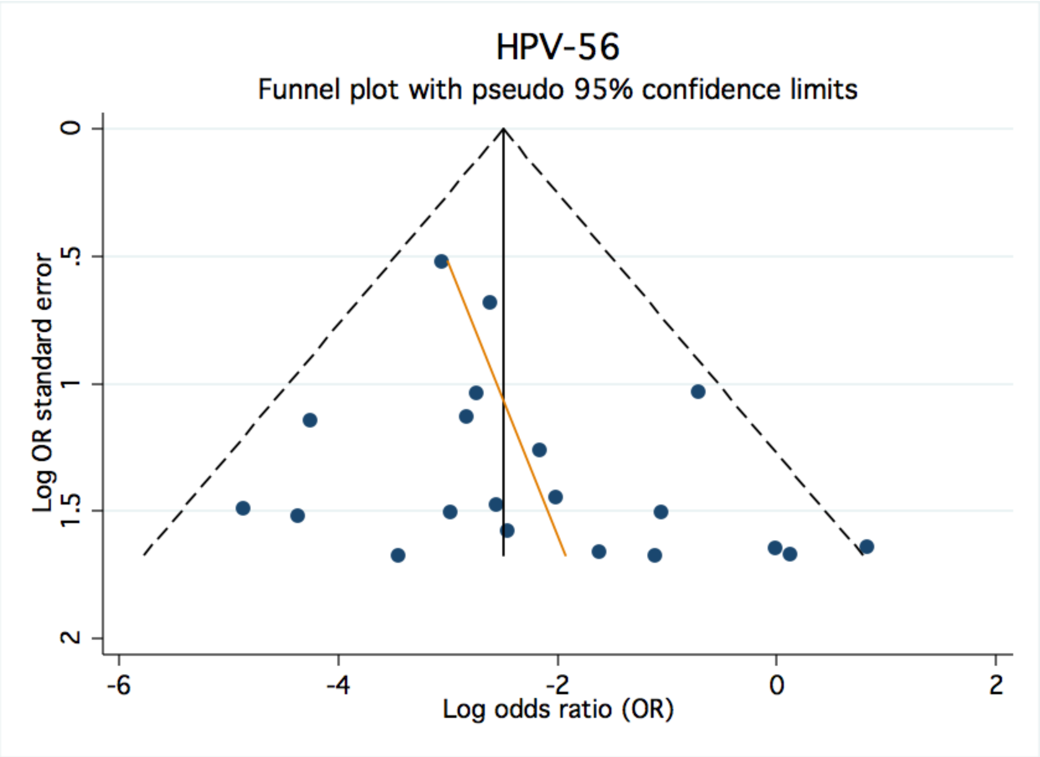
HPV-56
Funnel plot with pseudo 95% confidence limits
Log OR standard error
0
.5
1
1.5
2
-6
-4
-2
0
2
Log odds ratio (OR)

(s)

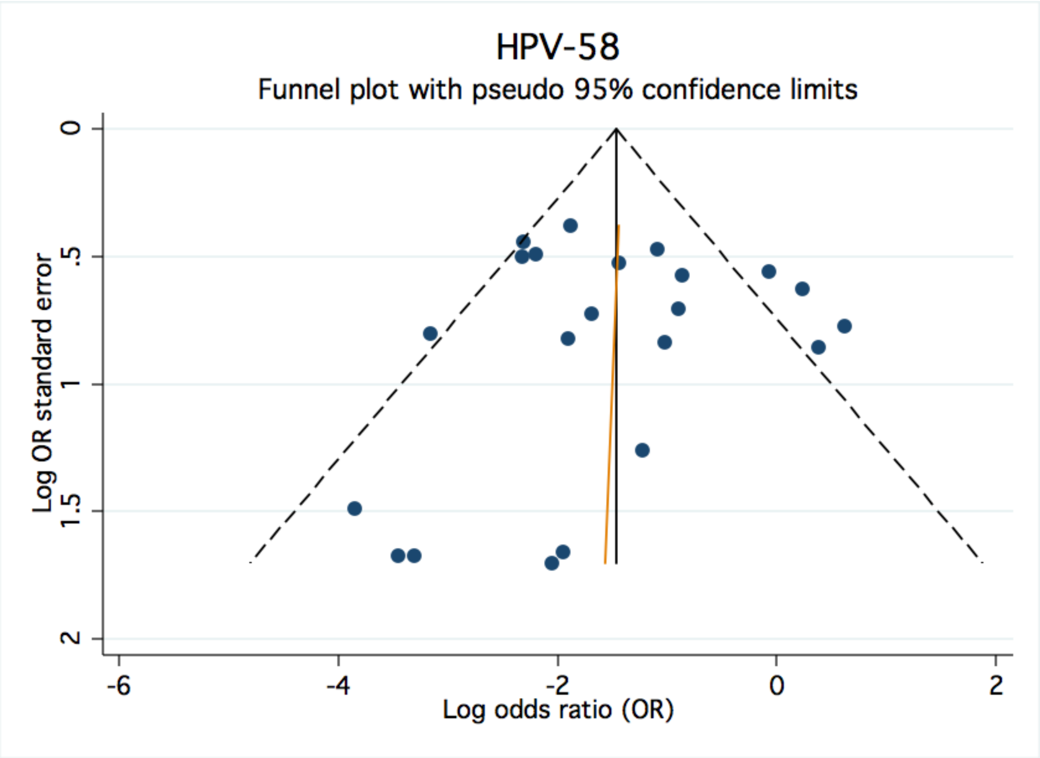
HPV-58
Funnel plot with pseudo 95% confidence limits
Log OR standard error
0
.5
1
1.5
2
-6
-4
-2
0
2
Log odds ratio (OR)

(t)

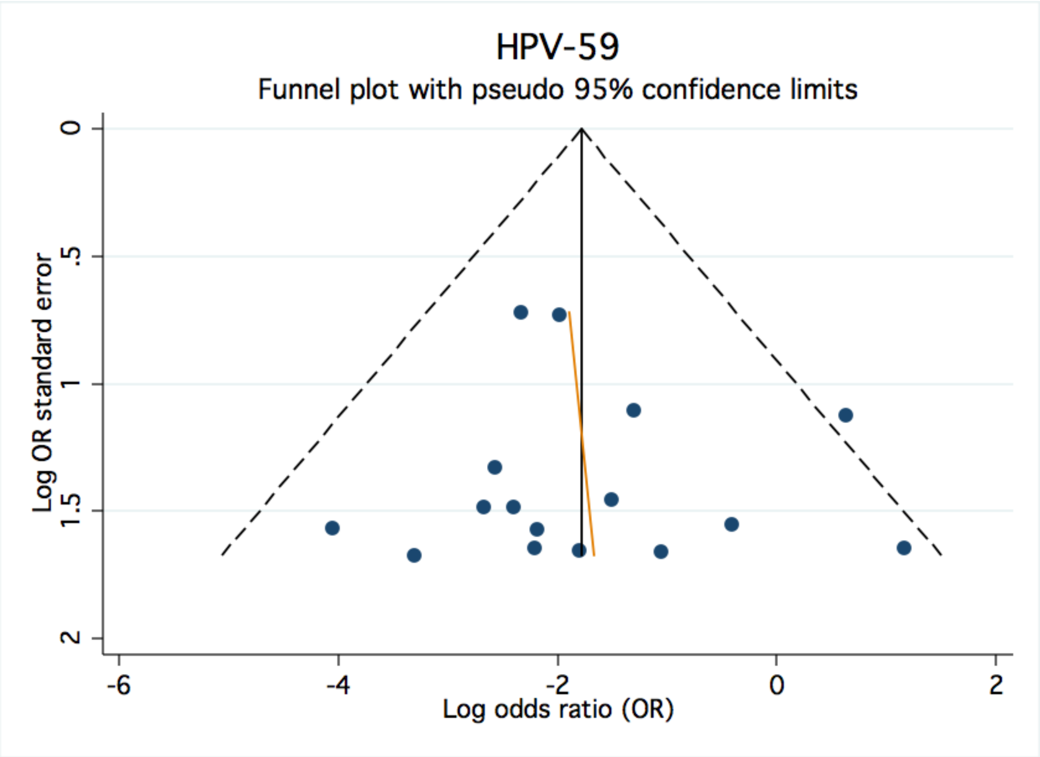
HPV-59
Funnel plot with pseudo 95% confidence limits
Log OR standard error
0
.5
1
1.5
2
-6
-4
-2
0
2
Log odds ratio (OR)

(u)

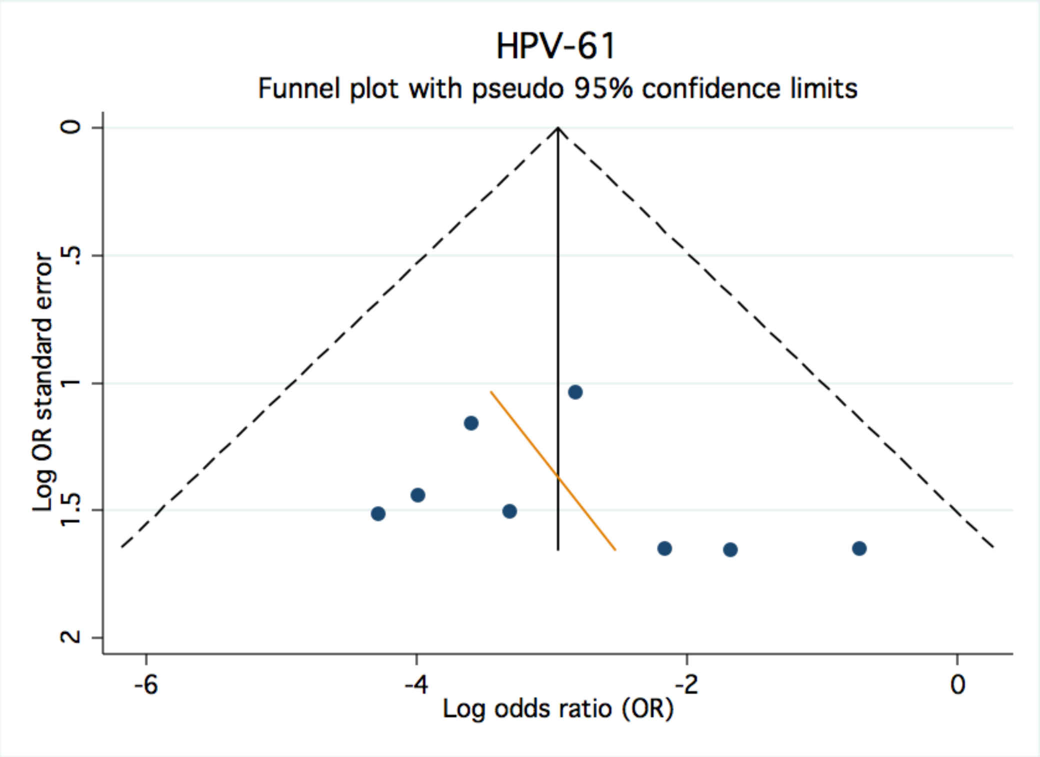
HPV-61
Funnel plot with pseudo 95% confidence limits
Log OR standard error
0
.5
1
1.5
2
-6
-4
-2
0
Log odds ratio (OR)

(v)

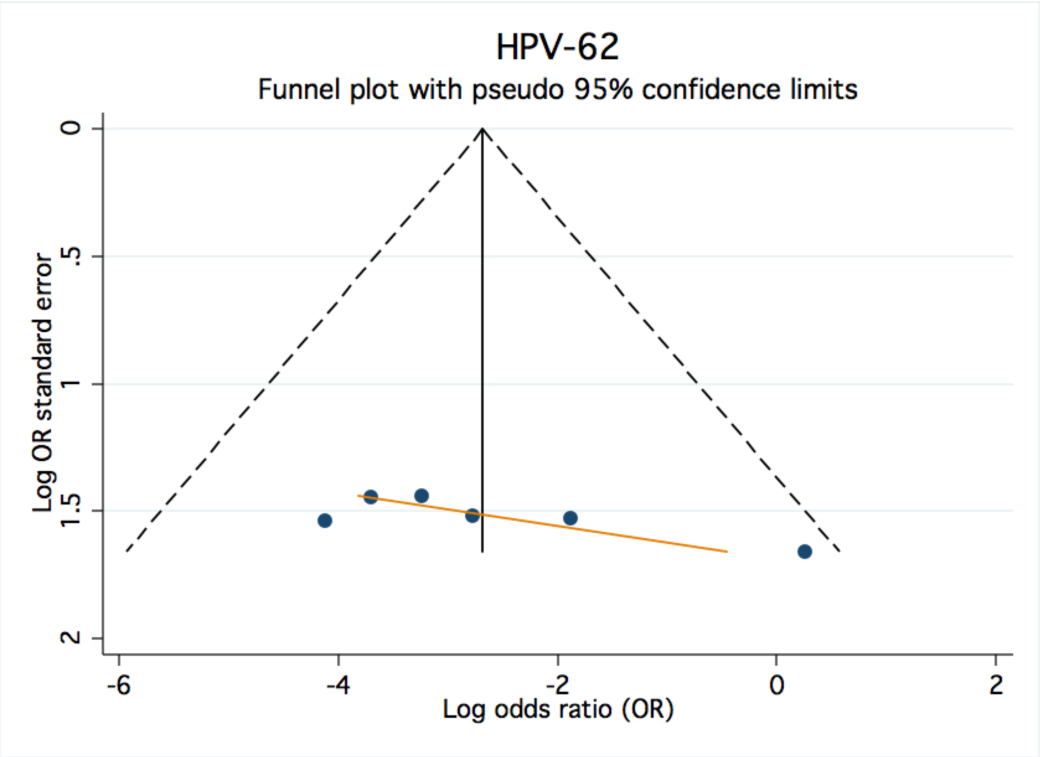
HPV-62
Funnel plot with pseudo 95% confidence limits
Log OR standard error
0
.5
1
1.5
2
-6
-4
-2
0
2
Log odds ratio (OR)

(w)

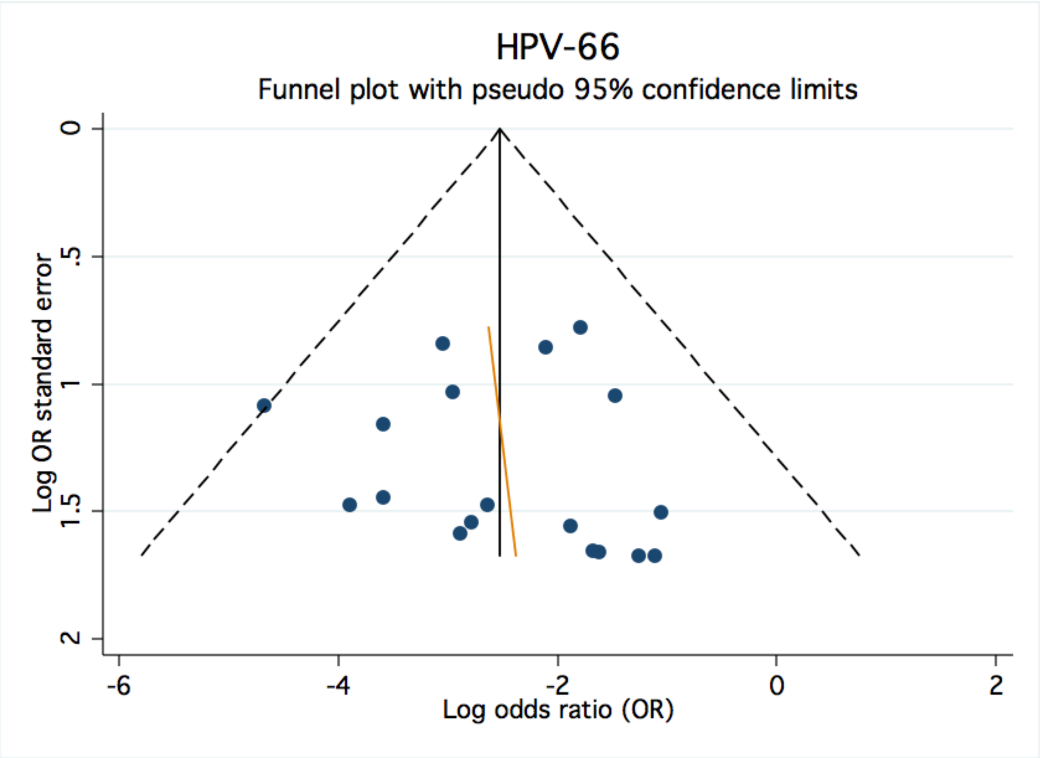
HPV-66
Funnel plot with pseudo 95% confidence limits
Log OR standard error
0
.5
1
1.5
2
-6
-4
-2
0
2
Log odds ratio (OR)

(x)

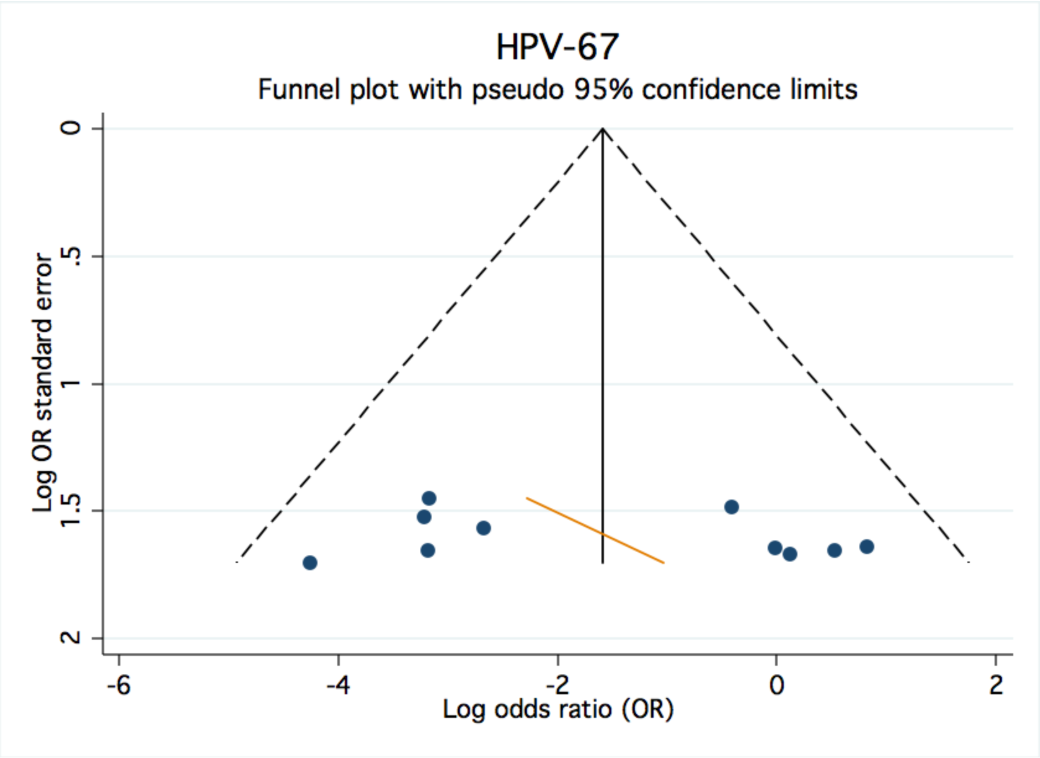
HPV-67
Funnel plot with pseudo 95% confidence limits
Log OR standard error
0
.5
1
1.5
2
-6
-4
-2
0
2
Log odds ratio (OR)

(y)

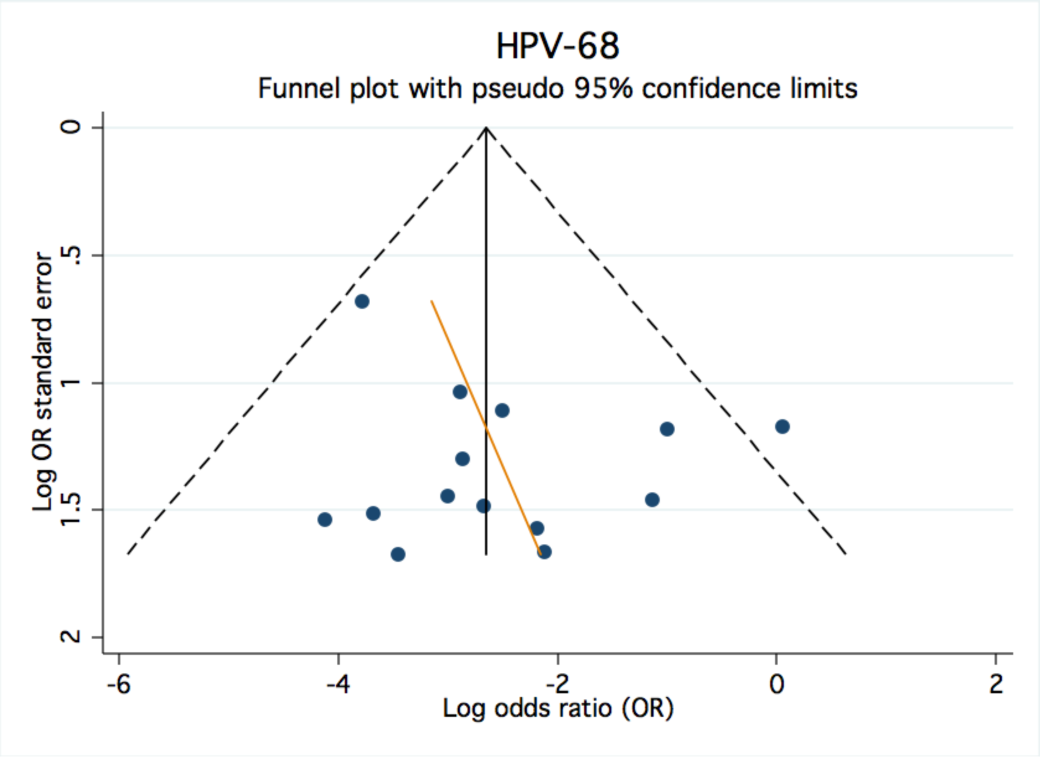
HPV-68
Funnel plot with pseudo 95% confidence limits
Log OR standard error
0
.5
1
1.5
2
-6
-4
-2
0
2
Log odds ratio (OR)

(z)

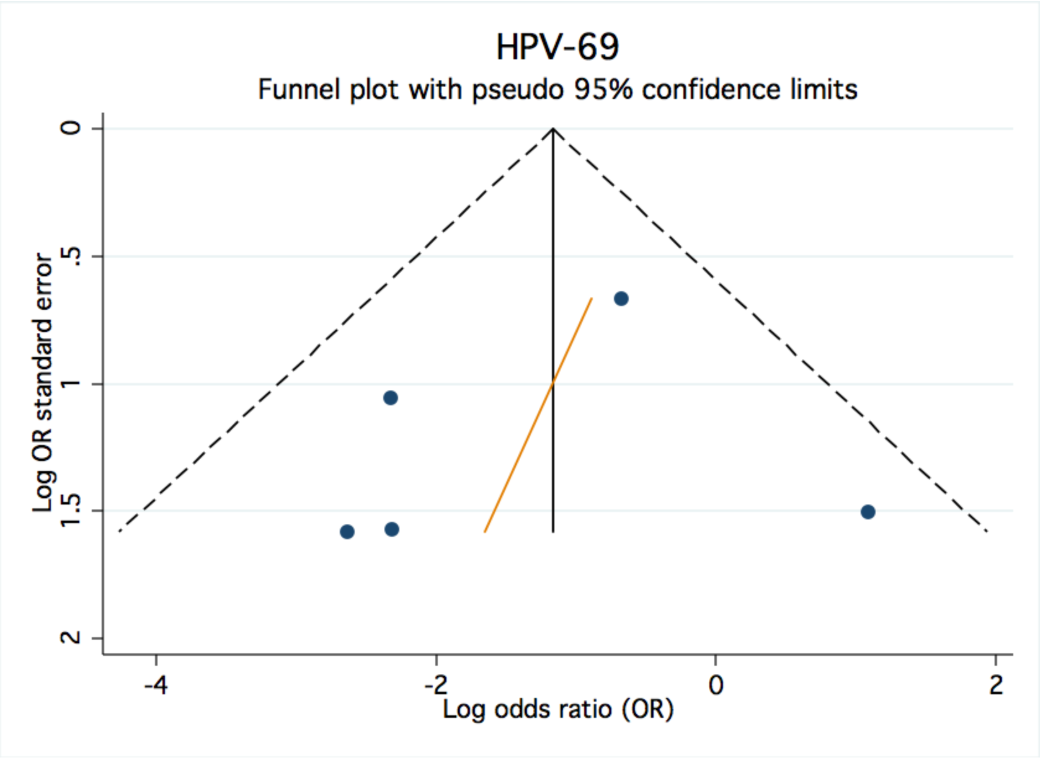
HPV-69
Funnel plot with pseudo 95% confidence limits
Log OR standard error
0
.5
1
1.5
2
-4
-2
0
2
Log odds ratio (OR)

(aa)

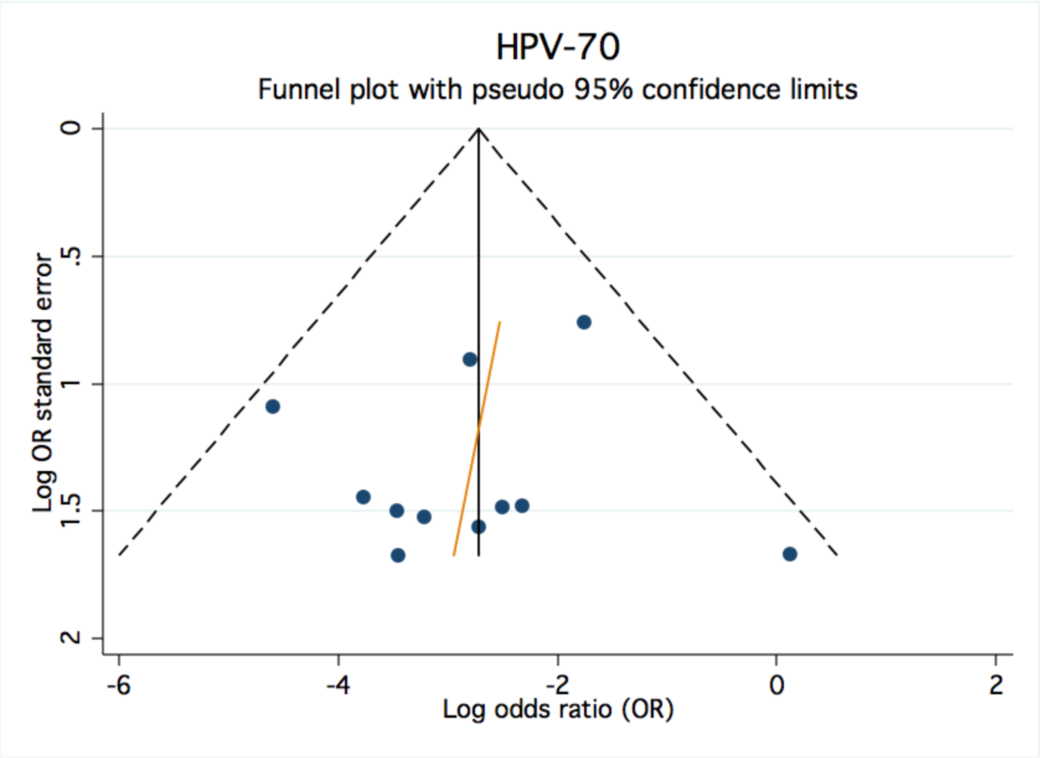
HPV-70
Funnel plot with pseudo 95% confidence limits
Log OR standard error
0
.5
1
1.5
2
-6
-4
-2
0
2
Log odds ratio (OR)

(ab)

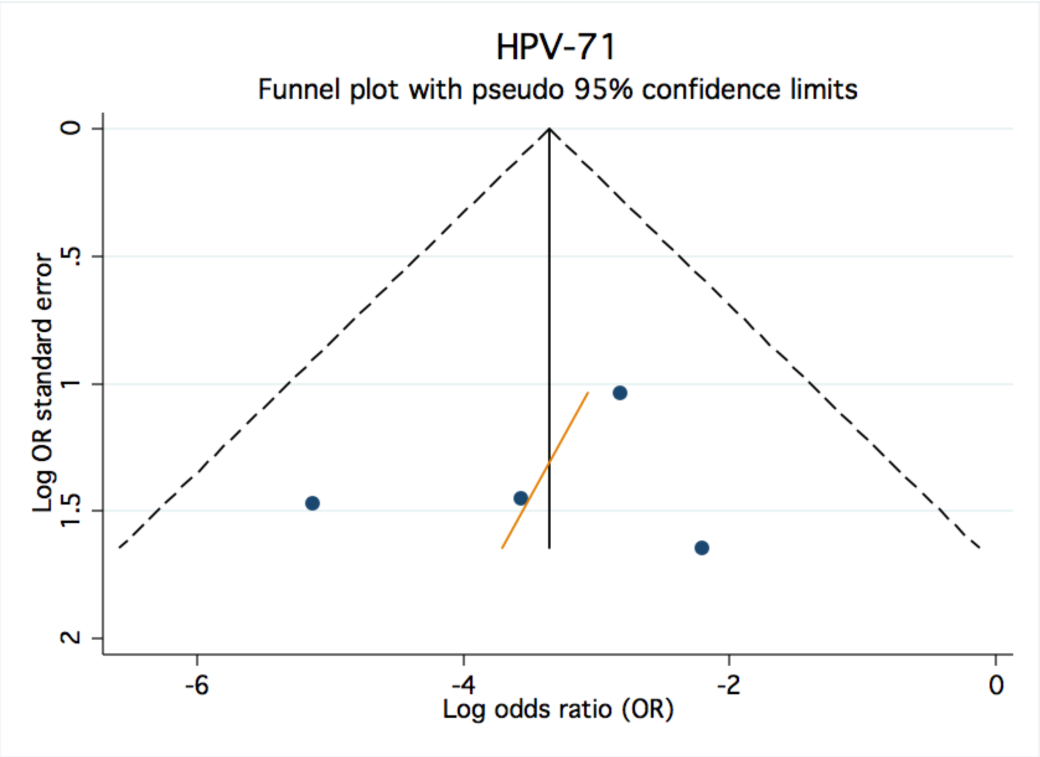
HPV-71
Funnel plot with pseudo 95% confidence limits
Log OR standard error
0
.5
1
1.5
2
-6
-4
-2
0
Log odds ratio (OR)

(ac)

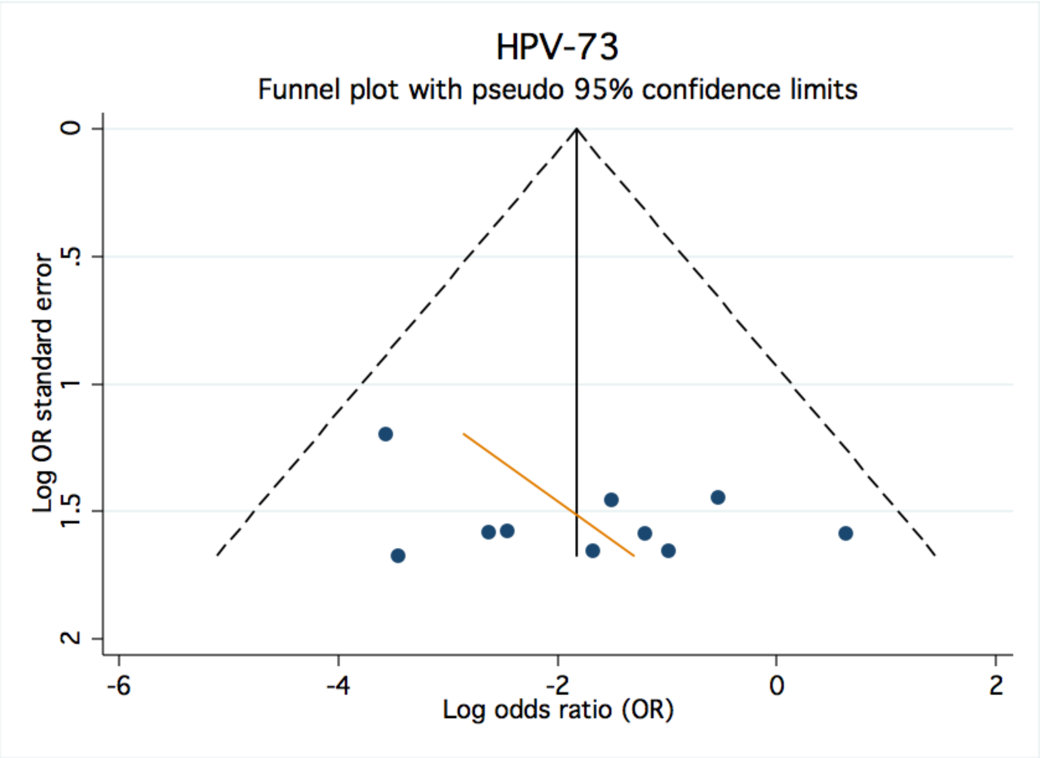
HPV-73
Funnel plot with pseudo 95% confidence limits
Log OR standard error
0
.5
1
1.5
2
-6
-4
-2
0
2
Log odds ratio (OR)

(ad)

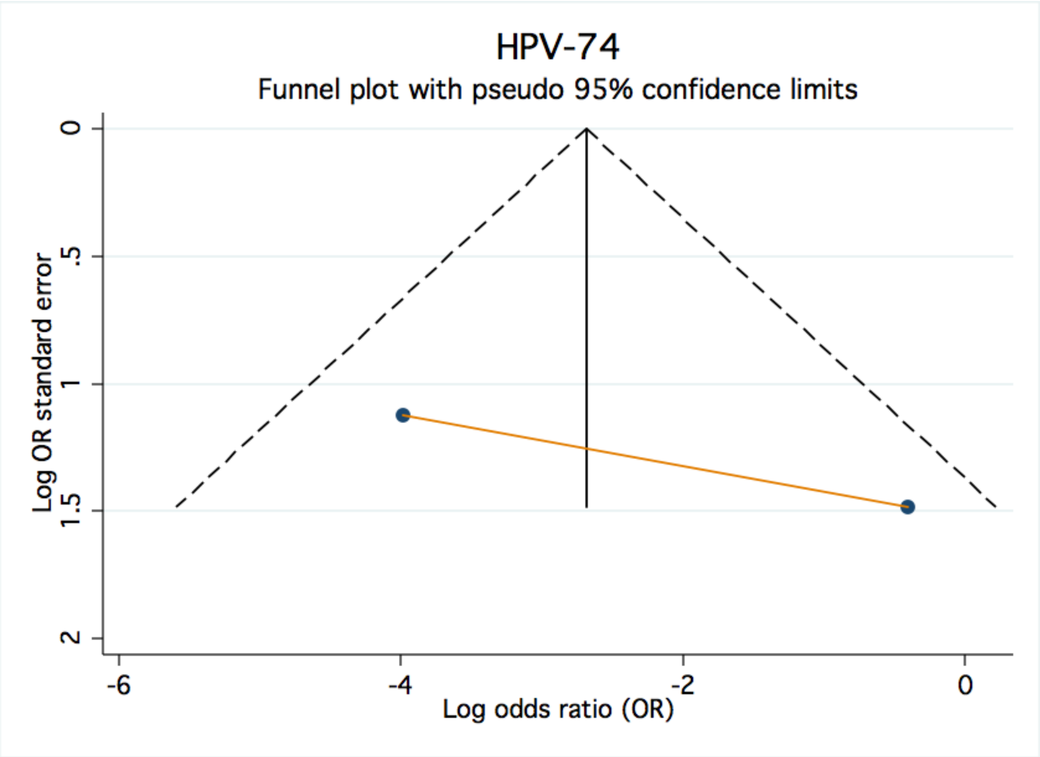
HPV-74
Funnel plot with pseudo 95% confidence limits
Log OR standard error
0
.5
1
1.5
2
-6
-4
-2
0
Log odds ratio (OR)

(ae)

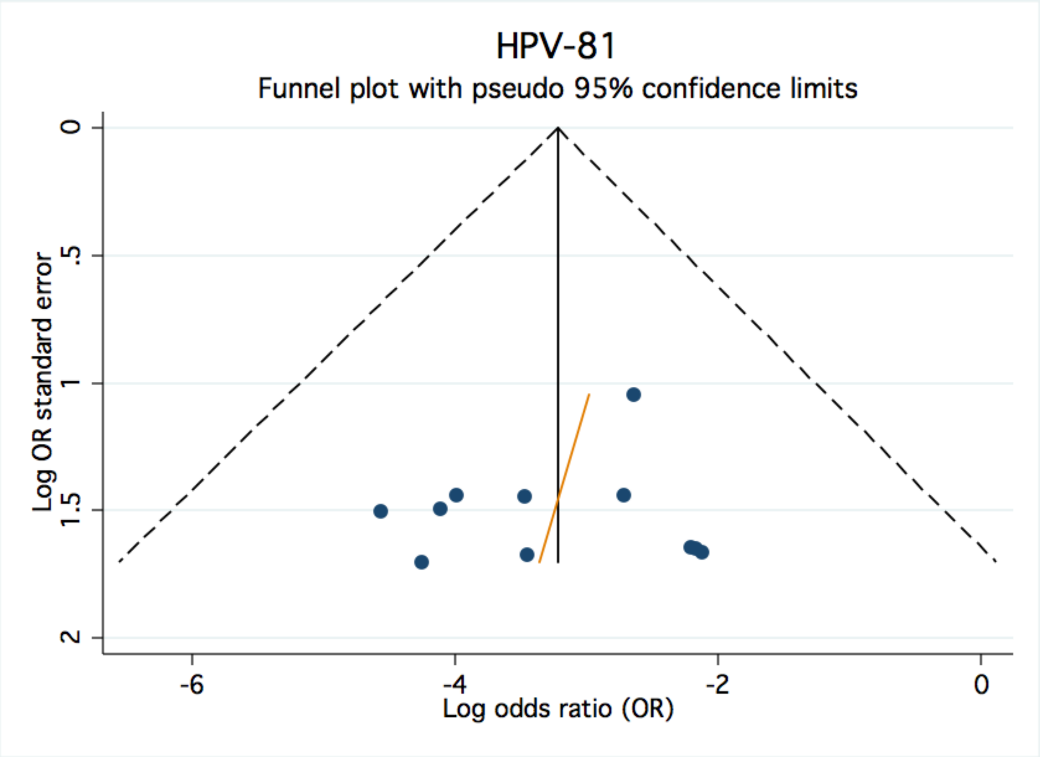
HPV-81
Funnel plot with pseudo 95% confidence limits
Log OR standard error
0
.5
1
1.5
2
-6
-4
-2
0
Log odds ratio (OR)

(af)

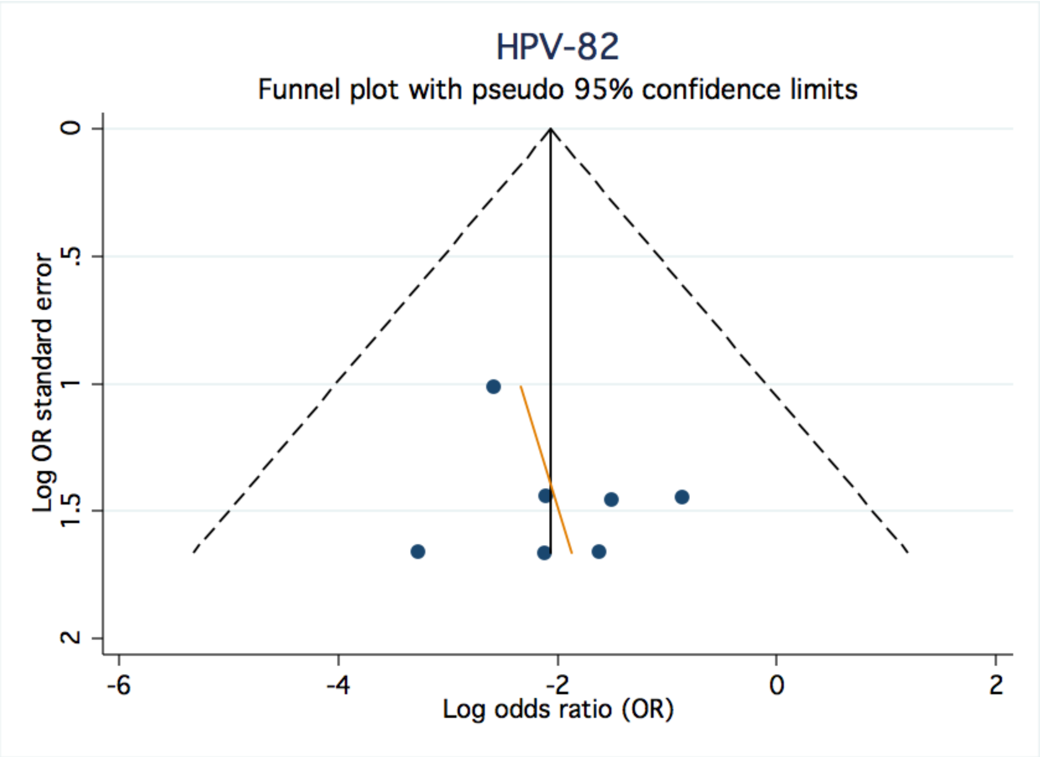
HPV-82
Funnel plot with pseudo 95% confidence limits
Log OR standard error
0
.5
1
1.5
2
-6
-4
-2
0
2
Log odds ratio (OR)
