# Supplementary material for: Comparing human papillomavirus prevalences in women with normal cytology or invasive cervical cancer to rank genotypes according to their oncogenic potential: a meta-analysis of observational studies
Source: BMC Infect Dis. 2013 Aug 13;13:373. doi: 10.1186/1471-2334-13-373 (PMC3751808; doi:10.1186/1471-2334-13-373)
Supplement: Additional file 4 — Sensitivity analyses of human papillomavirus genotype ranking. *Analyses using a fixed-effect model [27]. †Analyses using DerSimonian and Laird’s random-effects model [20] with a continuity correction (CC) = 0.25. ‡Analyses using DerSimonian and Laird’s random-effects model [20] with CC = 0. HPV-62 and -69 ORs could not be calculated. §Analyses using DerSimonian and Laird’s random-effects model [20] (CC = 0.5), with HPV-negative subjects as the reference group. In this model, unlike the preceding ones, the pooled OR for HPV-16 could be estimated. Abbreviations: HPV, human papilloma virus; OR, odds ratio; CI, confidence interval. [file 1471-2334-13-373-S4.pdf]

Additional file 4: Sensitivity analyses of human papillomavirus genotype ranking

Rank	Peto's method*			Continuity correction = 0.25†			Continuity correction = 0‡			HPV-negative§		
	HPV-	Pooled OR	95% CI	HPV-	Pooled OR	95% CI	HPV-	Pooled OR	95% CI	HPV-	Pooled OR	95% CI
1	18	0.65	0.52, 0.80	18	0.63	0.50, 0.78	40	0.98	0.08, 11.36	16	136.7	70.0, 266.9
2	45	0.36	0.26, 0.49	45	0.33	0.21, 0.53	18	0.62	0.50, 0.77	18	99.1	49.8, 197.2
3	69	0.32	0.15, 0.67	69	0.26	0.07, 1.00	44	0.35	0.08, 1.46	67	89.8	13.6, 593.6
4	58	0.23	0.18, 0.29	58	0.25	0.16, 0.40	45	0.32	0.20, 0.52	69	81.0	5.0, 1,313.3
5	31	0.19	0.14, 0.25	33	0.21	0.12, 0.38	58	0.28	0.17, 0.45	45	70.5	34.3, 144.8
6	33	0.18	0.14, 0.23	31	0.21	0.13, 0.34	69	0.27	0.05, 1.38	73	48.2	11.1, 209.5
7	30	0.16	0.05, 0.53	67	0.17	0.04, 0.77	34	0.24	0.02, 2.84	31	46.5	18.8, 115.3
8	52	0.16	0.12, 0.21	39	0.17	0.09, 0.31	33	0.20	0.11, 0.37	33	39.5	16.4, 94.9
9	67	0.15	0.07, 0.34	34	0.16	0.03, 0.80	59	0.20	0.07, 0.57	58	35.0	14.9, 82.4
10	39	0.14	0.09, 0.22	59	0.16	0.08, 0.31	39	0.20	0.10, 0.40	82	34.3	6.8, 172.3
11	59	0.13	0.07, 0.25	52	0.16	0.11, 0.23	31	0.19	0.12, 0.32	59	33.4	14.3, 78.2
12	73	0.12	0.05, 0.32	44	0.15	0.03, 0.80	30	0.18	0.02, 1.54	52	25.2	11.1, 57.2
13	34	0.11	0.03, 0.50	73	0.13	0.04, 0.42	52	0.16	0.11, 0.24	35	21.0	10.0, 44.1
14	62	0.11	0.06, 0.21	30	0.12	0.02, 0.66	82	0.13	0.03, 0.53	56	20.6	6.8, 63.0
15	11	0.11	0.07, 0.16	82	0.11	0.03, 0.35	6	0.12	0.05, 0.29	39	18.0	6.6, 48.8
16	53	0.10	0.08, 0.14	40	0.10	0.03, 0.42	11	0.12	0.05, 0.26	51	16.2	7.0, 37.9
17	35	0.10	0.07, 0.14	35	0.10	0.07, 0.15	73	0.12	0.01, 2.28	61	16.1	4.9, 53.1
18	66	0.10	0.07, 0.15	11	0.10	0.06, 0.18	35	0.11	0.06, 0.20	44	14.9	0.7, 304.6
19	81	0.09	0.06, 0.15	51	0.09	0.04, 0.18	68	0.10	0.03, 0.33	40	13.7	1.5, 125.4
20	51	0.09	0.07, 0.13	6	0.08	0.04, 0.16	51	0.08	0.03, 0.20	30	13.4	2.8, 63.8
21	82	0.09	0.03, 0.28	56	0.07	0.04, 0.12	53	0.07	0.03, 0.18	68	12.3	4.3, 35.3
22	6	0.08	0.06, 0.13	66	0.07	0.04, 0.13	81	0.07	0.01, 0.55	74	12.2	0.0, 7,404.2
23	68	0.08	0.05, 0.13	53	0.06	0.04, 0.11	42	0.07	0.01, 0.60	11	11.2	2.7, 46.5
24	70	0.08	0.05, 0.13	68	0.06	0.03, 0.13	66	0.07	0.03, 0.15	34	11.1	1.2, 106.0
25	56	0.07	0.05, 0.11	70	0.06	0.02, 0.13	56	0.07	0.03, 0.12	71	10.4	0.9, 117.3
26	61	0.07	0.04, 0.13	74	0.05	0.00, 0.91	71	0.06	0.01, 0.45	6	9.4	4.0, 21.8
27	71	0.07	0.03, 0.13	62	0.04	0.01, 0.23	70	0.05	0.01, 0.26	66	8.5	2.7, 26.6
28	40	0.05	0.02, 0.13	61	0.04	0.01, 0.12	61	0.04	0.01, 0.19	42	8.4	3.4, 20.7
29	42	0.05	0.03, 0.09	54	0.04	0.01, 0.13	54	0.04	0.00, 0.41	54	8.2	1.2, 55.0
30	44	0.04	0.02, 0.12	42	0.03	0.01, 0.12	74	0.02	0.00, 0.17	53	8.0	2.6, 23.9
31	54	0.02	0.01, 0.05	71	0.03	0.01, 0.15				70	7.7	1.4, 42.4
32	74	0.02	0.00, 0.08	81	0.03	0.01, 0.09				62	7.4	1.9, 29.5
33										81	6.6	1.7, 25.3

*Analyses using a fixed-effect model [27]. †Analyses using DerSimonian and Laird's random-effects model [20] with a continuity correction (CC) = 0.25. ‡Analyses using DerSimonian and Laird's random-effects model [20] with CC = 0. HPV-62 and -69 ORs could not be calculated. §Analyses using DerSimonian and Laird's random-effects model [20] (CC = 0.5), with HPV-negative subjects as the reference group. In this model, unlike the preceding ones, the pooled OR for HPV-16 could be estimated. Abbreviations: HPV, human papilloma virus; OR, odds ratio; CI, confidence interval.
